# Parental Reflective Capacities: A Scoping Review of Mindful Parenting and Parental Reflective Functioning

**DOI:** 10.1007/s12671-024-02379-6

**Published:** 2024-06-18

**Authors:** Tuyen Huynh, Margaret L. Kerr, Christina N. Kim, Endang Fourianalistyawati, Vickie Ya-Rong Chang, Larissa G. Duncan

**Affiliations:** 1 Department of Psychology, University of South Carolina, Barnwell College Room 517, 1512 Pendleton Street, Columbia, SC 29208, USA; 2 Human Development and Family Studies, University of Wisconsin-Madison, 4109 Nancy Nicholas Hall, 1300 Linden Drive, Madison, Wisconsin 53706, USA; 3 Department of Population Health, NYU Grossman School of Medicine, New York, USA; 4 Faculty of Psychology, Universitas YARSI, Menara YARSI, Kav. 13, Jl. Letjend. Suprapto. Cempaka Putih, Jakarta Pusat, DKI, Jakarta 10510, Indonesia; 5 1400 Shattuck Ave, Ste 12, PO Box 178, Berkeley, California 94709, USA; 6 Human Development and Family Studies, University of Wisconsin-Madison, 1300 Linden Drive, Madison, Wisconsin 53706, USA

**Keywords:** Mindful parenting, Mindfulness, Parental reflective functioning, Parenting

## Abstract

**Objectives:**

Two key parental reflective capacities—mindful parenting (MP) and parental reflective functioning (PRF) — have been shown to promote healthy parent-child relationships through parents’ increased sensitivity and responsiveness to their children’s needs in spite of parenting stressors. Despite the theoretical overlap between these two constructs, researchers have continued to examine them independently. Therefore, the purpose of this scoping review was to review the overlapping and distinctive outcomes and correlates in the empirical MP and PRF literatures.

**Method:**

A comprehensive literature search across the MP and PRF literature for studies published from 2005 through early 2020 (pre-COVID-19 pandemic) was conducted.

**Results:**

A review of 301 articles (*n* = 180 MP and *n* = 121 PRF) revealed overlapping study outcomes and correlates, including improvement in parent and child well-being, parenting behaviors, and attachment. Both MP and PRF literatures suggest MP and PRF are amenable to intervention-induced changes, although mostly documented in White mothers, which results may not be generalizable to diverse populations.

**Conclusions:**

Researchers should consider the impact MP and PRF have on positive family relationships. Results suggest that scholars should consider investigating and intervening on MP and PRF simultaneously. Specifically, results identified MP and PRF convergent associations and perhaps synergistic impacts on positive parenting behaviors. Limitations and future directions are discussed.

**Preregistration:**

This review was not preregistered.

The parent-child relationship is dynamic, and the quality of interactions is critical for early social, emotional, and cognitive development (Keller, 2018; Knauer et al., 2019). Evidence provides support for the important role parenting behaviors and caregiver interactions have on the quality of parent-child relationships (Brody et al., 2005). Mindful parenting and parental reflective functioning are two distinct parenting-related concepts that share theoretical and conceptual overlaps as well as key differences. Over the years, researchers have documented the associations of mindful parenting and parental reflective functioning, independently, with positive parenting (Alvarez-Monjarás et al., 2019; Parent et al., 2011).

Mindful parenting (MP) primarily emphasizes being fully present and attentive in the parenting role, with a focus on the present moment (Duncan et al., 2009a). MP is a multidimensional construct that bridges intrapersonal and interpersonal aspects of parenting. It extends mindfulness, or “the awareness that emerges through paying attention, on purpose, in the present moment, and nonjudgmentally to the unfolding of experience moment by moment” (Kabat-Zinn, 2003, p. 145)—drawn from Buddhist tradition—into parenting interactions. A commonly used framework for MP incorporates multiple dimensions of listening with full attention, nonjudgmental acceptance, emotional awareness, self-regulation, and compassion for both the child and for oneself as a parent (Duncan et al., 2009a). From this perspective, parents can learn to intentionally practice MP in their day-to-day, moment-to-moment parenting interactions. Intervention research in this area has taken a skills-building approach that involves varying degrees of informal mindfulness practice in daily life (e.g., Coatsworth et al., 2015) and/or training in formal mindfulness meditation (e.g., Potharst et al., 2019).

Parental reflective functioning (PRF), on the other hand, is a psychological construct that describes a parent’s ability to mentalize, or to understand their own and their child’s inner experiences—thoughts, emotions, and desires—and how those mental states drive behavior (Fonagy et al., 1991; Fonagy & Target, 1997; Sharp & Fonagy, 2008; Slade, 2005). It often involves a more in-depth exploration of the child’s inner world and includes a parent’s ability to think about and understand the motivations, emotions, and intentions behind their own and their child’s behavior. In PRF, parents’ reflections on their own and their child’s mental and emotional experiences extend beyond the present moment. More specifically, parents draw connections between past experiences and current mental states. PRF is frequently used in therapeutic settings to promote a deeper understanding of parent-child dynamics and promote sensitive and responsive parenting (e.g., Slade et al., 2020; Suchman et al., 2018).

Shaver et al. (2007) proposed that mentalization, or reflective functioning, overlaps with mindfulness because both processes require cognitive resources to openly observe one’s own and the other’s thoughts, needs, and emotions. These overlapping processes are both associated with parenting as well, such that parents with higher MP and PRF are more likely to engage in responsive caregiving and greater sensitivity (Block-Lerner et al., 2007; Suchman et al., 2018). Despite theoretical and conceptual overlap between MP and PRF, there is a lack of research examining the two concepts concurrently in a single study. In one study, Falkenström et al. (2014) found a positive association between dispositional mindfulness and reflective functioning together. However, these constructs were not assessed in relation to parenting, specifically PRF and MP. Although consistent, but separate, bodies of evidence support MP and PRF as promoting positive parenting behaviors and parent-child relationships, a comprehensive scoping review summarizing the overlapping associations across research outcomes and correlates related to parenting is needed.

“Mindful parenting” was first described by Myla Kabat-Zinn and Jon Kabat-Zinn (1997) in their book, *Everyday Blessings.* Since then, researchers and practitioners have developed an array of interventions for improving parenting or parent-child relationships using mindfulness and MP approaches (e.g., Bögels et al., 2014; Duncan et al., 2009b; Singh et al., 2007). MP involves cultivating awareness of one’s thoughts, feelings, and the child’s needs, promoting a calm and compassionate parenting style through emotion regulation and values-based intention setting (Bögels & Restifo, 2014; Duncan et al., 2009a; Kabat-Zinn & Kabat-Zinn, 1997). MP is associated with a warm and nurturing parent-child relationship, reduced stress, and improved well-being for both parents and children (Chaplin et al., 2018; Gouveia et al., 2016; Parent et al., 2016a, b). MP is thought to address both intrapersonal and interpersonal aspects of parenting through five core aspects: (a) listening with full attention, (b) emotional awareness of self and child, (c) nonjudgmental acceptance of self and child, including greater awareness of expectations and attributions, (d) self-regulation in the parenting relationship, and (e) adopting compassion toward oneself as a parent and the struggles one’s child faces (Duncan et al., 2009a). From this perspective, the foundation of MP involves practicing moment-to-moment awareness of one’s thoughts and emotions and those of the child. MP also involves suspending judgmental attributions through an open and receptive stance and engaging in more intentional parenting behaviors.

MP is also reflected in parents’ ability to be fully present with their children and to bring an attitude of acceptance, kindness, and compassion to those interactions. Interpersonal processes targeted by mindfulness-based interventions include perspective-taking, empathic responding, communication, and anger management (Block-Lerner et al., 2007; Wachs & Cordova, 2007). MP thus can play an important role in promoting positive parent-child relationships. For example, MP is associated with greater use of authoritative (versus authoritarian or permissive) parenting styles (Gouveia et al., 2016), increased positive parenting behaviors, such as warmth and reinforcement (Parent et al., 2016a, b), and more positive emotions and affective behaviors during parent-child interactions (Duncan et al., 2015; Turpyn & Chaplin, 2016).

A different, but related, parenting construct is reflective functioning, or the operationalization of the mental process known as mentalizing. Mentalizing is the ability to understand our own and other’s behaviors in relation to underlying mental states and intentions (Fonagy et al., 1991; Fonagy & Target, 1997). Importantly, reflective functioning includes the ability to reflect on both one’s own and others’ mental states and connect those feelings to behavior, distinguishing it from similar concepts such as empathy or meta-cognition (Suchman et al., 2010). A high capacity for reflective functioning includes an understanding of the complexity of mental states—that they can be contradictory, ambiguous, changing, hidden, or disguised (Slade, 2005). Developmentally, reflective functioning is essential to affect regulation and the development and sustainability of social relationships, especially parent-child relationships. Parental reflective functioning (PRF) has been proposed as one mechanism that contributes to the intergenerational transmission of attachment, via parenting sensitivity (Fonagy et al., 1995; Fonagy & Target, 1997; Slade et al., 2005a, b).

PRF is important to parent-child relationships for several reasons. First, reflective capacity allows the child and the parent to see that behavior is both predictable and meaningful, and to understand each other’s mental states and intentions (Slade, 2005). Second, PRF has been consistently linked to higher parenting sensitivity (e.g., Buttitta et al., 2019; Slade et al., 2005a, b; Suchman et al., 2010, 2018), and secure attachment in children (e.g., Hoffman et al., 2006; Stacks et al., 2014). Third, reflective functioning helps decipher a person’s inner from outer reality. For example, a child can attribute their parent’s insensitive behaviors to the parent’s emotional or mental states, rather than to themselves as an unlovable child. Fourth, reflective functioning promotes effective communication, which is important in parenting and the parent-child relationship. Reflective functioning allows one to be open to others’ thoughts and emotions and that they may differ from oneself. Indeed, reflective functioning has been linked to perspective-taking (Fonagy et al., 2016a, b). Lastly, reflective functioning connects meaning to internal and external experiences. Children depend on their parents to facilitate processes such as affect regulation and self-organization of thoughts, feelings, and mental states. Parents enacting reflective functioning are actively helping the child construct their sense of self and others. In contrast, parents’ failure to accurately mirror or reflect the child’s emotional needs can lead their child to have a distorted representation of the self and others (Slade, 2005). High levels of PRF are associated with improved parent-child attachment, better child emotional regulation, and more sensitive parenting (e.g., Buttitta et al., 2019; Camoirano, 2017; Slade et al., 2005a, b).

Relatedly, it is important to clarify the focus on PRF independent of other elements of mentalization. Although different aspects of mentalization, including insightfulness and mind-mindedness are conceptually similar to PRF, this scoping review will focus only on PRF. As discussed in Camoirano’s (2017) review, these constructs may not tap into the same mental or cognitive capacity as PRF (Van Ijzendoorn & Bakermans-Kranenburg, 2019). PRF is thought to capture the capacity to mentalize, or an overall cognitive ability, while mind-mindedness and insightfulness captures the extent to which parents accept children’s mental states and balance positive and negative aspects of children, as captured by insightfulness (Medrea & Benga, 2021). More research is needed to clearly delineate these constructs (Camoirano, 2017; Medrea & Benga, 2021). However, the mentalization construct most closely aligned with MP is PRF, because both involve reflective mental processes. Given the conceptual similarity, it is important to compare research evidence supporting these two sectors of research and examine their potential overlaps.

Following this framing of the theoretical and empirical backgrounds for MP and PRF, we describe our approach to conducting a scoping review of both literatures. This scoping review is necessary to map the depth and breadth of the existing evidence of MP and PRF in the parenting literature. The results from this review may inform future studies such as systematic reviews, research that compares MP and PRF directly, and the design of parenting programs. Scoping reviews examine the extent, variety, and characteristics of the evidence on a topic and findings from this type of review can help determine if there is value in conducting a systematic review on the same topic (Tricco et al., 2018). Lastly, a scoping review can identify gaps in the existing literature and in turn, present directions for future research. By conducting this study, we aim to cross MP and PRF sub-fields to allow for refinement in our understanding and the potential value of each approach. Our long-term goal is for researchers to consider examining these two constructs together to move our current understanding of their impacts on parenting and parent-child relationships forward. We describe the theoretical background of MP and PRF in the context of parenting in the sections. To begin, we highlight the limitations of existing PRF and MP reviews.

To date, scoping or systematic reviews in this area exist but not without limitations, specifically narrow inclusion criteria (Donovan et al., 2022; Shorey & Ng, 2021; Townshend et al., 2016). For example, the scoping review by Donovan et al. (2022) scoping review excluded qualitative studies and cross-sectional studies, whereas Hidayati and Hartini (2022) only included studies with parents of adolescents. Previous reflective functioning reviews exist, but with important limitations. For example, both Camoirano’s (2017) and Katznelson’s (2014) reviews focused on studies that used certain reflective functioning measures such as the Reflective Functioning Scale (RFS; Fonagy et al., 1998) and/or Adult Attachment Interview (AAI; George et al., 1985), and excluded studies employing the Parental Reflective Functioning Questionnaire (PRFQ; Luyten et al., 2017) or other reflective functioning measures, hence, providing an incomplete scope of the field. Additionally, Katznelson’s (2014) review focused primarily on reflective functioning and psychopathology, with a limited focus on adult and child attachment.

The present study focuses on conducting a scoping review, with wider inclusion criteria, to identify and map the existing evidence for MP and PRF from 2005 to mid-January 2020, summarize the study characteristics across both areas and identify future research questions to explore. Specifically, the rationale for this scoping review is to provide fuller coverage of the body of literature on MP and PRF to address the following questions within the parenting literature: (1) What are the overlapping or distinctive outcomes and correlates across the mindful parenting and parental reflective functioning literature? (2) To what extent should these separate theoretical and empirical lines of research be integrated (or not) in future research? What are the gaps in this area to inform future inquiry?

## Method

**Fig. 1:**
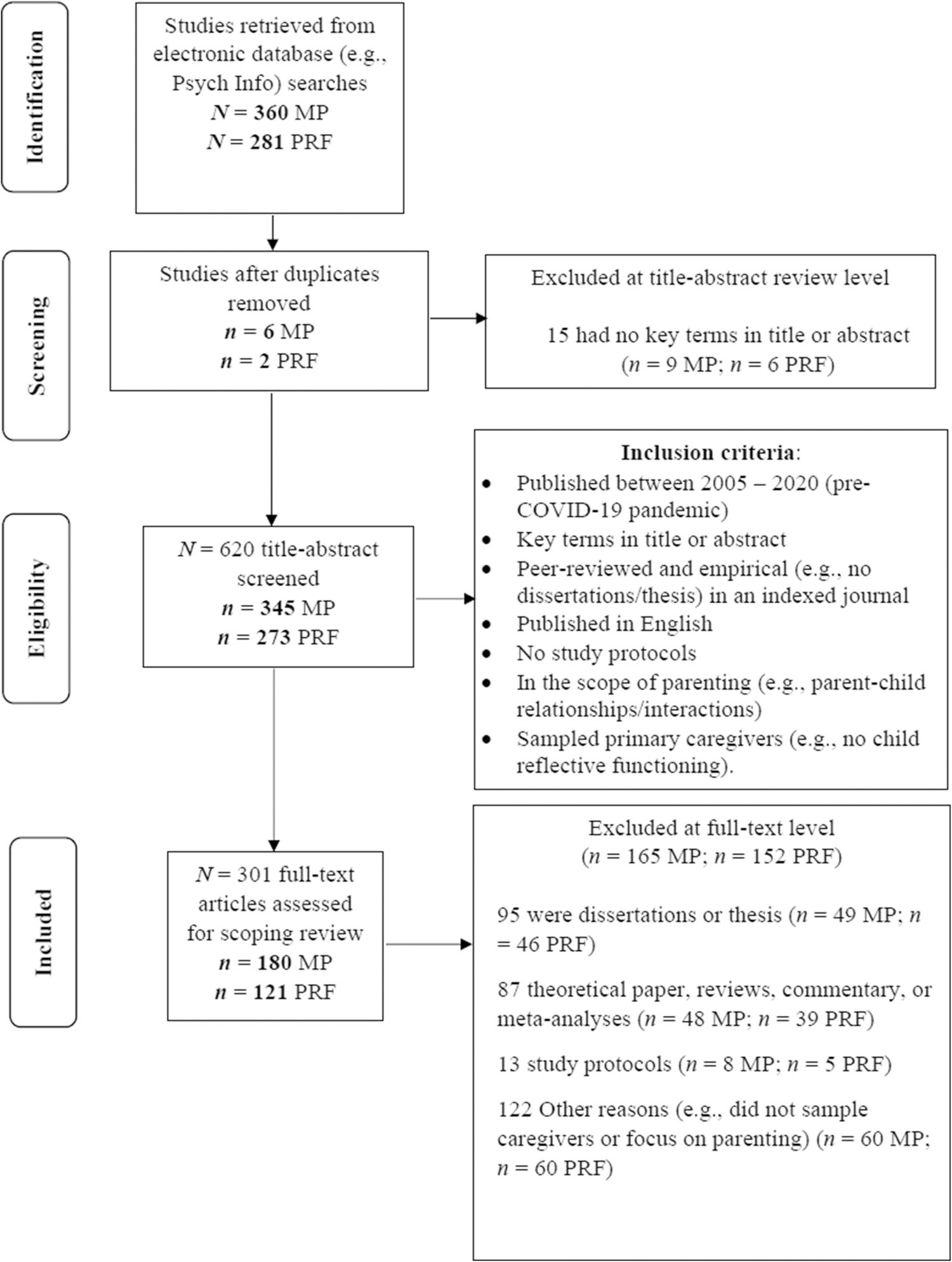
PRISMA flow diagram for the research and inclusion criteria in the review. MP=mindful parenting; PRF=parental reflective functioning

The scoping review was designed and reported following the Preferred Reporting Items for Systematic Reviews and Meta-Analyses-Extension for Scoping Review (PRISMA-ScR; Tricco et al., 2018) criteria, which guided our methods and reporting of the findings. Fig. 1 depicts the PRISMA stages of our scoping review process: identification, screening, eligibility, and inclusion. Detailed procedures for each stage of the process are explained below.

### Identification

The first step was to obtain consensus on the years to cover in our search (2005-Mid- January 2020), search engines and databases (Google Scholars, PubMed, PsychInfo, EBSCOHost, and MINDRxiv), and search terms to use when conducting the MP and PRF literature searches. Our search strategy included the following search terms, “parenting” “parent-child relationships” “parent-child dyads” with a combination of the search terms “parental reflective functioning,” “parental mentalization”, “paternal/maternal reflective functioning,” “paternal/maternal mentalization.” For the MP literature, we used the search terms, “mindful parenting,” “mindfulness,” and “parenting.” The fifth author (VC) conducted the first PRF and MP literature searches. Then, the first author (TH for PRF studies) and the third author (CK for MP studies) conducted additional literature searches. For the last step of the identification phase, we determined the key data to extract from each article during the full-text assessment such as sample size and demographic information.

### Screening

Both TH (PRF studies) and CK (MP studies) independently screened the articles at the title-abstract level to remove duplicates before the full-text assessments of the articles, with guidance from the eligibility criteria for this scoping review.

### Eligibility Criteria

To expand beyond past reviews (Camoirano, 2017; Katznelson, 2014), we did not restrict our eligibility criteria to studies using certain measurements of MP or PRF or specific dependent variables (e.g., child attachment or psychopathology). Instead, studies were included in the full-text assessments if they met the following criteria: (1) peer-reviewed (e.g., no dissertations/thesis) in an indexed journal; (2) published in English and between 2005-mid-January 2020; (3) key terms (see above) were included in the title or abstract; (4) empirical articles (e.g., no book chapters); (5) no study protocols; (6) in the scope of parenting (e.g., parent-child relationships; parenting behaviors); and (7) sampled primary caregivers (e.g., not child reflective functioning).

### Inclusion

**Fig. 2:**
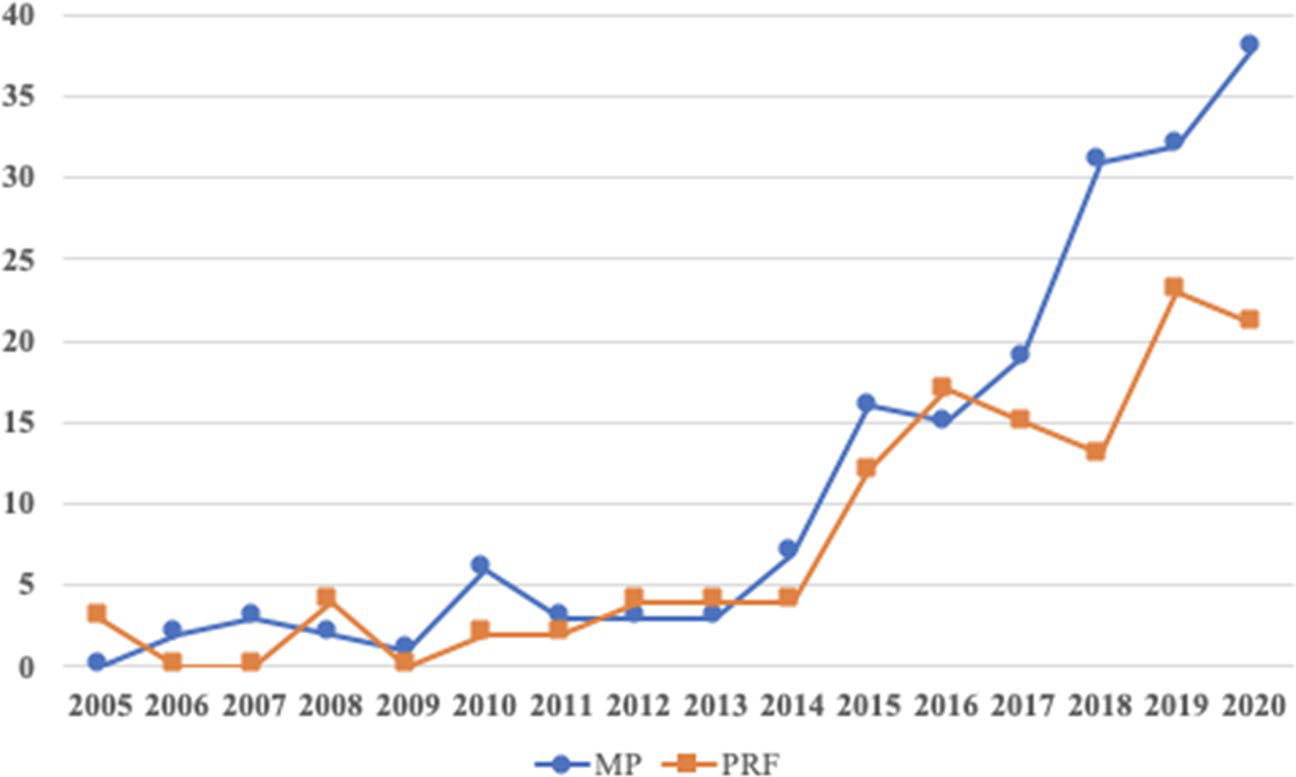
Trends of Mindful Parenting and Parental Reflective Functioning Studies from 2005 through early 2020 (preCOVID-19 pandemic)

All studies that met our eligibility criteria were assessed at the full-text level by study authors. CK, MLK, and EF reviewed the MP literature while TH and MLK reviewed the PRF literature (Fig. 1 and 2).

## Results

Tables 1 and 2 present the key extracted data from the total articles included in this review. As mentioned in the stages used to identify and choose the articles for this review, we identified a total of 641 potential studies (*n* = 360 MP; *n* = 281 PRF) and removed duplicate articles (*n* = 6 MP, *n* = 2 PRF). Next, we ended up with 618 articles after the title-abstract screening (*n* = 345 MP; *n* = 273 PRF). After the second screen process, we eliminated 317 articles that did not fit our eligibility criteria (*n* = 165 MP; *n* = 152 PRF) (see Fig. 1). After analyzing the data extracted from the included studies (*N* = 301; *n* = 180 MP, *n* = 121 PRF), we identified the key characteristics, commonalities, and differences across the articles, which are described below and presented in Tables 1 and 2.

### Characteristics of Included Studies

#### Timeline (Dates) for Research

Figure 2 depicts the patterns of empirical studies on MP and PRF published from 2005–2020. Although zero MP articles were found in 2005 (the year Dumas highlighted the value of a MP approach: Dumas, 2005), MP articles were steadily published from 2007 to 2014. Starting from 2015, there was a significant increase in MP empirical studies in 2015 (*n* = 16), 2016 (*n* = 15), 2017 (*n* = 19), 2018 (*n* = 31), 2019 (*n* = 32), and 2020 (*n* = 38).

For PRF, only three articles were published in 2005 and zero PRF articles were included in our scoping review for the years 2006, 2007, and 2009. In 2008, 2012, 2013, and 2014, 16 studies were published (4 each year) and two articles were published each in 2010 and 2011. However, after 2015, there was a substantial increase in PRF empirical articles: 2015 (*n* = 12), 2016 (*n* = 17), 2017 (*n* = 13), 2018 (*n* = 12), 2019 (*n* = 23), and 2020 (*n* = 21).

#### Country

The majority of the studies derived from the United States (*n* = 65; 36.1% for MP; *n* = 48; 40% for PRF). The remaining MP literature was from the Netherlands (*n* = 20; 11.1%), Portugal (*n* = 16; 8.9%), Canada and China (*n* = 10 per country; 5.6% each), the United Kingdom (*n* = 16; 8.9%), Australia (*n* = 6; 3.3%), Hong Kong (*n* = 5; 2.8%), Iran, (*n* = 4; 2.2%), Belgium, Chile, Croatia, Egypt, Jordan, Ireland, and Sweden (*n* = 2 per country; 1.1% each), and India, Israel, Spain, Taiwan, Turkey, and Vietnam (*n* = 1 per country; 0.6% each). Six studies did not report the country (3.3% total), and two studies included 36 countries (1.1% total).

The remaining 60% of the PRF studies were from Canada (*n* = 12; 10%), the United Kingdom (*n* = 9; 7%), Australia (*n* = 7; 6%), Italy (*n* = 7; 6%), the Netherlands (*n* = 5; 4%), Finland, Spain, Chile, and Norway (*n* = 4 per country; 3% each), Denmark (*n* = 3; 2%) and Switzerland, (*n* = 2; 3%), and Germany and Turkey (*n* = 2; 2%). The remaining PRF studies were from the Caribbean, Poland, Sweden, and Belgium (*n* =1 per country/region; 1% each), except one study for which the country was not reported (e.g., Væver et al., 2020).

#### Intervention

Results from this scoping review indicated that 57% (*n* = 104) of MP studies implemented a related intervention. Of the 103 studies, the most commonly implemented interventions (*n* = 23; 22%) were Mindfulness-Based Stress Reduction (MBSR; e.g., Corthorn, 2018; Gannon et al., 2017; Neece, 2014), Mindfulness-Based Cognitive Therapy (MBCT; *n* = 12; 12%, e.g., Evans et al., 2019; Ferraioli & Harris, 2013; Gurney-Smith et al., 2017), and 17 studies (16%) employed elements of both MBSR and MBCP (Bögels et al., 2014; Mah et al., 2020; Zhang et al., 2017). In addition, Mindfulness-Based Childbirth and Parenting (MBCP; *n* = 9; 9%, e.g., Duncan & Bardacke, 2010; Price et al., 2019; Warriner et al., 2018), Mindfulness-Enhanced Strengthening Families Program (MSFP; *n* = 7; 7%, e.g., Coatsworth et al., 2018; Lippold et al., 2019), Mindfulness-Based Positive Behavior Support (MBPBS; *n* = 3; 3%; Singh et al., 2014) and MyMind (*n* = 2; 2%; Ridderinkhof et al., 2018) were studied. About 9% (*n* = 9) of studies adapted their interventions from more than three interventions listed above. The remaining studies either did not provide information about the source of the mindfulness-based intervention that was employed (*n* = 17; 16%) or used sources not identified in this review (*n* = 5; 5%). While most of the interventions were delivered in person, online interventions (Boekhorst et al., 2020; Shaffer et al., 2020) and app-based interventions (Hunter et al., 2019; Yang et al., 2019) were introduced in 2019 and 2020.

Over one-quarter, or 27% (*n* = 33), of the PRF studies implemented a mentalization-related intervention. The most implemented interventions were Minding the Baby (15%; *n* = 5; e.g., Slade et al., 2020) and Circle of Security—Parenting (12%, *n* = 4; e.g., Huber et al., 2015). The remaining studies employed the Mothers and Toddlers/Mothering from the Inside Out (18%, *n* = 6; e.g., Suchman et al., 2012, 2016), Family Minds (6%, *n* = 2; e.g., Bammens et al., 2015), and 16 studies (48%; e.g., Byrne et al., 2019; Zimmer-Gembeck et al., 2019) used different programs such the Bright Program (Paris et al., 2015), Nurturing Attachments Program (Staines et al., 2019), Reflective Fostering Program (RFP; Midgley et al., 2019), or Baby Court (Stacks et al., 2019). Within these 16 studies, one study (Enav et al., 2019) mentioned using a mentalizing-based intervention for parents of children diagnosed with autism spectrum disorder (ASD), however, the researchers did not report the name of the program.

#### Population Targeted

In the MP literature, 46% (*n* = 83) sampled mothers only, 47% (*n* = 85) both mothers and fathers with mothers being the majority, 2% (*n* = 4) with equal proportions of mothers and fathers, and 1% (*n* = 2) sampled parents but did not state the gender identity or gender role of the parents. Only one study sampled fathers only (MacDonald & Hastings, 2010) and 3% (*n* = 5) included caregivers/teachers/cohabiting partners along with parents. Of the total 180 studies, 8% (*n* = 14) studies sampled parents who were considered at high risk for poor outcomes. Seven studies (4%) recruited parents with mental health challenges including depression (Evans et al., 2019; Mann et al., 2016; Parent et al., 2011), anxiety (Goodman et al., 2014), stress (Turpyn et al., 2019), and difficulties with emotion regulation (Wilson & Donachie, 2018). The other seven studies (3.9%) recruited mothers with opioid and substance use disorder (Gannon et al., 2017; Short et al., 2017), methadone maintenance (Dawe & Harnett, 2007), FMR1 premutation (Hunter et al., 2019), preterm premature rupture of membranes (Korukcu & Kukulu, 2017), history of sexual trauma (Price et al., 2019), and parents who had been exposed to a high rate of violence (Hicks et al., 2018). In addition, 39% (*n* = 71) studies sampled parents of children who were at risk for intellectual and developmental disabilities, mental health problems, or internalizing and externalizing problems.

In the PRF literature, 26% (*n* = 32) sampled mothers, 2% (*n* = 3) fathers, 2% (*n* = 3) pregnant women, 2% (*n* = 3) couples, 2% (*n* = 3) parents (no specification), 11% (*n* = 13) parent or caregiver-child dyads, 39% (*n* = 47) mother-child and 1% (*n* = 1) father-child dyads. Of the studies that sampled mothers, four studies focused on mothers with substance-related abuse or treatment (e.g., Suchman et al., 2011), with four studies specifically on mothers with substance abuse disorder (Håkansson et al., 2018). The remaining articles sampled young mothers (Sadler et al., 2013), mothers from outpatient mental health clinics (Suchman et al., 2016), and mothers with postpartum depression (Cordes et al., 2017). Two of three studies that sampled fathers specifically targeted fathers who had committed intimate partner violence (IPV; Mohaupt & Duckert, 2016; Stover & Coates, 2016). Of the remaining articles, 4% (*n* = 5) sampled foster or adoptive parents (Bammens et al., 2015; Bunday et al., 2015; León et al., 2015), and one study (Zimmer-Gembeck et al., 2019) included mothers, fathers, and foster parents. Additionally, 2% (*n* = 3) did not report the gender identity or gender role of the parents (Ashton et al., 2016; Ensink et al., 2017b; Staines et al., 2019). Lastly, León and Olhaberry (2020) included a triad sample of mothers, fathers, and children while Mata López, Álvarez, and Gómez (2020) included parents, children, and teachers.

Conclusively, 38.2% (*n* = 115) of the included articles (*N* = 301) targeted mothers only, and only 2.3% (*n* = 7) focused exclusively on fathers. Across the MP and PRF studies, an overlap was identified with both areas of research sampling parents with specific, targeted characteristics including parents considered “at risk,” and those with psychopathology (e.g., depression), or substance use disorder.

#### Race/Ethnicity of Samples

Of the total 180 MP studies, 81 articles did not report the race or ethnicity of their sample. Across the remaining 99 studies, that did explicitly report their race or ethnicity of their sample, 41 studies had predominantly White (or self-identified as “Caucasian”) samples. Across all 99 studies, the average percentage of participants that identified as White or “Caucasian,” in each study was 71% (range: 0–100), 12% (range: 0–100) for Black/African American, 7.65% (range: 0–47.5) for Hispanic/ Latiné, 4.13% (range: 0–17) for Asian/Asian American, 0.50% (range: 0–6) for Native American/Hawaiian/Alaskan Native/Pacific Islander, 0.48% (range: 0–63) for Multiracial, and 3.75% (range: 0–21) for “Other.”

For PRF, 30 studies out of 121 total PRF studies did not report the race or ethnicity of their sample. Across the 91 studies that explicitly reported the race/ethnicity of their sample, 49 had predominantly White or self-identified “Caucasian” samples. More specifically, across all 93 studies the average percentage of participants who identified as White or “Caucasian,” was 60% (range: 0–100), 18.36% (range: 0–75) for Black/African American, 19.45% (range: 0–88) for Hispanic/Latiné, 1.75% (range: 0–22) for Asian/Asian American, 0.33% (range: 0–2) for Native American/Hawaiian/Alaskan Native/Pacific Islander, 2.24% (range: 0–18) for Multiracial, and 3.73% reported (range: 0–14) “Other.”

#### Measures Employed

In the MP articles, 40% (*n* = 72) MP articles used the original 10-item Interpersonal Mindfulness in Parenting (IM-P) short-form (Duncan, 2007) or the expanded 31-item IMP version (Duncan, 2023), while 26.6% (*n* = 48) of studies used the Five Facet Mindfulness Questionnaire (FFMQ; Benn et al., 2012) (*n* = 48; 26.6%). These MP measures were most commonly used to assess MP specifically, and in general, mindfulness among parents in the MP literature. Another frequently used measure was the Mindfulness Attention Awareness Scale (MAAS; *n* = 30; 16.6%). Since 2014, a wider array of measures was used, including the Bangor Mindful Parenting Scale (BMPS; Jones et al., 2014) (*n* = 8; 4.4%), the Mindfulness in Parenting Questionnaire (MIPQ; Seidman et al., 2019) (*n* = 4; 2.2%), the Freiburg Mindfulness Inventory (FMI; Walach et al., 2006) (*n* = 3; 1.7%), and the Cognitive and Affective Mindfulness Scale-Revised (CAMS-R; Feldman et al., 2007; *n* = 2; 1.1%). Some measures were developed and only included in publications a handful of times. The Subjective Units of Use of Mindfulness (SUUM; Singh et al., 2006) (*n* = 2) and the Toronto Mindfulness Scale (TMS; Lau et al., 2006) (*n* = 1) were used only in 2006 and 2007 and were not used in future studies in this review. In 2019, one study (Benton et al., 2019) employed an adapted version of the Mindful Parenting Observation Scale (MPOS; Geier, 2012) which assesses the observed behavior of MP. In addition, 23 studies (12.7 utilized more than one measure. Particularly, 17 (9.4%) studies used the IM-P in tandem with the FFMQ (*n* = 10), the MAAS (*n* = 6), and FMI (*n* = 1). Moreover, of the total 180 MP studies, 33 studies (18.3%) did not employ any specific MP measures.

The most frequently used measures of PRF are the Parent Developmental Interview (PDI; Aber et al., 1985) (*n* = 41; 33.8%), the Parental Reflective Functioning Questionnaire (PRFQ; Luyten et al., 2017) (*n* = 26; 21.4%), the Adult Attachment Interview (AAI; George et al., 1985) (*n* = 17; 14%), Pregnancy Interview (PI-PDI; Smaling et al., 2015) (*n* = 10; 8.3%), PDI-Revised (PDI-R; Slade et al., 2004) (*n* = 7; 5.8%), Reflective Functioning Questionnaire (RFQ; Fonagy et al., 2016a, b) (*n* = 5; 4.1%), the PDI-Short Form (Stacks et al., 2014) (*n* = 4; 3.3%), Reflective Functioning Scale (RFS; Fonagy et al., 1998) (*n* = 2, 1.7%), or PDI-Modified (Steele et al., 2007) (*n* = 1; .8%). Notably, the Working Model of the Child Interview (WMCI; Zeanah et al., 1996) was used to assess PRF in 2005 (*n* = 1) and 2008 (*n* = 2), but not again until 2019 (*n* = 1) and 2020 (*n* = 2). More recently, new PRF measures were developed to assess PRF such as the Parental Embodied Mentalizing (PEM; Shai et al., 2017), the Limit Setting Interview (Möller et al., 2017), and the Mini-Parent Reflective Functioning Interview (Mini-PRFI; Ensink et al., 2019). Additionally, 13.2% (*n* = 16) of PRF studies employed more than one measure of PRF. For example, Hertzmann et al. (2016) used both the PDI and the PRFQ whereas 31.3% (*n* = 5) of those 16 studies used both the PDI and the PI-PDI in their study (Ordway et al., 2014; Pajulo et al., 2008, 2012; Sadler et al., 2013; Smaling et al., 2016). Möller et al. (2017) was the only study that employed three PRF measures (PDI, Limit Setting Interview, and RFQ). Lastly, the remaining PRF studies employed a different instrument to assess PRF such as the Rumination Reflection Questionnaire (e.g., Waldman-Levi et al., 2020), or Prenatal PRFQ (e.g., Røhder et al., 2020).

#### Independent Variables

More than half of the MP studies (*n* = 103; 57.2%) implemented mindfulness-based interventions and focused on pre- and post-assessments of the intervention. Studies with interventions mostly measured changes in parents’ levels of MP across time and groups (e.g., Chaplin et al., 2018; Potharst et al., 2019). Other common independent variables that were assessed in MP studies included dispositional mindfulness (e.g., Gouveia et al., 2016; Hicks & Dayton, 2019; Parent et al., 2016a, b; Zhang, Wang, & Ying, 2019b), anxiety (e.g., Henrichs et al., 2019), parenting stress (e.g., Chan & Neece, 2018; Laurent et al., 2017), parent-child related problems (e.g., Chan & Lam, 2017; Whitlock et al., 2018), and parent attachment (e.g., Moreira et al., 2016).

As noted earlier, 33 studies implemented a mentalization-based intervention. Not surprisingly, *n* = 33 (27.3%) focused on pre- and post-assessments of the program with a particular emphasis on changes in PRF scores (e.g., Sadler et al., 2013; Suchman et al., 2008). Other independent variables that were frequently examined in the remaining PRF studies (*n* = 90; 74.3%) varied, such as intimate partner violence (Mohaupt & Duckert, 2016), maternal and child attachment (Slade et al., 2005a, b), parenting reflectivity (Rosenblum et al., 2008), maternal accuracy (Ha et al., 2011), parenting sensitivity (Borelli et al., 2012), parenting behaviors (Ensink et al., 2017b), treatment fidelity of the mentalizing program (Suchman et al., 2012), eating disorder symptoms (Claydon et al., 2016), child sexual abuse (Ensink et al., 2016), and trauma and attachment (Cristobal et al., 2017).

#### Dependent Variables

Of the 180 MP studies, 47.2% (*n* = 85) of studies examined parent outcomes only, 18.8% (*n* = 34) focused on child outcomes only, and 33.3% (*n* = 60) included both parent and child outcomes. There was one study (Fernandes et al., 2020a) that did not examine either parent or child outcomes. This study assessed the usefulness of a MP intervention. The most commonly included parenting outcomes were: MP and mindfulness (*n* = 63; 74.1%; e.g., Lunsky et al., 2015; Potharst et al., 2018b; Rice et al., 2020), psychological distress including parenting stress (*n* = 71; 83.5%; e.g., Corthorn, 2018; Lo et al., 2017), depression (*n* = 27; 31.7%; e.g., Duncan et al., 2017; Pan, Gau, et al., 2019a; Pan, Chang, et al., 2019b), and anxiety (*n* = 18; 21.2%; e.g., Geurtzen et al., 2015; Rayan & Ahmad, 2017). For child outcome variables, child behavior challenges (*n* = 23; 67.6%; e.g., Beer et al., 2013; Srivastava et al., 2011) and internalizing and externalizing problems (*n* = 14; 41.2%; Haydicky et al., 2015; Parent et al., 2016a, b) were most commonly assessed.

From the 121 PRF studies, PRF was the dependent variable for half (47.9%; *n* = 58) of the articles, with 0.8% (*n* = 1) study focused on *prenatal* reflective functioning (Smaling et al., 2015) and 0.8% (*n* = 1) on postpartum reflective functioning (Rutherford et al., 2018). The remaining studies (*n* = 64) focused on a range of outcome variables—either in addition to PRF or separately—such as mothers’ representation of the children (Schechter et al., 2005), atypical maternal behavior (Schechter et al., 2008), child conduct problems (Ha et al., 2011), adolescent reflective functioning and behaviors (Benbassat & Priel, 2012), child anxiety (Esbjørn et al., 2013), maternal distress tolerance (Rutherford et al., 2013), infant attachment disorganization (Berthelot et al., 2015), parenting stress (Adkins et al., 2018), and child temperament (Vismara et al., 2020).

#### Overlaps in Correlates and Outcomes

Review of studies for both MP and PRF revealed overlap in four general constructs: (a) parent well-being; (b) child well-being; (c) parenting behaviors; and (d) attachment. Specifically, 58.3% of MP studies (*n* = 105) and 28.9% of the PRF studies (*n* = 35) included a variable related to parents’ well-being (e.g., parenting stress, anxiety, depression; e.g., Hertzman et al., 2016; Kohlhoff et al., 2016; Lo et al., 2017; Short et al., 2017; Wheeler et al., 2018). Further, 41.6% of MP (*n* = 75) and 23.1% PRF studies (*n* = 28) examined child well-being (e.g., internalizing and externalizing behaviors; e.g., Parent et al., 2016a, b; Mann et al., 2016; León et a., 2015) and 38.3% of MP (*n* = 69) and 38.8% PRF studies (*n* = 47) evaluated parenting behaviors (e.g., responsivity, sensitivity; e.g., Weitlauf et al., 2020, Rutherford et al., 2013; Borelli et al., 2012). Lastly, 1.6% of MP (*n* = 3) and 21.5% PRF studies (*n* = 26) focused on some attachment-related variables, such as parent attachment (e.g., Cristobal et al., 2017; Korukcu & Kukulu, 2017), child attachment (e.g., Ensink et al., 2019; Moreira et al., 2018), or attachment anxiety (e.g., Moreira et al., 2016; Nijssens et al., 2018).

## Discussion

This scoping review covers empirical evidence from 2005 to mid-January 2020 linking MP and PRF with other positive parenting outcomes. We present conclusions regarding the research questions that guided this scoping review, discuss the conceptual and theoretical overlaps of MP and PRF based on the results of our study, and suggest future directions for these lines of research.

The results of the scoping review demonstrated a significant association between MP and other elements of positive parenting and parent and child well-being, including parental warmth and responsiveness (Campbell et al., 2017; Duncan et al., 2015), less parenting stress (Zeegers et al., 2019), and fewer child externalizing and internalizing problems (Han et al., 2019). About two-thirds of the studies included a mindfulness-based intervention aimed at increasing levels of mindfulness in parenting. Most of the interventions were based on MBSR and/or MBCT, which aimed to improve parents’ ability to cope more effectively and reduce psychological reactivity to stressful parenting situations by bringing mindful awareness to moment-to-moment parent-child interactions while being nonjudgmental of self and child. Participation in mindfulness-based interventions was found to significantly reduce various types of stress including stress related to parenting (Potharst et al., 2019; Zeegers et al., 2019), perceived stress (Seidman et al., 2019), and general stress (Townshend et al., 2018). Other notable advantages of MP were reported in parents’ psychological functioning, such as a reduction in overreactive parenting (Potharst et al., 2019) and reduced emotion dysregulation (Gershy et al., 2017; Lengua et al., 2018). Applying mindfulness in parenting can alter not only parents’ intrapersonal experiences as a parent but also interpersonal experiences between parent and child. Researchers reported an association between parental mindfulness with a more optimal parenting style (e.g., authoritative parenting, parental warmth; Williams & Wahler, 2010; Duncan et al., 2015), positivity (Jones et al., 2014), and less negative emotion expression toward the child (Turpyn & Chaplin, 2016).

Additionally, the effects of MP on parents’ psychological well-being and positive parenting outcomes were found promising for parents considered to be “at risk.” For example, scholars have targeted interventions for parents with depression (e.g., Mann et al., 2016), substance use disorder (e.g., Short et al., 2017), and mood, anxiety, and stress disorder (e.g., Zeegers et al., 2019). After participation in mindfulness-based interventions that focused on cultivating mindfulness in the parenting context, these parents reported improvements in both their clinical symptoms (e.g., reduction in depression and anxiety symptoms) and parenting-related outcomes (e.g., reduction in parenting stress and increase in acceptance toward child). Notably, these studies did not examine mechanistic changes in levels of mindfulness or MP in parents after the intervention. However, several cross-sectional studies found evidence that levels of dispositional mindfulness are significantly related to parents’ psychological well-being considered to be at “high risk” (Hicks et al., 2018; Parent et al., 2011). Through MP interventions, parents may improve their ability to manage negative emotions and stress that arise in their parenting role.

A stream of research that has examined the efficacy of mindfulness-based interventions for pregnant women at risk of perinatal depression or anxiety also found similar results (Goodman et al., 2014; Korukcu & Kukulu, 2017; Townshend et al., 2018). These studies demonstrated that developing mindfulness skills and applying them to parenting helped expectant mothers cope with anxiety and depression related to pregnancy, birth, and early parenthood, and significantly increased mindfulness and self-compassion. Perinatal mindfulness intervention studies with lower risk samples also indicate they may prevent postpartum depression symptoms (Duncan et al., 2017). Researchers have often focused on targeted samples of parents suffering from or at risk for depression, anxiety, and other psychological disorders. However, a new stream of research has developed in examining more diverse groups such as parents with obesity (Jastreboff et al., 2018) and military-deployed parents (Zhang et al., 2018; Zhang, Zhang, & Gewirtz, 2019a).

Evidence from the studies in this review demonstrates that PRF has significant implications for parenting and child development. For example, PRF is associated with parenting sensitivity (Ensink et al., 2016), parenting stress (Nijssens et al., 2018), child attachment (Slade et al., 2005a, b), and child behavior problems (Suardi et al., 2020). One-quarter of the studies implemented mentalization-based interventions that sought to enhance parents’ capacities for reflective functioning. These programs help caregivers, via PRF, fully understand their view of their child, themselves, and their parenting. Specifically, reflective functioning enables parents to consider how their past (their own childhood memories) and present (perceptions of the child, current mental states) influence their caregiving behaviors, their child’s behavior, and the parent-child relationship. Parents’ failure to engage in high levels of PRF can result in less sensitive parenting and child attachment insecurity (Ensink et al., 2019). The intervention studies included in this review provided evidence that mentalization-based interventions improve parents’ capacity for reflective functioning, parenting sensitivity, and child attachment (e.g., Slade et al., 2020; Suchman et al., 2018).

Articles from our review of PRF also focused on parent psychopathology. For instance, scholars have targeted interventions toward mothers with substance abuse disorders (e.g., Suchman et al., 2008) and mothers from outpatient mental health clinics (e.g., Suchman et al., 2016). These studies have important clinical implications. Specifically, psychopathology can inhibit parents’ capacity to reflect on both their own and the child’s mental states, potentially resulting in negative child outcomes such as insecure attachment or psychopathology. Taken together, an increase in PRF through participation in mentalization-based programs (e.g., Mothering Inside Out) improves parents’ abilities to provide sensitive care to their children. In specific, parents can help their children identify and organize their mental states about external experiences when parents can recognize how their own feelings impact their behavior. These empirical results have significant clinical implications as it may be critical to target parents who are more vulnerable or susceptible to psychopathology and impaired mentalization.

Moving beyond interventions focused on enhancing PRF, there is also evidence that PRF may mitigate the association between parental stress and psychopathology and risks to healthy child development. In the past, PRF had been primarily tested as a mechanism explaining parenting sensitivity and behavior (e.g., Alvarez-Monjarás et al., 2019; Suchman et al., 2008). Theoretically, however, it should have buffering effects, especially on the association between parenting stress and indicators of parenting behavior or child well-being (e.g., attachment). First, several studies document an inverse association between PRF and parenting stress (León et al., 2015) and a significant reduction in parenting stress after participation in mentalization programs (Huber et al., 2016). Further, even when parents with higher RF are under stress, they may be able to respond with greater sensitivity to their child’s cues, which in turn, promotes child well-being and secure attachment. This theory is supported by several studies in our review, which demonstrated that PRF moderated associations between various risk factors (e.g., SES, stress) and parenting behavior or child outcomes (e.g., Benbassat & Priel, 2012; Borelli et al., 2020a; Buttitta et al., 2019). In sum, the expansive literature on PRF over the last 15 years suggests that it is an important factor in promoting healthy child development and parent-child relationships.

Our review identified several areas of overlap in studies on MP and PRF. Both MP and PRF consist of intrapersonal processes that impact interpersonal relationships between parent and child. Fostering greater MP and PRF in parenting can result in improved parent-child interactions. Studies of MP and PRF demonstrate a range of similar effects, including improvements in parent well-being (e.g., parenting stress, depression), child well-being (e.g., internalizing or externalizing behaviors), parenting behaviors (e.g., responsiveness), and other qualities of the parent-child relationship. Conceptually, both MP and PRF are reflective processes in parenting that may be important targets for understanding how a parent’s intra- and interpersonal relationship with childrearing may contribute to their parenting behaviors and impact on child well-being. Specifically, a commonality between MP and PRF is that both involve cognitive and affective processes in which the parent needs to engage in awareness of their feelings and thoughts to support their child’s emotional needs and thoughts behind their behaviors. To an extent, both MP and PRF entail some level of emotion coregulation and intentional awareness to establish a deeper understanding of the parent’s and child’s internal world (thoughts, feelings, mental states) within the parent-child relationship.

There are some characteristics that differentiate MP and PRF from one another. For example, PRF involves the parents’ capacity to reflect and establish insightful conclusions when discussing the caregiving they received in childhood and its impact on them and their caregiving. Thus, PRF involves deep reflections of the past, which assessment tools such as AAI and PDI aim to facilitate. In contrast, the construct of MP—rooted in mindfulness tenets—focuses on caregivers’ capacity to intentionally bring their attention and awareness to the present moment, allowing thoughts and difficult emotions to arise without judgment, providing compassion to self and the child, especially when the parent or the child are having a difficult interaction. One possible explanation for this difference is that MP is typically captured via self-report assessments while the majority of PRF studies use coded interviews. Evidenced in the attachment literature (Roisman et al., 2007), these different modalities may capture different facets of an overlapping construct. For instance, assessing PRF via coded interviews may capture underlying or more implicit concepts that parents lack enough awareness of to identify in a self-report measure, whereas MP assessed through self-report may capture parents’ more intentional efforts to bring awareness to a given moment in parenting. Future research may benefit from comparing these constructs using the same modality to reveal more about how measurement has impacted their conceptualization. Collectively, given the apparent benefits of MP and PRF interventions, they seem both worthy intervention targets, as they can change and improve through support and training, potentially with synergistic effects.

Findings from this scoping review clarify the need for future research incorporating MP and PRF to advance our current knowledge of reflective processes in parenting and the parent-child relationship. For example, much is unknown regarding the extent to which MP and PRF are correlated with each other and the direction of those associations. Notably, it is unclear whether PRF enhances MP, or vice versa, as rigorous, longitudinal, joint assessments of these constructs and their mechanisms of change do not exist. While there are inconsistencies in how MP has been assessed across the reviewed studies, the *Interpersonal Mindfulness in Parenting scale* (IM-P; Duncan, 2007; Duncan, 2023) is the frequently used measure that explicitly assesses mindfulness in parenting, followed by the *Mindfulness in Parenting Questionnaire* (MIPQ; McCaffrey et al., 2017), which assesses MP of parents with children two-years-old or older. Because there are multiple robust instruments to measure PRF, an unanswered research question that warrants further investigation is how the IM-P relates to various PRF measures (e.g., AAI, PDI, and PRFQ).

Studies assessing attachment, MP, and PRF in a single research design are needed, given that both MP and parenting reflective functioning are correlated with parent and child attachment. Because PRF is grounded in attachment theory, there is a substantial body of research supporting the link between reflective functioning and attachment-related constructs. Although correlational studies have examined the association between MP and both parent attachment (Moreira et al., 2016) and adolescent attachment (Moreira et al., 2018), this research is limited. For example, existing studies utilize a variety of self-report attachment measures, but no studies have compared MP and attachment using “gold-standard” assessments of attachment security (e.g., Strange Situation, AAI).

Collectively examining attachment, PRF, and MP can further our understanding of how these constructs simultaneously influence child development. Specifically, new evidence can advance our understanding of parents’ mental representations of attachment influence MP, and the impact of MP on observed parenting sensitivity and child-attachment security, particularly in infancy and young childhood. There is ample evidence that increases in PRF, through interventions, can facilitate child attachment security (Huber et al., 2015) and parental sensitivity to a child’s cues (Suchman et al., 2008). Still, this evidence is limited to smaller and/or qualitative studies for MP. Moreover, it is still unclear what role MP plays in the association between attachment and PRF. For example, Cristobal et al. (2017) found that maternal insecure attachment was associated with lower PRF. It would be particularly interesting to examine whether MP significantly moderates or mediates the association between parental attachment and PRF as the specific effects of MP on this association are unknown. Given the associations between MP and stress, and that parental reflective capacities tend to be compromised under extreme stress or trauma (Fonagy & Target, 1997), one possibility is that MP buffers the effects of stress on PRF and parenting sensitivity.

One advantage of integrating these two lines of work is identifying unique strengths in each area that may benefit the other. For instance, while emotion regulation has been shown to improve MP interventions (Gershy et al., 2017; May et al., 2016; Wilson & Donachie, 2018), it has not been examined as an outcome of mentalization-based interventions, even though PRF should theoretically improve parents’ ability to regulate their emotions. A few studies have found positive associations between PRF and both parents’ distress tolerance (Rutherford et al., 2013; Rutherford et al., 2015), and emotion regulation skills (Schultheis et al., 2019), but this work is limited to self-report assessments of PRF. Campora et al. (2019) use the AAI to assess maternal reflective functioning but found no significant association with emotion regulation, thus this is an area for additional inquiry.

Parent well-being is important to consider in these studies, given the links between parental well-being and parenting behavior (Dix, 1991). Moreover, parent psychopathology and extreme stress can hinder both parent well-being and the capacity to engage in MP and PRF. There is some evidence that MP buffers the negative effects of life events on mother and infant cortisol levels (Laurent et al., 2017). However, research needs to be expanded to other developmental stages. Researchers have documented a significant improvement in parent well-being via reduction of stress (e.g., Fonagy et al., 2016a, b; Kohlhoff et al., 2016) after participation in a mentalization-based program. In contrast, others found a decrease in parenting stress at post-assessments but no significant effects of the intervention (Hertzman et al., 2016). However, including variables that assess parental well-being is limited and could benefit from additional investigation. Existing studies typically include measures of parenting stress or mental health (e.g., anxiety, depression). However, much is still unknown about how PRF and MP are associated with or impact other facets of parental well-being such as emotional experiences.

Another benefit of looking at these two lines of research together is that it illuminates gaps in targeted developmental stages. For example, there has been an examination of PRF during the prenatal period, with the development of the Pregnancy Interview (Slade et al., 2007), which was administered in 8.2% of the studies reviewed. On balance, MP has been more extensively studied in parents of adolescents (29.1%), whereas only a few studies have explored PRF in parents of adolescents. Additionally, it is necessary to note there are limitations to when MP can be evaluated. For instance, MP is specifically about the parent-child interaction, precluding examining MP before birth. It may only be possible to examine dispositional mindfulness prenatally for first-time parents, and then MP at postpartum. It is important for researchers to consider this limitation when examining changes in MP, especially after participating in an intervention with a sample of first-time parents.

There are more existing interventions focused on promoting MP than PRF. As such, more research is needed to examine how combining the strengths of MP and PRF informs parenting experiences and impacts the quality of parent-child relationships. There is empirical value in this integration, especially if changes or improvements in parenting behaviors via parenting reflective processes or capacities (MP and PRF) can positively enhance parent-child relationship quality. Interventions that aim to enhance both MP and PRF to promote positive parent-child relationships may yield impactful results as PRF can be promoted as early as the prenatal period. For example, Pajulo et al. (2008) found prenatal PRF was positively associated with maternal sensitivity at four months. Given the extensive evidence base of MP interventions and complementary processes, combining the two together in one intervention may yield effects greater than either one can alone.

Several gaps in the MP and PRF literature were identified through this scoping review. First, across both bodies of research, the sampling of fathers was lacking. Understanding how mothers, fathers, and gender-expansive parents may engage in MP and PRF differently is important. Compared to mothers, fathers tend to report lower levels of MP (Medeiros et al., 2016; Moreira & Canavarro, 2018b). However, studies found fathers also benefit from practicing MP. For example, Gershy et al. (2017) revealed that fathers of schoolage children who developed interpersonal mindfulness skills were more likely to report improvement in the capacity for emotion regulation, reduced negative feelings, and reduced parental submission. For example, studies have also demonstrated that fathers’ MP is associated with greater emotional awareness of the child (Coatsworth et al., 2018) and less dismissive responses to the child’s emotions (McKee et al., 2018). No research among parents identifying with a genderexpansive parenting role was found in our review, highlighting a major gap in both bodies of literature.

From the PRF literature, Benbassat and Priel’s (2014) review revealed that fathers tend to score lower than mothers on reflective functioning, although other recent studies have found no differences (Borelli et al., 2016). Benbassat and Priel (2014) also reported that fathers’ reflective functioning is particularly important during adolescence as it is inversely correlated with adolescent behavior problems. However, more research that includes additional child outcomes and at different developmental stages is needed. It would be useful to identify whether reflective functioning in mothers and fathers is linked to the same parent and child outcomes. For example, Buttitta et al. (2019) found that fathers’ reflective functioning was linked to specific types of sensitivity, such as autonomy-supporting behaviors, which may be qualitatively different from mothers’ sensitive behaviors. Moreover, an unexplored research question is “How does mindful parenting differ based on parent gender identity/gendered parenting role (e.g., mother, father)? Rigorous assessment of MP and PRF in diverse samples of parents is necessary to answer this research question.

Diversifying the sample of caregivers continues to be a limitation as only 1.5% of the MP articles studied adoptive or stepparents, and an additional 2.7% of studies included a combination of parents with caregivers or teachers. In the PRF articles, only 4% studied adoptive or foster parents, and only one study included a combination of mothers, fathers, and foster parents. This limitation is concerning as family structure continues to change and family members such as grandparents, which some studies sampled, can take on the primary caregiver role in the family.

Another major sample limitation is the lack of racial and ethnic diversity. Surprisingly, over one-third of the studies (*n* = 111) did not even report the racial/ethnic makeup of their sample. Best standards in clinical trial reporting (e.g., CONSORT guidelines; Moher et al., 2012) require this level of detail. Particularly with the entrenched existence of racial/ethnic disparities due to systemic oppression and racism, research highlighting the potential benefit of MP and PRF for minoritized communities would be highly beneficial. Further, determining the cultural fit of mindfulness interventions for racial/ethnic minoritized communities is essential to creating effective interventions and understanding their impact (Black & Switzer, 2018). Among the studies that reported on the racial/ethnic makeup of their participants, the MP literature was more diverse than the PRF literature. However, there was little attention to the potential for iatrogenic effects or cultural mismatch of intervention approach with participants’ parenting values. A serious area of caution in parenting research led primarily by white researchers with predominantly white samples is an overgeneralization of parenting values based on white cultural ideology. Much could be learned by engaging scholars with expertise and life experience as members of Black, Indigenous, Latinx, and other communities of color in this area of inquiry. Questions in this line of research could include consideration of other longstanding cultural traditions that emphasize reflective capacities that go beyond MP and PRF.

Both lines of work could also benefit from more rigorous assessments of their instruments. For example, despite sixteen studies from our review that employed two PRF measures simultaneously in their research, as of 2020, no studies had empirically compared these various PRF instruments. This is a critical next step in the reflective functioning literature, as these instruments may assess overlapping but distinct constructs, similar to the differences between self-reported and interview-based measures of attachment (Roisman et al., 2007). For MP, there are currently two primary self-report assessments (IM-P; MIPQ), but no interview measures exist, and the only observational coding system for MP (Geier, 2012) has not been adopted in the field. Perhaps one benefit of looking at these two constructs together is that researchers can identify the extent to which MP could be assessed in interview-based assessments, such as the Parent Development Interview-Revised (Slade et al., 2004), or another interview created specifically for MP. The IM-P has been linked with both mother-infant stress physiology (Laurent et al., 2017) and parent-adolescent communication assessed through observational methods (Duncan et al., 2015).

### Limitations and Future Directions

Taken together, the studies reviewed here provide support for the critical influences of MP and PRF on the quality of parenting. Several strengths of this scoping review should be noted. To date, researchers have primarily investigated and viewed these two strands of parental reflective capacities separately. Hence, this is the first scoping review that comprehensively summarizes the MP and PRF literatures together to provide an initial conceptual link between these processes within a parenting framework. Ideally, the results from this review may bring potentially “siloed” MP and PRF functioning researchers’ attention to the existence of the other area of research. Second, our scoping review identified and summarized the distinct and overlapping empirical outcomes from MP and PRF studies published from 2005 to early 2020. Therefore, the results of this review can be used to identify future directions that address the existing gaps in the literature and present opportunities for further investigations (see the previous section). Lastly, this review highlights areas of growth for both MP and PRF research that may inspire new and important lines of work within these individual areas, such as the critical need to study more racially and ethnically diverse populations and to expand the research beyond mothers.

This scoping review is not without limitations. First, PRF is the only mentalization component focused on in this review. There are two additional components of mentalization—parental insightfulness and mind-mindedness—that were intentionally excluded from this scoping review. Future reviews may include these components to fully distinguish all the mentalization components from one another and their specific effects on parenting. Likewise, expanding the content search terms to include terms such as “parental insightfulness” and “parental mind-mindedness” is important to capture the full range of the existing mentalization literature. Second, this scoping review examined articles on PRF, not child reflective functioning. Thus, future reviews may include child reflective functioning as well as greater emphasis on child reports of parents’ MP (Coatsworth et al., 2015), given the bidirectional nature of the parent-child relationship. Lastly, we endorse the critical importance and value of qualitative research despite qualitative studies were beyond the scope of this review. Qualitative research is critically needed to understand more complex research questions and variables related to parenting.

Nonetheless, findings from this scoping review provide empirical evidence that MP and PRF play important roles in parenting. Specifically, our results support our suggestion of investigating these constructs concurrently as the evidence reported in our study establishes links between MP and PRF, overlapping outcomes, and independent contributions to parenting. The parent-child relationship is dynamic, and the quality of these interactions impact children’s emotional development and well-being over the short- and long-term. Thus, MP and PRF may be a critical faculty that promotes responsive caregiving as greater MP and PRF is associated with more positive parenting behaviors (Gershy et al., 2017; Krink et al., 2018) and higher quality parent-child interactions (Coatsworth et al., 2010; León et al., 2018). Our ability to understand and enhance parenting through intervention may be advanced when these two distinct streams of parenting research come together.

MP and PRF have different areas of focus and approaches, despite a similar goals of improving parenting and enhancing the parent-child relationship. MP emphasizes mindfulness practices to promote awareness, emotional regulation, and compassionate parenting in the present moment. PRF, on the other hand, is a psychological concept that specifically relates to a parent’s capacity to understand and reflect on their child’s inner world, with a focus on the child’s mental and emotional experiences. Both can be valuable tools for effective and nurturing parenting, and they likely complement each other in helping parents better connect with and support their children through reflective processes.

## Supplementary Material

Supplementary Materials 1

Supplementary Materials 2

## Figures and Tables

**Table 1:** Content analysis of mindful parenting articles (*N* = 180)

Study	Sample	MP Measure	RCT Design (Yes/No)	Other Variable(s)	Results

Minor et al. (2006)	44 Canada parents/caregivers of children with special needs and various chronic conditions	None	Yes	Intervention Maternal stress and mood	Significant improvement in stress and mood were found following the program
Singh et al. (2006)	3 mothers of children with Autism and their children	SUUM	Yes	Intervention Maternal satisfaction with their parenting, mother- child interaction, and use of mindfulness Child aggression, noncompliance, and self-injury	Mother’s satisfaction with their parenting skill and their interaction with children increased from baseline during mindfulness training Use of mindfulness was high on baseline, decreased during mindfulness training, and peaked during mindfulness practice Children’s aggression and maladaptive behavior decreased during and after the mindfulness train-
Dawe and Harnett (2007)	64 Australia parents on methadone maintenance	None	Yes	Intervention Parenting stress, child abuse potential Child behavior	Families that went through treatment showed reduction in child abuse potential, rigid parenting attitudes, and child behavior problems at follow-up
Maloney and Altmaier (2007)	12 U.S. divorced parents with their preschool children (100% White)	TMS	Yes	Intervention Mindfulness Parent-child connectedness	Participants reported an increase in mindfulness over the course of program. More time engaged in mindfulness practice during the study was not necessarily associated with higher posttreatment mindfulness scores. No change in parent-child connectedness was found.
Singh et al. (2007)	4 mother-child dyads	SUUM	Yes	Intervention Maternal parenting and interaction satisfaction, and use of mindfulness Child aggressive behavior and social interaction	Minimal improvement of child behavior and social interaction was found from baseline during the mindfulness training, and much more improvement occurred during the mindfulness practice phase. Parenting outcomes were low during baseline, increased during mindfulness training, and reached high levels during mindfulness practice.
Bögels et al. (2008)	14 Netherlands parents of adolescents with externalizing disorder	None	Yes	Intervention Personal goals Quality of life	Parents reported improvement in their own goals at posttest and follow-up Borderline improvement in quality of life at posttest
Lloyd and Hastings (2008)	148 England mothers of children [time 1:91, time 2: 57] with ID	MAAS	No	Family deprivation Maternal psychological acceptance, mindfulness, avoidant coping, positive perception of child, and mental well-being Child problem and adaptive behavior	Acceptance was negatively associated with maternal anxiety, depression, and stress, and bidirectionally related to anxiety and depression Mindfulness not significantly associated with any of the variables
Duncan et al., (2009a,2009b)	9 U.S. parents of children in 6^th^ grade (93% White)	None	Yes	Intervention Parent receptivity and perceived changes in behavior	Parents reported liking the new mindfulness activities in MSFP 10–14, but did not like the guided practice as much as other activities Parents reported greater awareness of how their moods affect how they react and an increased rate of stopping and thinking before reacting
Coatsworth et al. (2010)	65 U.S. mothers of youth from 10 to 14 (98% White)	IM-P	Yes	Intervention Child management Parent-youth relationship quality	Youth’s positive affect/behavior toward mother, youth-reports of discipline consistency, and monitoring improved in MSFP 10–14 relative to SFP 10–14 Mindful parenting served as an indirect effect between group and several outcomes
Duncan and Bardacke (2010)	27 pregnant women (89% White)	FFMQ	Yes	Intervention Maternal stress, pregnancy anxiety, depression, and positive and negative affect	Significant changes at post-test for almost all outcomes Qualitative results suggested themes of using informal and formal practices to cope with stress related to pregnancy, childbirth, parenting
MacDonald and Hastings (2010)	105 Ireland fathers of children with ID (94% Irish)	IM-P	No	Father’s involvement in childcare	Mindful parenting significantly predicts fathers’ involvement in child-related parenting task and socialization task, but not daily caregiving task.
Singh et al. (2010)	2 mothers and their children with ADHD	None	Yes	Intervention Maternal satisfaction with self in interaction with child and happiness with child Child compliance	Mothers reported increase in satisfaction with the interaction with their child and happiness in parenting Child compliance with mothers’ requests increased after mother training and even more after child training
Williams and Wahler (2010)	40 U.S. mothers of children with externalizing and internalizing problems (95% White)	MAAS	No	Parenting style Child problems	Mindfulness positively related to authoritative parenting and negatively to authoritarian and total child problems
Bluth and Wahler (2011a)	118 U.S. mothers of middle school adolescents (Majority being White)	MAAS	No	Parenting effort Youth externalizing and internalizing problems	Mother’s mindfulness was inversely correlated with perceptions of youth problem behavior Maternal mindfulness mediates the linkage between mother’s effort and their perception of youth internalizing problems and moderates the connection between mother’s effort and youth externalizing problems
Bluth and Wahler (2011b)	50 U.S. mothers of preschooler (82% White)	MAAS	No	Parenting effort	Negative correlation was found between mindfulness and parenting effort
Parent et al. (2011)	162 parents with history of depression (81.5% White)	MAAS	No	Parent mental health, observed positive and negative parenting Child externalizing and internalizing problems	Mindfulness was negatively associated with depressive symptoms, child internalizing problems, and child externalizing problems Mindfulness was not related to positive or negative parenting behavior
Srivastava et al. (2011)	60 India parents and children with behavioral problems	None	Yes	Intervention Child behavior problems	Children’s behavior problem significantly improved after parents’ participation in mindful parenting training
Benn et al. (2012)	60 U.S. parents and teachers of children with special needs (11 % of minority status)	FFMQ	Yes	Intervention	Mindfulness increased for treatment group at post and follow-up Also showed impacts on both positive & negative well-being
van de Weijer-Bergsma et al. (2012)	10 Netherlands parents of adolescents with ADHD	MAAS	Yes	Intervention Parenting stress, parenting style Adolescent functioning (attention, impulsivity, and behavioral problems)	Adolescents’ attention and behavioral problem reduced while executive functioning improved after mindfulness training Parenting stress decreased for fathers at post-test and follow-up Mothers reported decrease in parenting overreactivity but fathers reported increase at posttest
van der Oord et al. (2012)	22 Netherlands parents of children with ADHD	MAAS	Yes	Intervention Parenting stress, ineffective discipline style, parents’ own ADHD symptoms Child ODD symptom	Parents’ inattention, hyperactivity symptoms, and children’s ADHD symptoms significantly reduced after training and reduction maintained in follow-up Significant reduction in overreactive parenting and parenting stress were found from pre to follow-up
Beer et al. (2013)	28 Australia parents of children with ASD (85.7% White)	IM-P	No	Parent depressive and anxiety symptom, parenting stress Child behavior problems	Higher levels of MP were related to lower levels of depressive symptoms and parental stress MP was significantly negatively related to children’s’ behavior problems; however, it did not mediate the relationship between child behavior problems and parental outcomes
Ferraioli and Harris (2013)	15 U.S. parents of children with ASD (33% White, 27% Indian, 13% Asian, 13% Latina)	MAAS	Yes	Intervention Parenting stress and general health	Mindfulness group demonstrated significantly more change in parenting stress and general health at T2, and general health at T3 Mindfulness increased from T1 to T2 for mindfulness group only
Perez-Blasco et al. (2013)	26 Spain breast-feeding mothers	FFMQ	Yes	Intervention Maternal self-efficacy and self-compassion	Intervention group showed improvements in maternal self-efficacy, mindfulness (except describing), some self-compassion sub-scales, anxiety, stress, and psychological distress, but not in depression, satisfaction with life, or subjective happiness
Bögels et al. (2014)	86 Netherlands parents referred to a child/youth secondary mental health care (81% born in Netherlands)	None	Yes	Intervention Parent externalizing and internalizing problems, parental stress, parenting style, coparenting behavior, and marital conflict satisfaction Child externalizing and internalizing problems	Parental stress, parenting style (encouragement, overprotection, rejection, acceptance), and coparenting behavior improved after intervention Both children’s and parents’ externalizing and internalizing problems significantly decreased, and these improvements were maintained at 8-week follow-up
Conner and White (2014)	67 U.S. mothers of children with ASD and 87 mothers of children without ASD (94% White)	MAAS	No	Group (ASD or non-ASD) Parental stress, depression, and anxiety Child aberrant behavior	Mindfulness was negatively correlated with ASD diagnosis, stress, depression anxiety stress scale, aberrant child behavior Mindfulness was associated with lower stress in both ASD & non-ASD groups
Goodman et al. (2014)	23 U.S. pregnant women with anxiety symptoms (75% White)	MAAS	Yes	Intervention Maternal stress, depression, psychiatric symptoms, and self-compassion	Significant improvement in all outcomes were reported following intervention
Jones et al. (2014)	140 U.S. parents of children with Autism (90% White)	FFMQ BMPS	No	Parents’ psychological wellbeing Child behavior problems	Psychological acceptance mediated the relationship between child behavior problems and parental well-being (maternal anxiety, depression, stress and paternal depression) Both dispositional mindfulness and MP had mediating effects in relation to maternal anxiety, depression and stress Parents who reported increased mindfulness and acceptance also reported greater levels of positivity in relation to their child’s ASD
Neece (2014)	46 U.S. parents of children with development delay (26% White, 37% Hispanic, 23% Other)	None	Yes	Intervention Parenting stress, depression, and life satisfaction Child behavior	Parents who participated in MBSR reported less stress and depression and greater life satisfaction compared to waitlist control Children whose parents participated in MBSR reported fewer behavior problem
Parent et al. (2014)	121 U.S. black cohabiting low-income stepfamilies (Either mother or partner identifies as Black)	FFMQ	No	Firm control parenting Dyadic relationship quality	Mindfulness was directly related to each individual’s own perceptions of relationship quality Maternal mindfulness was indirectly related to maternal parenting practices through her perceptions of dyadic relationship quality
Singh et al. (2014)	3 mother-adolescent dyads of adolescent with ASD	None	Yes	Intervention Parents’ behavioral skills with their adolescents challenging behavior, and parenting stress	Adolescent’s challenging behaviors decreased and compliance behaviors increased commensurate with the mothers’ training in MBPBS
Bakhshayesh et al. (2015)	36 Iran children with ADHD and their mothers	None	Yes	Intervention Parenting style and parenting stress Child ADHD symptoms	Parents show reduction in parenting stress, inefficient interactions of parent-child, and these were reduction maintained at follow-up Children’s ADHD symptoms reduced after mindfulness training
Bazzano et al. (2015)	66 U.S. parents and caregivers of children with DD (45% Hispanic, 32% White, 12% Black)	MAAS	Yes	Intervention Parental stress, psychological well-being, self-compassion, and self-reported physical health	Significant lasting improvements were found in all measured outcomes after treatment
Coatsworth et al. (2015)	432 U.S. families with adolescents (69% White, 15% Black, 8% Latino)	IM-P	Yes	Intervention Parent well-being and parent- youth relationship Youth behavior management	Mothers and fathers in MSFP showed greater improvements in interpersonal mindfulness in parenting, parent-youth relationship quality, youth behavior management, and parent well-being compared to parents in SFP 10–14
de Bruin et al. (2015)	23 Netherlands adolescents with ASD and their parents	FFMQ IM-P	Yes	Intervention Parenting stress, and quality of life Youth’s ASD symptoms, mindfulness, worry, rumination, and quality of life	Adolescents reported an increase in quality of life and decrease in rumination, but no changes in worry, ASD core symptoms, or mindful awareness Parents reported improved in overall outcomes and increase in parental mindfulness
Duncan et al. (2015)	375 U.S. mothers of adolescents (88% White)	IM-P (brief 10-items)	No	Interactions between mothers and youth	MP was significantly related to greater warmth, positive interaction, positive parenting, consistent discipline, parent communication skills and lower harsh parenting
Eames et al. (2015)	23 UK socioeconomically disadvantaged mothers	FFMQ	Yes	Intervention Parental stress, depression, rumination, and mental well-being	Moderate to large effect size improvements were found in parental stress, depression and rumination after intervention
Geurtzen et al. (2015)	901 Netherlands adolescents and parents (95% Dutch)	IM-P	No	Parent depressive and anxiety symptoms, and tradition parenting dimensions Youth depressive and anxiety symptoms	MP was associated with adolescents’ symptoms of depression and anxiety while controlling for traditional parenting dimensions Non-judgmental acceptance was associated with lower levels of adolescents’ internalizing problems
Haydicky et al. (2015)	18 Canada adolescents with ADHD and their parents (59%’ parents born in North America)	IM-P	Yes	Intervention Parenting stress Family functioning Adolescents’ attention, externalizing and internalizing problems	Reduction in adolescents’ inattention, conduct problems, and peer relation problems were found after intervention Parents reported a decrease in parenting stress and increase in MP
Hwang et al. (2015)	6 Australia mother-child dyads of children with ASD	FMI	Yes	Intervention Parenting stress and quality of life	Overall improvement was found in all outcomes following intervention
Lewallen and Neece (2015)	24 U.S. mothers of children with DD (37% Hispanic, 33% White)	None	Yes	Intervention Child social skills	Secondary informants and teachers reported improvement in child self-control after intervention Mothers and teachers reported improvement in child empathy and engagement
Lippold et al. (2015)	432 U.S. mothers and their adolescents (72% White)	IM-P	No	Parental solicitation Adolescents’ perception of mother’ mindful parenting, and levels of disclosure	MP may promote parent-adolescent communication by reducing parental negative reactions to information, adolescent perception of over-control, and by improving the affective quality of parent-adolescent relationship Negative parental reactions to adolescent’s disclosure and adolescents’ perceptions of the quality of mother-adolescent relationship mediates the association between MP and adolescent disclosure and parental solicitation
Lunsky et al. (2015)	22 Canada parents of adoles- cents/adults with IDD	BMPS	Yes	Intervention Parental stress	Parents reported a significant reduction in parental stress after intervention, however no significant change in mindfulness or MP were reported
Meamar et al. (2015)	50 Iran mothers of adolescent girls	None	Yes	Intervention Maternal self-regulation Adolescents’ externalizing behavior problems	Maternal self-regulation and adolescents’ externalizing behavior problems improved after treatment
Moreira and Canavarro (2015)	439 Portugal parents	IM-P	No	Attachment anxiety and avoidance	Mothers reported significantly higher levels of MP than fathers Attachment-related anxiety and avoidance were indirectly associated with MP through different aspects of caregiving representations
Roberts and Neece (2015)	43 U.S. parents of children with DD (63% of minority status)	FFMQ	Yes	Intervention Feasibility of standardized MBSR intervention	Intensive MBSR intervention is feasible to parents of children with DD
van den Heuvel et al. (2015)	90 Netherlands mother-infant dyads	FMI	No	Maternal anxiety Infant socioemotional development and temperament	Higher maternal mindfulness during pregnancy was associated with less infant self-regulation problems and less infant negative affectivity Maternal anxiety mediated the association between infant self-regulation problems and maternal mindfulness
Corthorn and Milicic (2016)	62 Chile mothers of preschooler	IM-P FFMQ	No	Parental stress, general stress, and depression	Mindfulness were more strongly and consistenly related to more general aspects of mother’s mental health, while MP more strongly and consistently related to aspects of stress specific to their role as a mother, parent-child interactions, and perceptions about their child Being nonjudgmental about self as a person and mother appeared as main predictive aspect of MP on overall outcome
Gouveia et al. (2016)	333 Portugal parents	IM-P MAAS	No	Parenting stress, parenting style, and self-compassion	Higher levels of dispositional mindfulness and self-compassion are associated with higher levels of MP that, in turn, is associated with lower levels of parenting stress, higher levels of authoritative parenting style, and lower levels of authoritarian and permissive parenting styles
Mann et al. (2016)	12 UK parents for manual development (100% White) and 38 parents with history of depression in intervention condition (97% White)	FFMQ	Yes	Intervention Parental stress, depression Child psychopathology	Participants randomized to MBCT-P have greater reduction in their depressive symptoms compared to usual care over 9-months follow-up Increase in mindfulness and self-compassion was found and an initial reduction in their child’s psychopathology at 4 months
May et al. (2016)	18 U.S. parents of adolescents	FFMQ	Yes	Intervention Parental neural activation, perceived stress Child reports of family relationship	Parents reported significant increase in mindfulness and decrease in stress while children reported increase in the amount of parent monitoring after intervention, however no change in positive family relationship was reported Parent neural activation from pre to post intervention increased in area related to self-awareness and evaluation, emotional awareness and interception, and emotion regulation
Medeiros et al. (2016)	243 Portugal family triads	IM-P	No	Children’s well-being	Mothers reported higher levels of MP than fathers MP of mothers were positively correlated with MP of fathers Child’s perception of security in the relationship with their parents mediated the link between the parents’ MP and child’s well-being
Meppelink et al. (2016)	70 Netherlands parents (80% Dutch)	IM-P FFMQ	Yes	Intervention Parent and child psychopathology	Significant decrease in parents’ and children’s psychopathology and an increase in MP and general mindfulness was found after intervention Changes in parents’ general mindfulness predicted a decline in parental psychopathology, but not MP MP significantly reduced children’s externalizing problems reported by parents.
Moreira et al. (2016)	290 Portugal mothers	IM-P	No	Maternal attachment avoidance and attachment anxiety	Self-compassion mediated the relationship between attachment anxiety and MP, in that higher levels of anxiety was associated with lower SC, which in turn was associated with lower levels of MP
Parent et al. (2016a, b)	485 U.S. parents of children from three developmental stages (79% White)	IM-P MAAS	No	Negative and positive parenting Coparenting relationship quality	Findings across all three youth development stages indicated both direct effects of parent dispositional mindfulness as well as indirect effect through MP and mindful coparenting, with parenting and coparenting relationship quality
Parent et al. (2016b)	615 U.S. parents of children from three developmental stages (72–80% White across three developmental stages)	IM-P MAAS	No	Child externalizing and internalizing problems	Consistent findings across all three developmental stages Higher parent dispositional mindfulness was indirectly related to lower youth internalizing and externalizing problems through higher MP and lower negative parenting practices
Rayan and Ahmad (2017)	104 Jordan parents of children with ASD	MAAS	Yes	Intervention Parents’ quality of life and positive stress reappraisal	Parents in the intervention group showed improvement in psychological and social health domain of quality of life, mindfulness, and positive stress reappraisal with medium to large effect size after intervention. Parents in control group improved in overall outcomes with small effect size.
Serkel-Schrama et al. (2016)	215 Netherlands parents and 129 of their adolescents (Majority Dutch)	IM-P	No	Child glycemic control and quality of life	MP is related to more optimal glycemic control in adolescent boys, adolescent girls who were not hospitalized in the last 12 months, and to proxy- reported generic and diabetes-specific quality of life of both male and female adolescents with T1DM No significant associations wwere found between MP and generic or diabetes-specific quality of life as rated by the adolescents by themselves Significant association was found between MP and glycaemic control and hospitalization due to ketoacidosis
Siu et al. (2016)	216 China mothers	MAAS	No	Mother-child relationship Child social adjustment	Maternal mindfulness had positive effect on attachment/involvement/parental confidence and negative effect on discipline practice/relational frustration Maternal mindfulness had a negative indirect effect to children’s emotional symptoms, conduct problems and positive indirect effect to children’s prosocial behavior
Turpyn and Chaplin (2016)	157 U.S. adolescents and their primary caregiver (64% White, 14% Black)	IM-P	No	Parent negative and positive expression during conflict interaction with children Adolesents’ risk behavior	MP was related to less parent al negative emotion expression in the conflict interaction and greater shared parent-adolescent positive emotions Significant indirect effect of MP on youth’s substance use through shared parent-adolescent positive emotion was found
Waters (2016)	68 Australia parent-child dyads	CAMS-R	No	Child stress	Both parent mindfulness and child mindfulness are negatively related to child stress Child mindfulness did not mediate the relationship between parent mindfulness and child stress
Zarnegar et al. (2016)	10 U.S. adopted maltreated children with fetal alcohol spectrum disorder (FASD) and their caregiver (80% White)	None	Yes	Intervention Parenting stress Child development and functional capacity	Children’s functioning and parenting stress improved from pre-post intervention Early intervention that is tailored to children’s neuropsychological strengths and deficit may hold promise for promoting global improvements
Campbell et al. (2017)	128 U.S. adult parents of children under 18 (82% White)	FFMQ	No	Parenting stress, and parental responsiveness	The more mindful parents are, the more attuned and responsive they are to their child’s needs, and that this is explained by the lower levels of parenting stress associated with higher levels of mindfulness Results also indicated particular importance of the recursive relational aspect (i.e., parent-child interaction) of the constructs
Chan and Lam (2017)	271 Hong Kong parents of children with ID (100% Hong Kong Chinese)	CAMS-R	No	Parental stress Child behavior problems	Parental mindfulness partially mediated the association between parent-reported child behavior problems and parental stress Medium or high level of parental mindfulness buffer the effect of child behavior problems on parental stress
Dehkordian et al. (2017)	60 Iran adolescents with ADHD and their parents	None	Yes	Intervention Child quality of life	MP improved the quality of life of children with ADHD
Duncan et al. (2017)	30 U.S. first-time mothers in the late 3^rd^ trimester (59% White, 18% Latina/Hispanic, 17% Asian)	FFMQ MAIA	Yes	Intervention Perinatal depression Childbirth-related appraisals and psychological functioning, use of pain medication in labor, and birth satisfaction	Mindfulness-based childbirth education improved women’s childbirth-related appraisals and psychological functioning in comparison to standard childbirth education Mothers showed greater childbirth self-efficacy and mindful body awareness (but no changes in dispositional mindfulness), lower post-course depression symptoms after intervention that were maintained through postpartum follow-up, and a trend toward a lower rate of opioid analgesia use in labor
Gannon et al. (2017)	160 U.S. women with opioid use disorder	IM-P	Yes	Intervention Quality of parenting behavior	Overall parenting quality improved from low quality at baseline to moderate quality at program completion There was also improvement in overall quality of parenting behaviors at a greater rate over time
Gershy et al. (2017)	152 Israel parents of children with ADHD and behavior problems	None	Yes	Intervention Parental emotion regulation, hostile and coercive parenting Child behavioral problems	Mothers’ negative feelings, escalating behaviors, and capacity for emotion regulation improved significantly following treatment. Mothers across treatment conditions reported reduced emotional dysregulation and coercive behaviors Fathers in the NVRM condition experienced a greater decrease in paternal emotional dysregulation following treatment than did fathers in the NVR condition. There was no change in coercive behavior in father over time in either treatment condition Parents across treatment conditions reported a significant reduction in child externalizing symptoms
Gurney-Smith et al. (2017)	39 UK parents (17 adoptive parents)	MAAS	Yes	Intervention Self-compassion Parent stress score, defensive responding, parental distress and perceived difficult child	Self-compassion and mindfulness for staff group increased from pre- to post- training, although this was not statistically significant for mindfulness Significant difference of mindfulness and self-compassion was found for adoptive parents from pre- to post- training Improvements were reported in total parent stress score, defensive responding, parental distress and perceived difficult child domains
Heifetz and Dyson (2017)	6 Canada adolescents with IDD and 8 of their parents	IM-P	Yes	Intervention Mood improvement of youth Social parenting	Youth participants showed more happy, relaxed, and less worried from pre- to post- sessions, while parents reported that their youth’s social behaviors showed a trend to have more social behavior post- group For parent, only participant 3 showed a great difference between pre- and post-group in MP Parents reported a range of benefits and positive changes through feedback surveys completed following the final group session
Korukcu and Kukulu (2017)	60 Turkey pregnant women with preterm premature rupture of membranes (PPROM)	None	Yes	Intervention Maternal attachment Post-partum self-evaluation	Improvement in acceptance of pregnancy, level of readiness to give birth, level of maternal attachment, and level of competence in the role of motherhood was found after intervention
Laurent et al. (2017)	73 U.S. mother-infant dyads (77% White)	IM-P-I FFMQ	No	Maternal dispositional mindfulness and stressful life events Mother’s and infant’s hypothalamic pituitary-adrenal (HPA) axis activity during a dyadic stressor	MP predicted steeper maternal HPA axis recovery slopes during early postpartum, but not dispositional mindfulness No main effect of maternal MP was found on infant cortisol
Lo et al. (2017)	100 Hong Kong children with ADHD symptoms and their parents (100% Chinese)	IM-P	Yes	Intervention Overall behaviors, and parenting stress and well-being	Families from intervention group had greater improvements in children’s ADHD symptoms, overall behaviors, and parenting stress and wellbeing than those in wait-list control group
Lo et al. (2017)	180 Hong Kong parents of children with DD (100% Chinese)	IM-P	Yes	Intervention Parental stress and stress from parent-child dysfunctional interaction	Parents had significant improvements in parental stress and stress from parent-child dysfunctional interaction Parents with severe stress and depression reported more significant positive changes, and they reported moderate effect for stress and depression.
Lunsky et al. (2017)	50 Canada parents of adults with ASD	FFMQ BMPS	Yes	Intervention	Parents in the mindfulness group reported significant reductions in psychological distress, while parents in the support and information group did not Reduced levels of distress in the mindfulness group were maintained at 20 weeks follow-up
Maughan and Wiess (2017)	57 Canada parents of children with ASD (61% White)	IM-P (10-item)	Yes	Intervention Parents’ expressed emotion, negative parenting, parent mental health, emotion regulation Child mental health and psychopathology	Parents reported improvement in depression, emotion regulation, perceptions of their children, and MP after treatment
Miklósi et al. (2017)	145 parents (100% White)	MAAS	No	Perceived parenting, early maladaptive schemata and parental sense of competence	Higher levels of perceived aversive parenting are associated with stronger early maladaptive schemata, which in turn are related to lower levels of mindfulness, leading to lower levels of parental sense of competence
Potharst et al. (2017)	44 Netherlands mothers of infants (66% Dutch)	IM-P FFMQ	Yes	Intervention Maternal self-compassion, well-being, psychopathology, parenting stress, lack of confidence, warmth and negativity toward child Infant temperament	Mothers reported on increased MP, mindfulness, self-compassion during the training, and this improvement was maintained during 8-week follow-up Significant improvement was found in maternal well-being
Rayan and Ahmad (2017)	104 Jordan parents of children with ASD (100% Arabic speaking)	MAAS	Yes	Intervention Perceived stress, anxiety and depression	Parents in the intervention group had better outcomes on psychological well-being, mindfulness, and improvements in stress, anxiety, depression than those in the comparison group
Short et al. (2017)	59 U.S. women who are in treatment for substance use disorder (Majority White)	None	Yes	Intervention General and parenting stress	General stress decreased significantly from baseline to post-intervention Women with the highest baseline general stress level experienced the greatest change in total stress score.
Zhang et al. (2017)	11 Hong Kong children with ADHD and one of their parents (100% Chinese)	IM-P	Yes	Intervention Program feasibility Parenting stress and children’s attention, behavior, and executive function	MY mind was feasible and well accepted among children with ADHD and their parents as shown in course attendance, evaluation, and qualitative results Qualitative result showed the parents expressed very positive views on the MYmind course and admitted that they improved through the course. Most children also expressed positive views although some did not comment.
Aalders et al. (2018)	421 Netherlands parents of children with Type 1 Diabetes	FMI	No	Parental fear of hypoglycemia	Parents with an increased ability to be less judgmental of themselves as parents and less reactive to emotions within parenting interactions reported less fear of hypoglycaemia
Behbahani et al., (2018)	60 Iran children with ADHD	None	Yes	Intervention Parenting stress Child ADHD symptoms	MP training improved maternal parenting stress. Parents reported improvement attention, impulsivity, and attention deficit hyperactivity symptoms in their children after the training
Brown et al. (2018)	28 U.S. child welfare-involved parent with their children (64% White, 14% Latino, 14% Black)	FFMQ	Yes	Intervention Parental substance use Child maltreatment Family functioning	Intervention was found to reduce parenting stress, child abuse potential, and child behavior problems, and improve mindfulness
Chan and Neece (2018)	80 U.S. parents with children with DD (48% Latino, 25% White)	None	No	Parenting stress, parenting behavior Child emotion dysregulation	There was a reduction in parenting stress after MBSR MBSR significantly predicted reductions in child emotion dysregulation Intrusive parenting was found to significantly mediate the relationship between parenting stress and child emotion dysregulation
Chaplin et al. (2018)	83 U.S. mothers of adolescents (65% White, 12% Mixed-race, 10% Hispanic)	IM-P MAAS	Yes	Intervention Parenting stress, parent-adolescent relationship quality	Mindfulness intervention, compared to parent education increased mothers’ mindfulness, reduced parenting stress in two domains, increased MP related to emotional awareness in parenting, and improved parent-adolescent relationship quality For mothers of girls (but not mothers of boys), the mindfulness intervention also decreased negative parenting behavior and decreased negative emotional responses
Coatsworth et al. (2018)	432 U.S. families (69% White, 15% Black, 8% Latino)	IM-P	Yes	Intervention Positive parenting, parent-youth relationship quality Youth aggression	Changes in mothers’ and fathers’ MP was associated with increased positive parenting and parent-youth relationship quality Father’s MP was associated with decreased youth’s aggressive behaviors, but not mothers’ Fathers who participated in MSFP showed increase in emotional awareness of the child, compared to fathers in other conditions
Corthorn (2018)	43 Chile mothers of pre-schooler	IM-P FFMQ	Yes	Intervention Parental stress, depression, and anxiety	The intervention group showed a significant reduction in general and parental stress and an increase in MP and general mindfulness variables when compared with the comparison group, these improvements were maintained after 2 months
Gouveia et al. (2018a)	572 Portugal dyads of parent and child	IM-P	No	Adolescent’s emotional eating	MP were associated with lower levels of adolescents’ emotional eating through higher levels of adolescents’ self-compassion in isolation or followed by lower levels of body shame among girls but not boys
Gouveia et al. (2018b)	576 Portugal mothers of children with normal weight and 490 mothers of children with overweight	IM-P	No	Child body mass index (BMI)	Almost all mindful parenting dimensions and children’s zBMI were significantly associated with mothers’ concern and child-feeding practices
Hicks et al. (2018)	102 U.S. high-risk expectant parents due to high rates of violence exposure and psychopathology (59% Black, 27% White)	FFMQ	No	Parent depressive and trauma symptoms	Higher levels of mindfulness were associated with lower levels of depressive and trauma symptoms Levels of dispositional mindfulness (i.e., total mindfulness score and the non-reactivity subscale score) and current trauma symptoms were significantly associated with current depressive symptoms in the expected directions
Jastreboff et al. (2018)	62 U.S. parent-child dyads with parents with obesity (37% White, 63% Multiracial)	MAAS	Yes	Program feasibility Intervention Child obesity	Participants of mindfulness-bases parent stress group intervention plus nutrition and physical activity counseling showed greater improvement in parental involvement and decreased parental emotional eating Mindfulness-based parent stress intervention to decrease child- hood obesity risk is feasible
Jones et al. (2018)	21 UK parents of children with ASD, Down syndrome. Cerebral Palsy, ID (100% British)	FFMQ BMPS	Yes	Intervention General stress	Parent reported increases in mindfulness and selfcompassion, and reduced general stress, anxiety and depression No significant reductions in their child’s behavior problems or increases in the child’s prosocial behavior were found
Laurent et al. (2018)	25 U.S. mother-child dyads (72% White, 12% Latina)	FFMQ	No	mothers’ dispositional mindfulness and neural responses to their own infant in different emotion-eliciting contexts.	Mothers who reported higher Nonreactivity showed reduced signal in hypothesized regions: bilateral insula and prefrontal cortex (both dorsolateral and ventrolateral regions). They further showed lower signal across a range of cortical areas—bilateral temporal (including auditory regions and temporal pole), occipital (fusiform and lingual gyri), and parietal (including precuneus, supramarginal gyrus)—and subcortical regions (thalamus, right caudate). Mothers higher in nonjudging also showed reduced signal in parietal areas (right angular gyrus, bilateral precuneus extending to posterior cingulate cortex) and in the left dorsolateral prefrontal cortex. Mothers higher in describing showed increased signal in several occipital (left fusiform to lingual gyrus) and parietal (bilateral precuneus, right superior parietal extending to supramarginal gyrus) areas.
Lengua et al. (2018)	50 U.S. parent-child dyads	FFMQ-SF	Yes	Intervention young children’s self-regulation, social-emotional competence, and academic readiness parent mindfulness, self-regulation, and evidence-based parenting practices.	Parent self-reported executive function and observed scaffolding behaviors demonstrated a significant increase from pre-test to post-test, with effects sustained at follow-up, and significant decrease in rejection Children demonstrated significant decreases in observed negative affect, while parents reported significant increases in children’s social competence and academic readiness.
McKee et al. (2018)	246 U.S. parents (80% White)	IM-P	No	Child supportive and nonsupporative emotion socialization response	Higher levels of MP were associated with more supportive emotion socialization responses and fewer nonsupportive emotion socialization responses MP was more strongly linked with lower levels of parent distress, punitive, and dismissive responses to child emotions for fathers than mothers
Moreira and Canavarro (2018a)	265 Portugal mothers	IM-P	No	Maternal self-critical rumination, parenting stress	Higher levels of mothers’ self-critical rumination were associated with higher levels of parenting stress through 2 dimensions of MP (nonjudgmental acceptance and emotional awareness)
Moreira and Canavarro (2018b)	658 Portugal mother-adolescent dyads	IM-P	No	Adolescent dispositional mindfulness	Mothers with significant levels of anxiety and/or depressive symptoms Higher levels of MP were associated with higher levels of adolescents’ dispositional mindfulness, and these associations were moderated by mothers’ anxiety and/or depression symptoms
Moreira et al. (2018)	563 Portugal parent-child dyads	IM-P	No	Adolescent well-being	Adolescents’ attachment representations, self-compassion, and mindfulness mediated the association between MP and adolescents’ well-being
Potharst et al. (2018a)	98 Netherlands parents in clinical setting (90% Dutch) and 89 parents in nonclinical setting (88% Dutch)	IM-P	Yes	Intervention Parental stress, over reactivity, well-being, and partner relationship Child well-being and behavior problem	MP training was as effective in a non-clinical context as it is in a clinical context. Parents improved on parental stress, over reactivity, MP, well-being, and partner relationships, and children of these parents improved in behavior problems and well-being.
Potharst et al. (2018b)	18 Netherlands mother-toddler dyads experiencing (co-) regulation difficulties (68% Dutch, 23% non-European)	IM-P FFMQ-SF	Yes	Intervention maternal sensitivity and acceptance of the child, and mother-rated child dysregulation and psychopathology, maternal over reactivity, parenting stress, parenting sense of competence, maternal psychopathology, partner relationship.	There was no significant difference were seen in outcomes between waitlist and pretest assessment, except for a deterioration in listening with full attention and an improvement in compassion for the child Mothers were more sensitive and more accepting toward their child. Child psychopathology had decreased, and a borderline significant improvement in child dysregulation was revealed Maternal over reactivity did not improve significantly, while parenting stress a borderline significant improvement at post-test
Ridderinkhof et al. (2018)	45 Netherlands children with ASD and their parents	IM-P	Yes	Intervention Children’s and parents’ social communication problem, emotional and behavioral functioning, mindful awareness	There was no significant change in mindful awareness of children, but their social communication problems decreased, and their emotional and behavioral functioning improved There was an improvement on parents’ emotional and behavioral functioning, improved parenting, and mindful awareness on all occasions of pre-, post-, 2-month follow-up, and 1-year follow up, while social communication problems reduced only directly after the intervention
Ruskin et al. (2018)	34 Canada parents of adolescents with chronic pain	MAAS	Yes	Intervention Parent psychological flexibility Adolescent pain diagnosis Two-way interaction	There was a significant effect for parent psychological flexibility from pre to post intervention. There was no significant effect of adolescent pain diagnosis, nor two-way interaction.
Townshend et al. (2018)	109 Australia women at-risk for perinatal depression and anxiety (47% Australian, 21% non-English speaking background)	MAAS FFMQ	Yes	Intervention Perinatal depression and anxiety, general stress score	CBMP significantly reduced depression, anxiety, perinatal depression, perinatal anxiety and general stress scores, and significantly increased self-compassion and mindfulness with moderate to strong effect size.
Cowling and Van Gordon (2018*)	49 England parents of pre-schooler (97% White)	FFMQ	Yes	Intervention Parent psychological distress and parenting stress	There is a significant increase in mindfulness levels from pre- to post- three-day online mindfulness-based intervention There was also a significant decrease in both psychological distress and parenting stress.
Wang et al. (2018)	168 China mother-child dyads	IM-P	No	Adolescent emotional problems	Maternal MP may decrease adolescents’ emotional problems through adolescents’ perceived maternal warmth and their dispositional mindfulness.
Warriner et al. (2018)	155 UK parents	FFMQ	Yes	Intervention Maternal and paternal mental health	Pregnant women improved significantly post MBCP-4-NHS in terms of self-reported symptoms of perceived stress, anxiety, depression, pregnancy-related distress, labor worry and positive and negative pregnancy experiences. Fathers improved significantly post MBCP-4-NHS in self-reported symptoms of anxiety, depression and showed a trend for improvement in self- reported symptoms of perceived stress but this was not significant. There is a different baseline score but parents showed significant improvement after the course.
Wheeler et al. (2018)	155 U.S. mothers of children with Fragile X syndrome (90% White)	FFMQ-SF BMPS	No	Acceptance Maternal outcomes including stress, anxiety, depression, and health symptoms.	The severity of children’s disability significantly predicted each maternal outcome after controlling for total number of children in the family and where necessary, education and/or marital status. General mindfulness, acceptance and flexibility significantly predicted all maternal outcomes, while mindfulness in parenting was a significant predictor of stress, anxiety, and depression.
Whitlock et al. (2018)	196 U.S. parents with self-injuring youths and 57 parents of youths without no known mental health challenges (83% White in nonsuicidal self-injury sample and 64% White no known mental health history sample)	IM-P	No	Caregiver strain and factors that contribute to caregiver strain Parent expectancies for positive and negative outcome for self and child Parent-child relationship	Having a self-injuring youth adds significant strain to caregiver Nonjudgment of self and child was positively associated to objective stress but negatively related to subjective stress.
Wilson and Donachie (2018)	32 UK women with difficulties in emotion regulation (100% British)	None	Yes	Intervention Perinatal mental health	Decrease in overall psychological distress and increase in confidence in managing mental health and ability to cope with emotions were found after Dialectical Behavioral Therapy (DBT)
Zhang et al. (2018)	370 U.S. military parents from 207 families that had at least one parent who had been deployed overseas	FFMQ	Yes	Intervention Military parents’ online engagement Deployment status Dispositional mindfulness	Parents engaged with the exercises. Attendees and mothers engaged more than non-attendees. Engaged fathers were all deployed, engaged mothers were mostly non-deployed Parents’ dispositional mindfulness score was significantly higher
Benton et al. (2019)	30 U.S. mothers-adolescent dyads (80% White)	Adopted version of MPOS	Yes	Intervention Parent emotional availability Adolescent well-being and behavior problem	Strong correlations were found btw global scored of EA and MP. MP dimensions were significantly correlated to adolescent outcomes including life satisfaction and externalizing problems. Nonjudgmental acceptance of self and child is an MP dimension independently linked with indicators of adolescent externalizing problems, distinct from EA.
Cheung et al. (2019)	142 Hong Kong parents of children with ASD (100% Chinese)	BMPS	No	Parent affiliate stigma and mental well-beingChild adjustment	Parenting stress mediates the association btw parents’ disposition to MP/affiliated stigma and behavioral difficulties in children with ASD.
Emerson et al. (2019)	89 Netherlands parents of children assessing secondary mental health care (84% Dutch)	IM-P FFMQ	Yes	Intervention Parenting factors Child and parent psychopathology	Parent-reported child and parent psychopathology improved following the intervention. Improvements were found in the parenting factors targeted within the MP intervention. Increased MP predicted improvements in the child attention problem, but not mindfulness. Increase in MP did not predict improvement in child internalizing and externalizing problems.
Evans et al. (2019)	43 U.S. pregnant women with history of depression	None	Yes	Intervention Maternal depression	engagement being associated with depression improvement. improvements in depressive symptoms to the extent that they regularly attend classes and are engaged with the at-home practices pregnant women with at least two children practiced less often and attended fewer classes than women with no other children or only one child
Gouveia et al. (2019)	726 Portugal family dyads	IM-P	No	Child disordered eating behavior (emotional eating and overeating)	MP was negatively associated with children/adolescent’s emotional eating through lower levels of parenting stress followed by less frequent use of food as a reward as well as through parents’ less frequent use of food as a reward only MP was negatively associated with children’s overeating through lower levels of parenting stress, but not adolescents. MP was associated with more adaptive parental child-feeding practices through lower levels of parenting stress, among parents of girls from the early adolescents
Han et al. (2019)	2237 China parents (94% Han nationality)	IM-P FFMQ	No	Child externalizing and internalizing problems	Increase in parents’ dispositional mindfulness are negatively associated with children’s internalizing and externalizing problems through mindful parenting and subsequent parenting practices
Henrichs et al. (2019)	118 Netherlands mother-child dyads (96% Dutch)	IM-P	No	Maternal anxiety during pregnancy Child internalizing problems at age 4	MP mediated the association btw maternal state anxiety during pregnancy and child internalizing problems at age 4 The association btw maternal anxiety during pregnancy and child internalizing problems at age 4 was mediated by concurrent maternal general anxiety followed by MP
Hicks and Dayton (2019)	102 U.S. expectant parents (59% Black, 28% White)	FFMQ	No	Parents childhood trauma and trauma symptoms Child abuse potential	Non-reactivity was a significant predictor of potential risk of child abuse when controlling for childhood trauma history and current trauma symptoms.
Lippold et al. (2019)	421 U.S. mothers and their adolescents (73% White, 10% Black)	IM-P	Yes	Intervention Parenting cognition (sense of competence and parent- centered attribution) Parent-child communication	Parents who were more mindful were more likely to feel competent in their parenting role and to experience less self-blame Parents who felt more competent and had fewer negative parent-centered attributions were more likely to be mindful in parenting The effects of MP on parent-child communication were not mediated by changes in parenting cognition The effect of parenting cognitions on parent-child communication were mediated by MP
Moreira et al. (2019)	335 Portugal employed parents	IM-P	No	Work-family conflict	Higher levels of work-family conflict were indirectly associated with lower levels of mindful parenting dimensions through anxiety and depression symptoms and parenting stress
Neece et al. (2019)	80 U.S. parents of children with DD (35% White, 46% Latino, 9% Asian)	None	Yes	Intervention Parenting stress, depression, satisfaction with life Child behavior	MBSR improved parental mental health outcomes for Latino and non-Latino parents MBSR was associated with reduction in parent-reported child behavior problems
Pan et al. (2019)	104 Taiwan women between 14 and 28 weeks of gestation	FFMQ	Yes	Intervention Parental stress, depression Childbirth self-efficacy	MBCP was found effective in reducing self-perceived stress, depression, and in increasing mindfulness and childbirth efficacy
Park et al. (2020)	117 U.S. parents and adolescents (69% White)	IM-P	No	Adolescent externalizing and internalizing problems	Higher levels of mindful parenting were related to reduction in recurrent conflict after 3mth follow-up, and greater reduction in recurrent conflict during the 3mths were related to greater reduction in externalizing and internalizing problems over the following year
Potharst et al. (2019)	67 U.S. mothers	IM-P	Yes	Intervention Parental stress, over-reactive parenting discipline, depression and anxiety symptoms, self-compassion Child aggressive behavior and emotional reactivity	Online mindful parenting intervention was significantly more effective at 95% level than a waitlist period with regard to over-reactive parenting discipline, and symptoms of depression and anxiety Self-compassion, mother-rated child aggressive behavior and child emotional reactivity were significant at 90% level
Salem-Guirgis et al. (2019)	23 Canada parent-child dyads with child with Autism (71% White)	IM-P FFMQ	Yes	Intervention Parent mental health Youth mental health, mindfulness, Autism symptoms	Youth improved in autism symptoms, emotion regulation, and adaptive skills following the program Parents improved in mindfulness following the program
Seidman et al. (2019)	30 U.S. parents of children being treated for chronic pain (94% White)	MIPQ	No	Parental solicitousness, stress, resilience	Significant decrease was found in parental solicitous behavior and perceived stress, and increase in mindful parenting after 30-day mindfulness curriculum through mobile app
Singh et al. (2006)	47 mothers of adolescent with ASD and 45 mothers of adolescent with ID	None	Yes	Intervention Maternal mediation practice, perceived stress Child aggressive and disruptive behavior, and compliance with mother’s request	Significant reduction in levels of stress was found in both groups of mothers. Significant reduction in aggression and disruptive behavior and increase in compliance was found in adolescents in both groups.
Turpyn et al. (2019)	20 U.S. high-stressed mothers of adolescents (55% White, 20% Black)	IM-P	Yes	Intervention Maternal emotion reactivity, negative emotion, salivary cortisol reactivity, and fMRI emotion task and fMRI resting task scan	Mindfulness intervention increased brain responsivity in the left posterior insula in response to negative affective stimuli, and altered resting state functional connectivity in regions involved in self-reference, behavioral regulation, and social-emotional processing Changes in mothers’ brain function and connectivity were associated with increased mindful parenting and decreased emotional reactivity to the parent-adolescent conflict task
Van Gampelaere et al. (2019)	56 Belgium parents of 40 children with Type 1 Diabetes (96% Belgian)	MAAS	No	Parent’s daily worries and protective parenting behaviors	Mindfulness emerged as a buffer against daily worries and maladaptive parenting Mindfulness moderated the association between parental worries and protective behavior
Wong et al. (2019)	63 Netherlands mother-child dyads	IM-P	No	Children’s social decision making	Higher MP significantly predicted more sharing behaviors in children No effect was of MP was found for any of the individual decision-making measures
Zeegers et al. (2019)	50 Netherlands mothers with mood/anxiety/stress disorder and other disorders (12% Dutch)	None	Yes	Intervention Parenting stress, sensitivity and acceptance, and mind- mindedness Dyadic synchrony between mother and child	Mothers reported less parenting stress, more accepting and made less nonattuned references to the child’s mental state after training Child showed higher levels of responsiveness after training
Zhang et al. (2019a)	313 U.S. mothers who were either deployed or had partner who were deployed (91% White)	FFMQ	Yes	Intervention Self-reported parenting skills and observed parenting skills	Trait mindfulness was a moderator the intervention effect Mothers with lower levels of baseline mindfulness reported higher mindfulness at 1-yrs follow-up in intervention condition, while mothers with average mindfulness did not report changes at follow-up in both conditions Mindfulness at 1 or 2 yr follow-up was associated with self-reported parenting skills at 2 yr followup but not with observed parenting skills
Zhang et al. (2019b)	472 China parents	IM-P MAAS	No	Child emotion regulation	Higher levels of dispositional mindfulness were linked to greater MP, which in turn promotes secure attachment btw parent and child, thereby contributing to children’s lower emotion lability/ negativity and higher adaptive emotion regulation MP and parent-child attachment mediated the association btw parents’ dispositional mindfulness and children’s emotion regulation
Gouveia et al. (2019)	379 Portugal mother-child/adolescent dyads	IM-P	No	Mothers’ difficulties in emotion regulation Mindful parenting Children/adolescent’s emotional eating and depressive symptoms	Higher levels of mothers’ difficulties in emotion regulation are associated with higher levels of children/adolescents’ depressive symptoms through lower levels of mindful parenting. Lower levels of mindful parenting skills are associated with higher levels of children/adolescents’ emotional eating through higher levels of children/adolescents’ depressive symptoms.
Liu et al. (2019)	China STUDY 1: 272 middle school students STUDY 2: 525 adolescent- mother dyads	IM-P	No	Perceived mindful parenting (adolescent report) Mindful parenting (caregiver report) Adolescent dispositional greed Core self-evaluation.	Both mindful parenting perceived by adolescents and mindful parenting reported by primary caregivers has significant impacts on adolescent dispositional greed, and the relationship is mediated by adolescent core self-evaluations.
Eltelt and Mostafa (2019)	100 Egypt pregnant women	MAAS	Yes	Intervention Parental stress	Mothers in intervention group reported significantly reduction in the levels of perceived stress during pregnancy
Hunter et al. (2019)	18 U.S. women with FMR1 premutation (PM) and are mothers of children with fragile X syndrome (FXS)	Not measured	Yes	Intervention Use of App-based Mindfulness Exercise Maternal stress and social anxiety	Mothers with social anxiety and those experiencing barriers to social support were more likely to find the program helpful.
Pan et al. (2019b)	74 Taiwan women between 13 and 28-weeks gestation	FFMQ	Yes	Intervention Depression Stress	Mothers in the intervention group had a significant reduction in self-reported depression and stress than the comparison group.
Price et al. (2019)	12 U.S. pregnant women with a history of sexual trauma	FFMQ-SF	Yes	Intervention feasibility Mindfulness Health outcomes	MBCP intervention is effective for women with a history of sexual trauma. Mothers showed significant reduction in prenatal anxiety following the intervention.
Raulston et al. (2019)	3 U.S. mothers and their children with ASD	Not measured	Yes	Intervention Parent selected behavioral strategy use Parent self-reported stress, and Child challenging behavior	Medium effect for increases in behavioral strategy use and small-moderate effects for decreases in parent stress and child challenging behavior was found following intervention.
Roach et al. (2019)	15 U.S. parents and caregivers of young children	FFMQ-SH	Yes	Intervention Mindfulness Mental health Parenting competency	Participants reported increased mindfulness and decreased levels of anxiety and depression following intervention. Parent reports of family stressors were relatively stable across the two time points, while self-reported parenting competence decreased.
Yang et al. (2019)	123 China pregnant women	FFMQ	Yes	Intervention Depression Anxiety Mindfulness	Women in the intervention group showed greater decline in depression and anxiety, and significant increase in mindfulness compared to those in the control group.
Barrio Martinez et al. (2020)	37 Spain parents of children in secondary education	MAAS	Yes	Intervention Mindfulness Interpersonal reactivity Parents’ satisfaction with parenting abilities and parent-child relationship	Parents in intervention reported significant increase in mindfulness than parents in control group. Parents also reported significant increase in their satisfaction in their parenting abilities and parent-child relationship following intervention.
Boekhorst et al. (2020)	157 Netherland mothers with toddlers	IM-P	Yes	Intervention acceptability Parental stress, Over reactivity in parenting Self-compassion Anxiety Depression	Mindful parenting is acceptable and effective for women without elevated levels of stress. Parents reported significant improvement in selfcompassion, parental over reactivity, and symptoms of anxiety and depression at follow-up.
Calvete et al. (2020)	348 Spain parents and their adolescent	MIPQ FFMQ-SF	No	Mindful parenting Child mindfulness Peer aggressive behavior and victimization Depression	Mindful parenting predicted reduced depressive symptoms, aggression, and victimization after 1 year. Adolescents with poor dispositional mindfulness benefited more from mindful parenting.
Dieleman et al. (2020)	58 Belgium parents of children with cerebral palsy	BMPS	No	Mindful parenting Day-to-day variation in parents’ psychological needs and child behavior Day-to-day variation in parents’ Autonomy-support Psychological control Responsive parenting behavior	Daily fluctuations in both child behavior and parents’ own psychological needs are associated with daily variability in parenting. Interindividual differences in mindful parenting are positively associated with daily variability in parenting.
Elgendy et al. (2020)	100 Egypt parents of children with ADHD	IM-P	No	Mindful parenting Parental stress	Mindfulness is significantly negatively correlated with parental stress.
Evans et al. (2020)	225 Australia families (120 families with child ADHD and 105 control families)	IM-P	No	ADHD, mindful parenting Parenting behaviors Psychological distress Children’s self-regulation	Parents of children with ADHD report significantly lower mindful parenting than parents in control group. Higher mindful parenting was associated with lower levels of parent psychological distress, higher levels of parenting warmth and consistently, lower levels of parenting anger, and higher child emotion self-regulation in both groups. Mindful parenting was indirectly associated with child emotion self-regulation through lower parenting anger.
Fernandes et al. (2020a)	560 Portugal mothers with a child 12 months old or younger	IM-P	No	Parent anxiety Depression Parenting stress Infant temperament Mindful parenting	Mothers who perceived their infant temperament as difficult had significantly higher levels of parenting stress and lower levels of mindful parenting than those who perceived their infant temperament as easier. Parenting stress mediated the relationship between anxious and depressive symptomatology and mothers’ perception of infant temperament and mindful parenting.
Fernandes et al. (2020b)	599 Portugal mothers with child aged 0–12 months	Not measured	Yes	Usefulness of mindful parenting intervention Knowledge and acceptability of mindful parenting interventions Preference concerning the characteristics of mindful parenting intervention	Approximately 95% of mothers felt that participating in a mindful parenting intervention during the postpartum period would be useful. Concerning mothers’ preferences, most mothers preferred a weekly frequency (85.0%) and an average of 10 sessions (48.6%) of 45–60 min in length (52.6%). Learning how to better understand the baby’s emotions and behaviours and learning new tools to better cope with parenting stress were among the intervention contents considered most useful.
Ljubetić and Ercegovac (2020)	101 Croatia two-parent families with an adolescent child	MIPQ	No	Mindful parenting Cognitive parental awareness and Adolescents’ psychological well-being	Mindful parenting and cognitive parental awareness are significantly correlated to the subjective wellbeing of adolescents when it comes to fathers, but not to mothers.
Maglica et al. (2020)	168 Croatia participants (76 mothers, 76 fathers, 16 teachers) and their children (n=76)	MIPQ	No	Children’s internalizing and externalizing problems Mindful parenting	Mindful parenting did not predict children’s internalizing problem. Fathers’ focusing attention on the child with acceptance and mothers’ self-efficacy were related to lower externalizing problems. Fathers’ empathic understanding of the child and mothers’ non-reactivity were related to more externalizing problems.
Mohammadi et al. (2020)	72 Iran mothers with blind girls	Not measured	No	Mindful parenting Psychological capital Parental stress Psychological flexibility	Mothers in the intervention group reported decrease in stress and increase in psychological flexibility compared to mothers in the control group.
Moreira and Canavarro (2020)	375 Portugal mother-adolescent dyads	IM-P	No	Adolescents’ difficulties in emotion regulation Mindful parenting	The mindful parenting dimensions of compassion for the child and nonjudgmental acceptance of parental functioning were indirectly associated with difficulties in emotion regulation through self-compassion. The mindful parenting dimension of listening with full attention was indirectly associated with difficulties in emotion regulation through psychological inflexibility.
Moreira et al. (2020)	399 Portugal parents of children aged 6–13	IM-P	No	Parents’ overprotection and supportive behaviors Parents’ and children’s tendency to experience negative affect Mindful parenting	Parents’ and children’s tendency to experience negative affect were associated with lower levels of all mindful parenting. Parents’ neuroticism and children’s negative reactivity were both shown to be indirectly associated with lower levels of overprotection and supportive behaviors through lower levels of compassion towards the child and of emotional awareness of the child. In contrast, parents’ neuroticism and children’s negative reactivity were indirectly associated with a greater overprotection through lower levels of nonjudgmental acceptance of parental functioning.
Nguyen et al. (2020)	522 mothers	IM-P	No	Self-compassion Gratitude Mindful parenting	Self-compassion is indirectly associated with greater mindful parenting through gratitude.
Parent et al. (2020)	564 U.S. parents of children aged 3–17	MAAS IM-P	No	Caregiver dispositional mindfulness Mindful parenting Parenting behaviors Youth internalizing Externalizing problems	Higher levels of baseline caregiver dispositional mindful attention were related to higher levels of mindful parenting at 4 months. Higher levels of mindful parenting were associated with higher levels of positive parenting and lower levels of negative parenting practices at 8 months. Lower levels of negative parenting practices were related to lower levels of internalizing and externalizing symptoms at 12 months.
Potharst et al. (2020)	73 Netherland mothers	IM-P	No	Video-observations of parent-child interactions Self-reported mindful parenting	The IM-P total score is predictive of maternal actual attention for the child during a face-to-face interaction.
Pugsley and Acar (2020)	1324 parents	IM-P	No	Parents’ perception of creative and socially acceptive characteristics in children Parents’ attitudes and values toward creativity Creative home environment Mindful parenting	Parents’ attitudes and values toward creativity and creative home environment were significantly and positively related to support for creativity characteristics, whereas mindful parenting was significantly and negatively related to support for socially acceptable characteristics in children.
Ren et al. (2020b)	334 China parents (167 parents of children with ASD and 167 parents of typically developing parents)	IM-P	No	Positive and negative parenting practices Mindful parenting	Compared to parents of typically developing children, parents of children with ASD showed less listening with full attention, less proactive parenting, less supportiveness, more lax control, and more physical control to their children. Listening with full attention and awareness of children’s emotions were significantly related to both positive and negative parenting practices in families of children ASD.
Wang and Lo (2020)	201 parents	IM-P	No	Child and parent mental health Mindful parenting	Significant correlations were found between mindful parenting, parental stress, child behavior problems, and social support from family. Nonjudgmental acceptance of the child was a significant moderator of the relationship between parental stress and child behavior problems.
Burke et al. (2020)	1007 U.S. parents with youngest child under age of 19	FFMQ	No	Parenting efficacy parenting stress	Nonreactivity of inner experience was most predictive of parenting efficacy compared to acting with awareness, whereas awareness was most predictive of lower parenting stress compared to nonreactivity.
Fereydooni et al. (2020)	45 Iran mothers	Not measured	Yes	Intervention Parenting self-efficacy Children’s anxiety	Mindfulness intervention was effective in promoting parenting self-efficacy, and the effect persisted over time.
Gheibi et al. (2020)	40 Iran women btw 16–28 weeks gestation	Not measured	Yes	Intervention Maternal fetal attachment	Maternal-fetal attachment was significantly higher in the intervention group.
Guo et al. (2020)	284 China pregnant women	MAAS	Yes	Intervention Postpartum depression and anxiety	Women in the intervention group showed significant improvement in depression and anxiety compared to the control group.
Kil and Grusec (2020)	127 Canada mothers	FFMQ	No	Maternal stress Mothers’ perspective taking Adolescent disclosure to mothers Parent-child conflict intensity	Mothers’ greater dispositional mindfulness was associated with adolescent reports of greater maternal perspective-taking through less maternal stress. Mothers’ greater dispositional mindfulness was associated with more adolescent disclosure to mothers and less intense conflict through less maternal stress and greater maternal perspectivetaking
Lo et al. (2020)	100 Hong Kong parents of children with ADHD	IM-P	Yes	Intervention Child ADHD symptoms Parenting stress Well-being	Families in the intervention group had greater improvements in children’s ADHD symptoms, parenting stress, and well-being.
Lönnberg et al. (2020a)	193 Sweden first-time pregnant women	FFMQ	Yes	Intervention Perceived stress Depressive symptoms Positive state of mind Mindfulness	Compared to the active control treatment, MBCP significantly reduced perceived stress, depressive symptoms, increased positive state of mind, and mindfulness. Change in mindfulness mediated the treatment effects of MBCP on stress, depression symptoms, and positive state of mind.
Lönnberg et al. (2020b)	193 Sweden first-time pregnant women	FFMQ	Yes	Intervention Stress Depression Positive state mind Mindfulness	Mothers in the intervention group had a greater decrease in stress and depression, and greater increase in positive state of mind and mindfulness from baseline to post-intervention, compared to active control group.
Mah et al. (2020)	63 Canada parents of children with ADHD	IM-P	Yes	Intervention Mindful parenting Parenting stress Harsh discipline practice Behavioral dysregulation Child ADHD symptoms	Parents in the mindful group had decreased harsh discipline practices and improved self-regulation compared to parents in the standard group. Both groups improved in parenting sense of competence and child ADHD symptoms. No significant group differences were found in mindful parenting or parenting stress.
McGregor et al. (2020)	80 U.S. children with DD and ASD and their parents	BMPS	Yes	Intervention Parent stress Child internalizing problem	Children of parents in the MBSR treatment group had greater reductions in internalizing problems compared to children whose parents were in the control group. Children of parents who reported greater increase in mindfulness had greater reduction in internalizing problems.
Poormirzaei and Bagheri (2020)	278 Iran primary school children and their parents	IM-P	No	Child’s cognitive emotion regulation Mind reading Parent mindfulness	Maternal mindfulness is directly related to mind reading abilities of elementary school children. The effect of maternal mindfulness on the child’s mind reading ability was mediated by the child’s positive cognitive emotion regulation.
Ren et al. (2020a)	1723 China biological mothers	FFMQ	No	Perceived stress positive parenting practice Children’s emotion regulation	Higher level of maternal perceived life stress weakened the positive links between maternal mindfulness and positive parenting practices and between maternal mindfulness and school-aged children’s emotion regulation.
Rice et al. (2020)	23 Ireland parents of children with ADHD	MAAS	Yes	Intervention Parental stress Parenting competence Quality of life Mindfulness Child hyperactivity	Significant improvement in quality of life and reduction on the child hyperactivity was found following the intervention.
Roberts et al. (2020)	47 U.S. parents of children with DD	Not measured	Yes	Intervention Parenting daily hassles Salivary cortisol	Both self-reported parenting stress and cortisol awakening response decreased following MBSR for parents of children with DDs.
Shaffer et al. (2020)	11 U.S. parents of children with special needs	FFMQ-SF	Yes	Intervention Perceived stress Depression Anxiety Mindfulness	Parents reported significant reduction in stress and increase in mindfulness following intervention.
Singh et al. (2020)	195 U.S. mothers of children with ASD	Not measured	Yes	Intervention Perceived stress Child aggressive behavior Disruptive behavior Compliance with mother’s request	Mothers in the MBPBS condition reported greater reductions in perceived psychological stress, followed by those in the mindfulness condition (MB), and with no significant changes reported by those in the positive behavior support condition (PBS). Significant increases in compliance were largest in the MBPBS condition, followed by mindfulness condition (MB), and then positive behavior support condition (PBS).
Van Gampelaere et al. (2020)	33 U.S. families (51 parents) with children diagnosed with type 1 diabetes	MAAS	No	Diabetes-specific parent-child interaction Parental stress parental state anxiety parental trait mindfulness	Parental stress and anxiety were related to more maladaptive and less adaptive parent-child interactions. For mothers, mindfulness was related to less observed discomfort of the child during injection. For fathers, more emotional involvement was related to better child glycemic control.
Wang et al. (2020)	2237 China parents of school-aged children	FFMQ	No	Parent’s mental health Dispositional mindfulness Family risk	Dispositional mindfulness moderated the relationship between parenting-related risks and parental mental health, such that the negative impact of parenting-related risks was attenuated for parents with high dispositional mindfulness.
Weitlauf et al. (2020)	61 U.S. parents of children with ASD	FFMQ	Yes	Intervention Depressive symptoms Anxiety symptoms Parental distress Life satisfaction Parent-child dysfunctional interaction	Parents who received MBSR had greater improvements than those receiving P-ESDM only in parental distress and parent-child dysfunctional interactions.

*ADHD* Attention-Deficit/Hyperactivity Disorder, *ASD* Autism Spectrum Disorder, *BMPS* Bangor Mindful Parenting Scale, *CAMM* Children’s Acceptance and Mindfulness Measure, *CAMS-R* Cognitive and Affective Mindfulness Scale-Revised, *DD* Developmental Disabilities, *FFMQ* Five Facet Mindfulness Questionnaire, *FFMQ-SF* Five Facet Mindfulness Questionnaire-Short Form, *FMI* Freiburg Mindfulness Inventory, *ID* Intellectual Disability, *IDD* Intellectual and Developmental Disabilities, *IME-P* Interpersonal Mindfulness in Parenting, *IM-P* The Interpersonal Mindfulness in Parenting scale, *IM-P-I* Interpersonal Mindfulness in Parenting - Infant Version, *MAAS* Mindful Attention Awareness Scale, *MAIA* The Multidimensional Assessment of Interoceptive Awareness, *MBCP* Mindfulness-Based Childbirth and Parenting, *MBCT* Mindfulness-based Cognitive Therapy, *MBI* Mindfulness-Based Intervention, *MBSR* Mindfulness-based Stress Reduction, *MIPQ* Mindfulness in Parenting Questionnaire, *MP* Mindful Parenting, *MPE* Mindful Parenting Education, *MSFP* The Mindfulness-enhanced Strengthening Families Program, *SUUM* Subjective Units of Use of Mindfulness, *TMS* Toronto Mindfulness Scale

**Table 2:** Content Analysis of Parental Reflective Functioning Articles (*N* = 121)

Study	Sample	RF Measure	RCT Design (yes/no)	Other Variable(s)	Results

Grienenberger et al. (2005)	45 mother-infant dyads (94% White) (United States)	PDI	No	Disrupted affective communication (OB) Infant attachment (OB)	Negative correlation between RF (10 mos.) and disrupted communication (14 mos.) Higher RF predicted infant attachment Maternal behavior partially mediated the relationship between RF and attachment
Schechter et al. (2005)	41 mothers (88% Hispanic) (United States)	WMCI	No	Maternal PTSD (SR) Mothers’ representations of children (INT)	RF & PTSD unrelated Mothers with higher RF more likely to have balanced representations of child
Slade et al. (2005a, b)	40 mother-infant dyads (94% White) (United States)	PDI	No	Maternal attachment (INT) Infant attachment (OB)	Autonomous mothers had higher RF scores Dismissing & preoccupied mothers had higher RF than unresolved mothers Secure infants had mothers with higher RF than preoccupied or disorganized
Schechter et al. (2008)	41 mother-infant dyads (United States)	WMCI	No	Atypical maternal behavior (OB)	RF and atypical maternal behavior unrelated
Pajulo et al. (2008)	18 mother-infant dyads (Finland)	PI PDI	No	Intervention Maternal sensitivity	60% of the mothers reported increase in RF following intervention (residential treatment for substance abuse) Positive association between prenatal RF and maternal sensitivity at 4-months
Rosenblum et al. (2008)	95 mother-infant dyads (United States)	WMCI	No	Mind-minded comments Maternal sensitivity Maternal depression symptoms	RF positively associated with mind-minded comments & maternal sensitivity RF negatively related to depression symptoms
Suchman et al. (2008)	14 mothers in substance use treatment (70% White) (United States)	PDI	No	Intervention (Mothers & Toddlers Program) Maternal representational balance	Increase in RF following 12-week treatment RF partially mediated link between maternal representational balance and maternal behavior
Suchman et al. (2010)	47 mothers in substance use treatment (70% White) (United States)	PDI	Yes	Intervention	Higher post-treatment RF for intervention condition
Suchman et al. (2010)	47 mothers in substance use treatment (10% White) (United States)	PDI	Yes	Maternal Sensitivity	Confirmed two-factor structure of RF: Self-focused & child-focused Self-focused RF positively associated with sensitivity Child-focused RF unrelated to sensitivity
Borelli, West, DeCoste and Such- man et al. (2011)	47 mothers in substance use treatment (70% White) (United States)	PDI	Yes	Emotion word use Maternal Sensitivity	Positive emotion words associated with lower self-focused RF Positive feeling words partially mediated links between RF and maternal sensitivity
Ha et al. (2011)	652 mother-child dyads (91% White) (United Kingdom)	Social scenarios (distorted mentalizing task)	No	Child conduct problems (self-reported)	Maternal mentalizing and mother-reported child conduct problems negatively correlated at baseline and follow up even after controlling for baseline conduct problems. Maternal mentalizing unrelated to child-or teacher-reported conduct problems
Suchman et al. (2011)	47 mothers in substance use treatment (10 White) (United States)	PDI	Yes	Intervention (Mothers & Toddlers Program)	Higher RF in treatment group than control group at post-treatment and 6-week follow up No differences in child-focused RF
Benbassat and Priel (2012)	105 adolescents and their parents (Israel)	PDI	No	Adolescent (A) RF Internalizing problems Externalizing problems A social competence A self-perception	Both mothers’ and fathers’ RF associated with adolescent RF Fathers’ RF was positively associated with adolescent social competence, internalizing problems and negatively associated with adolescent self-perception Parental RF moderated links between parenting behavior (e.g., involvement, warmth) and adolescent outcomes
Pajulo et al. (2012)	34 mothers in residential treatment for substance use (Finland)	PI PDI	No	Intervention	RF increased from prenatal to postnatal phase for 63% of mothers Smaller increases in RF for those who also used alcohol, exposed to physical abuse and secrets within family during childhood, experienced secrets or abuse/ neglect Education positively associated with postnatal RF
Suchman et al. (2012)	24 U.S. mothers in substance use treatment (71% White)	PDI	No	Overall maternal representational quality of child (RQ) Treatment fidelity	Greater adherence to treatment components resulted in greater improvement in RQ Greater improvement in RQ explained unique variance in improvement of caregiving behavior (e.g., sensitivity to cues and responses to distress) Improvement in overall RQ partially mediated association between treatment fidelity and caregiving behavior
Esbjørn et al. (2013)	38 Denmark clinically anxious children & their parents (100% Danish)	AAI	No	child anxiety (SR)	Mothers had higher RF than fathers Higher mother RF x lower father attachment avoidance predicted lower child anxiety
Rutherford et al. (2013)	21 mothers (43% White, 19% African American, 10% Hispanic) (United States) (United States)	PRLQ	No	Maternal distress tolerance (OB)	RF (interest & curiosity subscale) associated with more persistence in soothing distressed infant
Sadler et al. (2013)	105 young mothers (28% Black, 62% Latina)	PI PDI	Yes	Intervention (Minding the Baby)	RF improved over time in both treatment & control conditions No time x group effects, except in subgroup of parents with very low RF
Sleed et al. (2013)	163 mother-infant dyads residing in prison (51% White, 32% Black) (United Kingdom)	PDI	Yes	Intervention (New Beginnings)	Significant time x group interaction: RF increased from baseline to posttreatment for intervention relative to control Control group RF declined over time
Huth-Bocks et al. (2014)	115 mother-infant dyads (75% White) (United States)	PDI-SF	No	Secure base scripts (narrative)	Secure base script scores positively related to RF
Ordway et al. (2014)	50 high-risk mother-child dyads (70% Latina) (United States)	PI PDI	Yes	Intervention (Minding the Baby)	Group unrelated to RF; No group x time interaction predicting RF Education positively correlated with RF
Stacks et al. (2014)	83 mother-infant dyads with or without a history of child maltreatment (United States)	PDI-SF	No	Maternal RF Maternal parenting negativity and sensitivity Infant attachment security Maternal depression symptoms and PTSD	RF positively correlated with depression symptoms and maternal sensitivity RF negatively correlated with parenting negativity and demographic risk Mothers of secure children had significantly higher RF Both parenting sensitivity and negativity mediated association between RF and infant attachment security. RF unrelated to mothers’ childhood maltreatment or PTSD
Stover and Kiselica (2014)	79 fathers (56% African American) (United States)	PDI	No	Parent-child attachment Parental discipline (consistently applying consequences to misbehaviors) Hostile-aggressive parenting (SR)	RF negatively correlated with drug use frequency and positively correlated with education RF unrelated to parent-child attachment or hostile-aggressive parenting RF positively correlated with parental consistent disciplining practices, not significant after controlling for covariates
Bammens et al. (2015)	30 adoptive/foster parents (United States)	FMSS	No	Intervention (Family Minds)	RF scores significantly increased for intervention group but not comparison group
Berthelot et al. (2015)	57 mother-infant dyads (78% Caucasian) (Canada)	AAI	No	Infant attachment disorganization (OB)	RF regarding trauma and unresolved trauma together predict disorganized attachment Global RF does not predict disorganized attachment
Bunday et al. (2015)	12 foster parents (United States)	PDI	No	RF	RF scores ranged from 3 to 7 with a variety of scores across sample and across child and self-focused RF
Ensink et al. (2015)	94 mother-child dyads (Majority White) (United States)	PDI	No	Child RF (INT)	Positive correlation between children’s RF and maternal RF; not significant after controlling for child sexual abuse
Huber et al. (2015)	83 caregiver-child dyads (29% diverse background) (Australia)	COS Interview	No	Intervention (Circle of Security)	Lower RF among caregivers with history of family violence, divorced or separated caregivers, caregivers of older children, caregivers with less education, caregivers of boys RF increased post-intervention for caregivers with baseline RF lower than 5 and those with less than postsecondary education
Huber et al. (2015)	83 caregiver-child dyads (29% diverse background) (Australia)	COS Interview	No	Internalizing problems Externalizing problems Protective factors	RF unrelated to protective factors and behavior problems
León et al. (2015)	98 adoptive and non-adoptive families (Spain)	PDI-SF	No	Parenting stress Children psychological problems	RF negatively correlated with parenting stress in adoptive families Global RF negatively correlated with children’s behavior problems, hyperactivity problems, and total problems in adoptive families
Paris et al. (2015)	66 mother-child dyads in residential treatment for substance use (79% White) (United States)	PRFQ	No	Intervention Psychological distress (SR)	Paranoid ideation negatively associated with RF interest/curiosity and positively with RF prementalizing Social-emotional risk negatively associated with RF certainty of mental states Mothers in top third of psychological distress demonstrated positive changes in RF interest & curiosity over time
Rosso et al. (2015)	41 mother-child dyads (100% Italian) (Italy)	AAI	No	Children’s mental-state talk and mentalization Maternal RF	Maternal RF higher in secure than insecure mothers Positive associations between overall maternal RF and both children’s mentalization and mental-state talk (except emotional lexicon)
Rutherford et al. (2015)	62 mothers (53% African American) (United States)	PRFQ	No	Maternal distress tolerance (SR) Parental distress tolerance (OBS via baby simulator task)	RF certainty negatively correlated with maternal age and education RF pre-mentalizing negatively correlated with maternal distress tolerance (SR) RF pre-mentalizing negatively associated with task persistence RF interest & curiosity positively associated with lower systolic BP before, during, and after task; not significant after controlling for age & education
Scopesi et al. (2015)	41 mother-child dyads (100% Italian) (Italy)	AAI	No	Children’s mental state terms	Maternal RF significantly predicted children’s use of mental state terms
Smaling et al. (2015)	162 pregnant women (85% White) (Netherlands)	PI	No	Risk factors Risk group (high vs. low-risk)	Prenatal RF significantly lower in high-risk group In high-risk group, number of risk factors negatively related to prenatal RF Education, social support and substance use during pregnancy were significant predictors of prenatal RF
Ashton et al. (2016)	51 caregiver/child dyads (Canada)	PRFQ	No	Caregiver-child attachment PRF Child well-being Parent-child relationship quality	At post-assessments: attachment, parental RF, and relationship quality improved (communication, involvement, relationship frustration)
Borelli et al. (2016)	117 parent-child dyads (38% Latino/a; 36% White) (United States)	PDI-R	No	Parent and child RF (RF divided into two dimensions--parent RF and child RF)	Confirmed factor structured of RF for a community sample of parents of school-age children No significant differences between PRF across certain demographic variables (e.g., parent and child sex). Married parents had higher PRF Non-significant associations between parent attachment and child and parent RF Child attachment was significantly associated with PRF
Claydon et al. (2016)	59 mothers (73.7% White) (United States)	PRFQ	No	PRF	Mothers with eating disorders (ED) had higher PRF than non-ED mother
Ensink et al. (2016)	168 mother-child dyads (98% White) (Canada)	PDI	No	Parent RF Child RF Child psychopathology (depression and externalizing difficulties)	Child RF significantly mediated the association between childhood sexual abuse and child psychopathology Maternal RF was associated with child externalizing difficulties
Ensink et al. (2016)	88 mother-child dyads (100% White) (Canada)	AAI	No	Parenting behaviors and infant attachment (observation)	RF significantly associated with greater sensitivity, less negative parenting, and child attachment security RF had significant indirect effects on child attachment via maternal insensitivity
Fonagy et al. (2016b)	76 mother-child dyads (Majority White) (United States)	PDI	Yes	Infant development. Parent-infant interaction Maternal psychopathology Maternal representations Maternal RF Infant attachment	No differences between groups on measures of parent-infant interactions No statistically significant differences in child attachment or PRF post-program. Intervention groups reported greater improvement on maternal psychopathology, parenting stress, and maternal representations of child
Heron-Delaney et al. (2016)	33 mother-infant pairs (Australia)	PDI	No	Infant emotion regulation	Statistically significant differences in infant negative affect behaviors (cry- ing/fussing) between infants of mothers with high versus low RF. Infants with high maternal RF demonstrated the highest level of negative affect at the still face episode versus the reunion episode for infants with low maternal RF.
Hertzmann et al. (2016)	15 co-parents dyads (30 total) (United Kingdom)	PDI and PRFQ	Yes	PRF Co-parenting anger Parenting alliance and hostility Parental stress and depression Child behaviors	No significant differences between control and treatment on reflective functioning across PDI and PRFQ Significant changes in parenting stress/depression, parenting alliance and hostility, and child behaviors at postassessments but no significant effects of intervention
Kohlhoff et al. (2016)	15 mothers (Australia)	PRFQ	No	Maternal RF, caregiving helplessness, feelings about their child (postpartum bonding), stress	Significant increase in PRF-Certainty subscale, postpartum bonding, but decreases in caregiving helplessness, mother and child frightened, and stress at post-COS-P
Mohaupt and Duckert (2016)	36 fathers who committed intimate partner violence (Norway)	PDI-R	No	Paternal RF Substance and alcohol use Trauma Parental stress	PRF was not significantly correlated with substance/alcohol use, lifetime trauma, or childhood trauma Childhood trauma (physical abuse) was significantly correlated with PRF, drug use, and lifetime trauma
Rosso and Airaldi (2016)	39 mother-preadolescent dyads (Italy)	AAI	No	Maternal RF Child attachment security	Child attachment was significantly associated with child RF Children with higher RF were more likely to have mothers with secure attachment
Rostad and Whitaker (2016)	79 parents (85.5% European American) (United States)	PRFQ	No	Parent-child relationship quality Child attachment security	RF was significantly associated with quality of parent-child relationship including parental involvement, communication, parent satisfaction, limit setting, and parental support Parent-child relationship quality (support, limit setting, autonomy) was correlated with attachment anxiety Parental support, satisfaction with parenting, involvement, and communication and attachment avoidance were associated with attachment avoidance
Sealy and Glovinsky (2016)	40 parent-child dyads (children w/neurodevelopmental disabilities) (Caribbean)	PDI	Yes	PRF	Treatment parents had higher PRF than parents in the control group
Smaling et al. (2016)	123 mother-infant dyads (89% Caucasian) (Netherlands)	PI - R PDI	No	Child temperament Child externalizing behaviors	Prenatal RF was associated with child physical aggression
Smaling et al. (2016)	133 mother-infant dyads (89% Caucasian) (Netherlands)	PI - R	No	Postnatal maternal interactive behavior	Prenatal RF was associated with accumulated risk (intrusiveness, and internalizing-helplessness) Higher maternal RF was associated with positive reengagement
Stover and Coates (2016)	24 fathers with IPV and substance use problems (54.2% African American) (United States)	PDI-R	No	Parenting behaviors (e., adult criticizing, child avoidance, and dyadic tension) Intimate partner violence (IPV)	IPV (male to partner physical aggression) was significantly correlated with parenting behaviors (dyadic constriction) and child avoidance of parents during parent-child interactions PRF was not significantly correlated with any measured variables
Suchman et al. (2016)	17 mothers from outpatient mental health clinic (44.4% White, 33.3 % Hispanic or Latina) (United States)	PDI	No	Feasibility of MIO program Change in quality of mother-child interactions (RF and parenting stress)	MIO had good acceptability and feasibility with 83% completion. Child-focused RF increased after intervention but self-focused RF did not. Mothers reported decreases on certain parenting stress subscales (Personal Distress and Difficult Child) but not Parent-child Dysfunctional at postassessments
Alvarez-Monjarás et al. (2019)	142 mothers in substance use treatment (64.2% White) (United States)	PDI	No	Maternal RF and caregiving	Maternal RF was significantly associated with caregiving (sensitivity) and quality of mental representation of the child Maternal RF significantly mediated the association between quality of mental representation of child and maternal sensitivity
Burkhart et al. (2017)	300 parents (65.3% White) (United States)	PRFQ	No	PRF (pre-mentalizing) Parenting satisfaction Positive emotions positivity	Relational savoring group had higher positivity scores than personal savoring group, but there were no significant group differences The indirect effects of attachment anxiety on parenting satisfaction, and positivity was present via PRF (prementalizing) in the single-group model analysis. Attachment anxiety had a significant indirect effect on relationship satisfaction, positivity, and positive emotions for the relationship savoring group via RF. For the personal savoring group, RF only significantly mediated the association between attachment anxiety and relationship satisfaction
Cooke et al. (2017)	240 (120 couples; 12.9% Australian) (Australia)	PRFQ	No	Maternal and Paternal family functioning Parenting efficacy Fathering role	Fathers scored significantly higher on two RF subscales (Prementalizing and Interest in Child) than mothers. There were no differences between mothers and fathers on the Certainty of Mental States subscale. Both mothers’ and fathers’ own RF (Certainty of Mental States) were significantly associated with their own Prementalizing
Cordes et al. (2017)	79 mothers (postpartum depression and non-clinical group) (Denmark)	AAI	No	Postnatal depression Personality disorder	No significant associations between RF and depression and personality disorder No significant differences between clinical and nonclinical groups on RF scores
Ensink, Bégin, et al. (2017a)	154 mother-child dyads (64 children had experienced childhood sexual abuse [CSA]) (Canada)	PDI	No	Maternal RF Child behaviors	Maternal RF was negatively correlated with child internalizing and externalizing difficulties Mothers in the non-CSA groups reported higher RF than the CSA group Maternal RF significantly moderated the association between CSA and child internalizing difficulties
Ensink et al. (2017a, c)	86 mother-child dyads (Canada)	AAI	No	Personal characteristic (RF) and personality organization (identity diffusion, reality testing, and primitive defenses)	Mothers with significantly lower RF and personality organization reported intrusive/aggressive parenting behaviors Maternal withdrawal and disconnection was associated with RF and personality organization
Maupin et al. (2017)	131 mothers (44% Hispanic) United States	PRFQ	No	Maternal depression, efficacy, and competency, maternal RF and parent- child relationship	Mothers reported significant decrease in depressive symptoms at postassessments No significant improvements on maternal RF, parental efhcacy/competency, or parent-child relationship
Möller et al. (2017)	40 mother-child dyads (Sweden)	PDI-R RF-limiting setting (RF-LS)	No	RF on PDI and emotional availability (sensitivity, structuring, non-intrusive- ness, non-hostility, child responsiveness/involve- ment)	RF-FS was significantly associated with RF-PDI. RF-FS was significantly correlated with all emotional availability subscales except non-hostility RF-PDI was significantly correlated with three emotional availability subscales (sensitivity, non-intrusiveness, and child responsiveness)
Rutherford et al. (2017)	62 U.S. mothers (49.2% White)	PRFQ	No	neural correlates of infant face/cry perception using event-related potentials (ERPs)	Maternal RF was associated with neural correlates of infant cue perception
Cristobal et al. (2017)	124 mothers (Chile)	PRFQ	No	PRF	Insecure attachment and trauma (emotional negligence) was significantly correlated with RF (pre-mentalization). Secure attachment was significantly associated with emotional negligence Mothers with insecure attachment and reported more physical neglect in childhood (trauma) were more likely to experience lower reflective functioning
Shai et al. (2017)	68 mother-infant dyads (Israel)	PDI-SF PEM	No	PDI-RF and parental (PEM) Parental stress -coparental alliance Infant temperament	PEM-RF PDI-RF were significantly correlated PEM-RF was significantly associated with coparental alliance PDI-RF was not significantly associated with any other study variables PEM-RF has significantly indirect effects on parental stress via coparental alliance
Shai and Belsky (2017)	200 mother-infant dyads (United States)	PEM	No	Infant-child cognitive and socio-emotional functioning Maternal behaviors and stress	PEM was inversely associated positively associated with maternal stress and sensitivity and child outcomes including internalizing/externalizing behaviors but positive correlated with child language, academic, social skills, and competence
Smaling et al. (2017)	96 mother-infant dyads (84.8% White) (Netherlands)	PI - R	No	Maternal behavior Infant aggression	Prenatal RF was significantly associated with Time 2–4 infant aggression, and Time 2 maternal behavior (Time 2 sensitivity) Mothers who were in the non-intrusive group and had low RF had infants who reported significantly more infant aggression, than high RF mothers
Suchman et al. (2017)	87 U.S. mother-child dyads; mothers enrolled in substance abuse treatment (77% White)	PDI	Yes	Maternal addiction severity Maternal intelligence, Maternal representation of child PRF Maternal psychiatric symptoms Maternal substance use Child attachment	MIO mothers reported higher RF at post- and 3-month assessments than PE mothers PE showed lower psychiatric symptoms at post-assessments but at the 3-month follow-up both PE and MIO showed normative levels of psychiatric distress and depression There was no significant difference in substance use across time MIO children were marginally more engaging with mothers than PE group at post-treatment. At 12-month MIO show significantly more engagement/involvement and dyadic reciprocity No significant differences in child attachment post-program
Wong et al. (2017)	84 U.S. mother-infant dyads (64.2% White)	PDI-R	No	RF Infant negative affect Toddler behavior	There was a significant correlation between infant negative affect and toddler behavior problems, both were significantly associated with cumulative risk Average and low levels of RF significantly moderated the association between infant negative affect and toddler behavior problems
Adkins et al. (2018)	102 U.S. foster parents (61% White)	PRFQ FMSS-RF	No	RF Parenting stress	FM group had significantly higher RF (Certainty and Curiosity) at post-test FM group had increased in RF (FMSS-RF) at post-test than the parenting class group Parenting stress decreased for the FM group. There were group differences at post-test on the Defensive Responding subscale, with the parenting class group reporting higher scores on this subscale
Byrne et al. (2019)	16 parents at risk for disorganized attachment (88% White-British) (United Kingdom)	PDI	No	Global distress Parental sensitivity Parenting self-efficacy Parent well-being	At post-treatment, parents reported increased sensitivity and reduction in parenting stress but the changes were not statistically significant. RF, depression, and anxiety did not improve at the end of the program
Riva et al. (2018)	44 adolescent mother-infant dyads 41 adults mother-infant dyads (Italy)	AAI	No	Maternal attachment PRF Maternal mind-mindedness Maternal emotional availability and sensitivity	More adult mothers had secure attachment Adolescent mothers had lower mind- mindedness and emotional availability than adult mothers
Håkansson et al. (2018)	43 mothers with substance use disorders (Norway)	PDI-R	No	Psychological well-being Trauma Executive function	RF was inversely associated with early childhood adversity, emotional/physical/sexual abuse, and neglect but was positively associated with early childhood and latency adaptive, competence, EF and safety
Håkansson et al. (2018)	43 mothers with substance use disorders (Norway)	PDI	No	Executive function Mental health	RF was significantly associated with EF (working memory, cognitive flexibility/inhibition, planning, and verbal/non-verbal IQ) Statistically significant inverse association between RF and mental health
Jessee et al. (2018)	97 mother-father dyads (Majority White) (United States)	AAI	No	Marital and coparenting quality Child behavior	Wife RF was positively associated with marital engagement, supportive coparenting and inversely associated with marital conflict and undermining coparenting Husband RF was positively correlated with marital engagement only
Krink et al. (2018)	50 mother-infant dyads (mothers with PPD) (German)	PRFQ	No	Maternal sensitivity Maternal depression	Maternal RF-Prementalizing was inversely associated with change in maternal sensitivity but positively associated with depression
León et al. (2018)	98 (40 adoptive and 58 non-adoptive parents) (Spain)	PDI	No	Quality of parent-child interaction	RF (negative/angry) was inversely correlated with parent’s encouraging behavior RF (positive perception of child in relationship) was positively associated with parent’s encouraging behavior, sensitivity and dyad creativity, but inversely associated with child’s negative quality of demeanor Adoptive parents had higher positive components of RF than non-adoptive parents.
Nijssens et al. (2018)	76 couples (United States)	PRFQ	No	RF Parenting stress	RF (prementalizing) for postpartum mothers and fathers at Time 2 was significantly correlated with parenting stress Father RF (Certainty) at Time 2 was associated with attachment anxiety. RF (prementalizing) significantly mediated the association between attachment and parenting stress
Rutherford et al. (2018)	Study 1: 50 mothers (48% African American) Study 2: 68 U.S. mothers (50% African American) (United States)	PRFQ	No	PRF	Study 1–2: Maternal RF (Interest and Curiosity) was positively associated with working memory Study 2: Interest and Curiosity was positively associated with set-shifting
Rutherford et al. (2018)	35 U.S. mothers (45.7% White)	PRFQ	No	Postpartum RF	Mothers’ infant LPP during pregnancy predicted postpartum maternal RF
Suchman et al. (2018)	84 mothers in substance use treatment (78.6% White) (United States)	PDI	Yes	Maternal RF Maternal mental representation of caregiving Maternal psychiatric distress and substance use Child attachment	Fidelity of the MIO program predicted maternal RF Improvements in maternal RF and caregiving representation was associated with maternal sensitivity Improved in maternal sensitivity was associated with child attachment security at post-assessment.
Berthelot et al. (2019)	301 pregnant women and expecting fathers (95% White) (Canada)	RFQ	No	RF Psychopathology Parental attitudes	Childhood maltreatment had significant indirect effects on psychological symptoms via RF
Borelli et al. (2019)	111 mother-child dyads (children exposed to CSA and comparison group) (98% White) (Canada)	PDI	No	Maternal childhood sexual abuse (CSA) Maternal childhood exposure to trauma	CSA-mothers with high RF about her own abuse history had children who were less likely to be exposed to trauma
Buttitta et al. (2019)	77 father-toddler dyads (62% White) (United States)	PDI-R	No	Parenting behaviors Socioeconomic risk	Father’s RF (child-focused) was associated with socioemotional supportive behaviors and moderated the relationship between SES and fathers autonomy supportive behaviors
Campora et al. (2019)	51 mothers (Italy)	AAI	No	Maternal emotion regulation	Maternal RF was not significantly correlated with emotion regulation
Riva et al. (2019)	63 mother-adolescent dyads (Italy)	AAI	No	Maternal RF Parent-child interaction Mother emotional states Infant emotional states	Maternal RF was not significantly associated with childhood maltreatment RF did not differ between mothers with or without maltreatment history Cumulative maternal childhood maltreatment was significantly associated with infant’s (Infant Negative) and mother’s emotional states (Mother Negative) and on a dyadic level (Infant Positive-Mother Negative)
Enav et al. (2019)	64 parents of children with ASD (Majority White) (United States)	PDI	No	Parental RF Emotion regulation Parental beliefs Parenting self-efficacy Child behaviors	Intervention group reported greater RF, emotion regulation beliefs, and parental self-efficacy and a decrease in child behaviors at post-treatment
Ensink et al. (2019)	88 mother-infant dyads (100% White) (Canada)	AAI Mini-PRFI	No	Infant attachment Maternal sensitivity	Mini-PRFI was positively associated with AAI-RF. Maternal insensitivity was inversely correlated with Mini-PRFI Disorganized infant attachment was correlated with AAI-RF and Mini-PRFI
Håkansson et al.(2019)	43 mothers with SUD (Norway)	PDI-R	No	Maternal RF Maternal cognitive flexibility Parental stress	Maternal RF was inversely correlated with parental and psychological stress but positively correlated with executive function (working memory, inhibition, and cognitive flexibility) RF significantly mediated the association between executive function and stress (parental and psychological distress)
Handeland et al. (2019)	43 mothers with SUD (Norway)	PDI-R RFQ	No	Maternal RF	Maternal RF (PDI) was significantly correlated with uncertain RF (RFu) and not certain RF (RFc) on the RFQ measure.
Midgley et al. (2019)	28 foster parents (96.4% White) (United Kingdom)	PRFQ RFQ	No	Pre- and post PRF RF Parenting stress	Parenting stress decreased after program No statistically significant changes in PRF or RF after the program
Ruiz et al. (2019)	322 parents (Australia)	PDI	No	Pre-term and at-term children	Mothers displayed higher RF than fathers for both at term and preterm children
Schultheis et al. (2019)	97 mothers (49.5% African American) (United States)	PRFQ	No	Emotion regulation Emotion dysregulation	RF (pre-mentalizing) was inversely correlated with ER-reappraisal and positively correlated with ER-suppression Emotion dysregulation was significantly correlated with RF (pre-mentalizing)
Stacks et al. (2019)	16 mothers with children under court jurisdiction due to maltreatment (75 % African American) (United States)	PDI-SF	No	Parental RF Parental responsiveness	43.8% of parents exhibited increases in RF, emotional responsiveness, and positive affect at post-treatment.
Staines et al. (2019)	48 adoptive parents (93% White) (United Kingdom)	PRFQ	No	Parental RF and self-efficacy Child behaviors	Parental self-efficacy and RF increased whereas child behavior difficulties (conduct problems) decreased at posttreatment Child emotional distress and peer problems increased at post-assessments
Zimmer-Gembeck et al. (2019)	139 caregivers-child dyads (Australia)	PRFQ	No	PRF Emotion dysregulation Child internalizing/externalizing symptoms	Child internalizing/externalizing symptoms, parental RF (prementalizing), and emotion dysregulation decreased Cognitive reappraisal and positive parenting increased at post-treatment
Álvarez et al. (2019)	90 mother-child dyads (Chile)	Adult’s speech in a structured situation (Farkas et al. (2017)	No	PRF	Mothers improved in mentalization at 30 months No significant correlation between maternal mentalization with child temperament and family SES at 12 and 30 months
Byrne et al. (2019)	16 parent-child dyads (88% White) (United Kingdom)	PDI	No	Parental sensitivity Parental efficacy Parenting well-being	Significant improvements in parent sensitivity, stress, and self-efficacy at post-program, but no improvements in RF
Georg et al. (2019)	1 mother (Germany)	RFS	No	RF	Higher maternal RF post FPIP program
Moser et al. (2019)	48 mother-child dyads (72.9% White) (Switzerland)	WMCI	No	RF	Mothers with a history of physical abuse have lower RF than their counterparts Mothers’ brain activation was significantly associated with RF for non- abused mothers
Salo et al. (2019)	45 mothers (Finland)	PDI	Yes	Maternal RF Parenting Maternal depression	Mothers improved in parenting (e.g., maternal sensitivity), RF, and decreased in depression at post-intervention than the control group
Anderson and van Ee (2020)	10 mothers (Netherlands)	PDI	No	RF and maternal well-being	Mothers with a child born from sexual violence exhibited lower RF scores than their counterparts No significant difference in PTSD symptoms and depression across both groups
Arikan and Kumru (2020)	537 mother-child dyads (Turkey)	PRFQ	No	Child behavior	RF was inversely associated with child internalizing and externalizing behaviors There were significant inverse associations between maternal well-being (e.g., depression) and child behaviors
Barone and Carone (2020)	46 mothers (Italy)	AAI	No	RF Attachment	Child abuse and neglect was negatively associated with RF and attachment
Borelli et al., (2020a)	108 mothers (72% French-Canadian) (Canada)	AAI	No	Dyadic cohesion Maternal insensitivity	RF moderated association between parental rejection and 17-month dyadic cohesion with partners, and maternal unresponsiveness and controlling behavior at 5-months
Borelli et al., (2020c)	Study 1: 106 mother-child pairs Study 2: 72 mother-child pairs (Majority White in both studies) (United States)	Study 1: RF coded from mom interview about child Study 2: PDI-R-SC	No	Study 1: caregiving sensitivity Study 2: child attachment, emotion regulation, stress reactivity (cortisol)	Study 1: RF associated with parental empathy, accuracy in perceiving child’s negative emotion, and mothers’ supportive behavior Study 2: RF associated with parental empathy; RF and empathy associated with less stress reactivity (less increases in cortisol)
Borelli et al., (2020b)	151 mothers-toddler pairs (65% White) (United States)	PRFQ	No	Child emotion regulation (distress, coping)	RF (certainty of mental states) moderated association between child distress and mother-directed coping; and between toddler distress and aggression
Carlone and Milan (2020)	212 mother-child dyads (78% White) (United States)	PRFQ RFQ	No	Child well-being	Negative RF domains were inversely correlated with child well-being (internalizing and externalizing behaviors)
Dejko-Wańczyk et al. (2020)	39 mother-child dyads (Poland)	AAI	No	Child behaviors Quality of parent-child relationship	RF was inversely associated with child aggressive behaviors but significantly associated with mother’s positive perception of the parent-child relationship
Dieleman et al. (2020)	268 parent-child dyads (Belgium)	PRFQ	No	PRF Parenting behaviors (psychological control)	Maternal and paternal RF was significantly associated with parental RF and psychological control
Gershy and Gray (2020)	74 parents (Israel)	MM interview coded using the MM scoring manual (Meins & Fernyhough, 2010) Single question: “describe your child”	No	Parenting behaviors (hostile and coercive)	RF significantly buffered the negative association between parenťs difficulties with emotion regulation and hostile parenting behaviors.
Gordo et al. (2020)	546 parent-child dyads (Spain)	PRFQ	No	Competent parenting Child socioemotional development	Parental competence significantly mediated the association between RF and child socioemotional development
Halfon and Besiroglu (2020)	60 parent-child dyads (Turkey)	PDI-R	No	Children’s problem behaviors	Child-focused PRF predicted less problem behavior Child mentalization negatively predicted problem behavior
León and Olhaberry (2020)	50 mother- father-child triads (Chile)	PDI-R	No	Children’s social-emotional difficulties	Mother RF predicted triadic interactions Triadic interaction mediated association between mother RF and children’s SE difficulties
Letourneau et al. (2020)	30 mother-infant dyads (56.7% White) (Canada)	PDI	Yes	PDI Strange Situation	Maternal RF improved in all three pilot studies
Mata López et al. (2020)	146 parent-child dyads & parents; 18 teachers (Chile)	Measurement of Significant Adult Mentalization in Interaction with Children	No	Children’s theory of mind Attachment Social emotional difficulties	Interaction between technician’s and caregivers’ mentalization predicted child theory of mind
Røhder et al. (2020)	78 pregnant women (Denmark)	Prenatal PRFQ	No	Maternal Antenatal Attachment	Higher PRF associated with greater quality and intensity of antenatal attachment
Slade et al. (2020)	156 pregnant women (Majority Latina) (United States)	PI PDI	Yes	PRF	Parents in the intervention group were 2.15 times more likely to be in a higher PRF group (intervention improved PRF) Interaction between condition and disrupted parent-infant communication on PRF at 24 months (intervention protected against negative effects of disrupted communication on PRF)
Suardi et al. (2020)	56 mother-child dyads (Switzerland)	WMCI	No	Maternal sensitivity Child psychopathology symptoms	Higher maternal RF related to greater maternal sensitivity Lower PRF related to child dysregulation
Væver et al. (2020)	71 mother-infant dyads (Denmark)	PEM	No	Maternal sensitivity	Higher PEM is associated with higher maternal sensitivity; not significantly different in mothers with postpartum depression and those without.
Vismara et al. (2020)	40 pregnant couples (Italy)	AAI RFS	No	Depression symptoms Parenting stress Child temperament	Mothers’ lower RF associated with higher depression symptoms, higher dysfunctional parent-child interaction, sadder tempered child Fathers’ lower RF associated with higher depressive symptoms, higher dysfunctional parent-child interaction and higher parenting stress; perceive child as less cuddly and more reactive
Waldman-Levi et al. (2020)	Mother-child dyads (Israel)	Rumination Reflection Questionnaire	No	RF	Avoidant caregiving significantly predicted lower maternal RF Anxious caregiving significantly predicted higher maternal RF Maternal RF significantly predicted maternal supportive parenting

*AAI* Adult Attachment Interview, *PDI* Parent Development Interview, *PDI-R* Parent Development Interview-Revised, *PDI-SF* Parent Development Interview- Short Form, *PI* Pregnancy Interview, *PI-R* Pregnancy Interview-Revised, *WMCI* Working Model of Child Interview, *Mini-PRFI* Parental Reflective Functioning Interview, *PEM* Parental Embodied Mentalizing, *FMSS-RF* Five-Minute Speech Sample, *PRFQ* Parental Reflective Functioning Questionnaire, *RFQ* Reflective Functioning Questionnaire.

## Data Availability

No data is available for this study.
