# Supplementary material for: Parental Reflective Capacities: A Scoping Review of Mindful Parenting and Parental Reflective Functioning
Source: Mindfulness (N Y). Author manuscript; Available in PMC 2024 Sep 26. (PMC11426413; doi:10.1007/s12671-024-02379-6)
Supplement: Supplementary Materials 1 [file NIHMS2021674-supplement-Supplementary_Materials_1.docx]

**SUPPLEMENTAL MATERIALS**

**Intervention Research and Measurement of**

**Mindful Parenting and Parental Reflective Functioning**

Here we offer a brief overview of several examples of mindful parenting and parental reflective functioning interventions and measurement tools. This overview is intended to provide illustrative examples without serving as a comprehensive review.

***Mindful Parenting Interventions***

Secular mindfulness interventions for lay audiences were initially developed in the context of medical and psychotherapeutic interventions to treat chronic pain and depression. These interventions were subsequently adapted for application to parenting. *Mindfulness-Based Stress Reduction* (MBSR; Kabat-Zinn, 1982; Kabat-Zinn, 1990) is the most well-known and well-studied mindfulness training. MBSR has been shown to reduce stress, depression, and anxiety symptoms in both clinical and non-clinical samples (Carlson & Garland, 2005; Gold et al. 2010; Goldin & Gross, 2010; Klatt et al., 2009). Mindfulness-Based Cognitive Therapy (MBCT; Segal et al., 2002; Teasdale et al., 1995) is a derivative of MBSR that is designed to reduce relapse risk in patients who have experienced depressive episodes. Studies have reported that MBCT reduces depressive symptoms and ruminative tendencies (Kenny & Williams, 2007; van Vugt et al., 2012)

In recent years, mindfulness-based interventions have been employed in parenting by teaching both formal mindfulness meditation to parents as well as skills for using mindfulness in parenting. Two studies (Bögels et al., 2008; Bögels et al., 2014) further developed Kabat-Zinn and Kabat-Zinn’s (1997) conceptualization of mindful parenting by tailoring general mindfulness exercises from MBSR and MBCT for parenting. The eight-session training facilitates mindful interaction between the parent and child by encouraging parents to cultivate nonjudgmental awareness of how they respond to their child. Instead of automatically reacting to the child, parents are taught to pay full attention to the child and cultivate an attitude of acceptance of the child’s behavior. Bögels and colleagues’ protocol has been the basis for several studies with parents (de Bruin et al., 2015; Meppelink, de Bruin et al., 2016; Potharst et al., 2019; van der Oord et al., 2012).

These studies of mindful parenting primarily modified and adjusted curricula to meet their needs in terms of hours, weeks, and modules related to specific topics (e.g., practices specifically for parents of adolescents with autism spectrum disorder). Mindful parenting training significantly benefited parents and their children, whether delivered concurrently with mindfulness programs for children or not, and whether held in-person or online. Parental mindfulness increased, and parents reported being less reactive and calmer. However, the lack of sample diversity and RCT designs in these studies makes generalization and causality difficult to establish. Thus, future research could compare mindful parenting to general mindfulness training and parent management training to better understand the effects and change mechanisms.

Some interventions target parents during specific developmental stages such as pregnancy and early parenthood. *Mindfulness-Based Childbirth and Parenting* (MBCP; Bardacke, 2012; Duncan & Bardacke, 2010) is a formal adaptation of MBSR to equip expectant parents with mindfulness skills relevant to the adaptive navigation of pregnancy, childbirth, and early parenting. MBCP offers three-hour group sessions for nine weeks to expectant parents with an emphasis on mind-body pain coping skills for childbirth as well as mindful awareness skills for coping with stress in daily life. MBCP is found to boost mindfulness and positive emotion, and improve pregnancy-related and postnatal maternal distress, anxiety, and depression symptoms into the 2^nd^ year of the child’s life (Duncan & Bardacke, 2010; Duncan et al., 2017; Warriner et al., 2018). Qualitative research with MBCP participants 2-3 years post training suggests it increases parental sensitive-responding to infant cues and toddler behaviors (Shaddix & Duncan, 2016).

Some programs have incorporated mindfulness into existing evidence-based interventions for families. The *Mindfulness-Enhanced Strengthening Families Program: For Parents and Youth 10-14* (MSFP 10-14; Duncan et al., 2009b; Coatsworth et al., 2010) is an adaptation of the Strengthening Families Program: For Parents and Youth 10-14 (SFP 10-14; Molgaard et al., 2001) that adds mindfulness and mindful parenting activities. The original SFP 10-14 program is a family-focused intervention that is designed to reduce risk factors and enhance protective factors to prevent adolescent substance use and problem behaviors that has been identified by the Cochrane collaborative as one of the best of its kind in terms of impact and longevity of effects (Foxcroft et al., 2002; Foxcroft, 2003). The newly added mindfulness activities are aimed to increase mindful parenting by enhancing parents’ ability to pay full attention and listen carefully to their children during parent-child interactions. In a large-scale RCT, the MSFP 10-14 enhanced version of the intervention (Coatsworth et al., 2015) led to greater benefits for parental monitoring, a key element of effective parent-youth relationships to prevent youth substance use. Kerr and Sttatin (2003) identified that effective parental monitoring may be less about what parents are doing to actively monitor their children, and more about children’s willingness to disclose to their parents what they are doing and who they are with. Mindful parenting, and specifically the ability to mindfully listen to children, may be an important mechanism in this disclosure process. For other aspects of the intervention, improvements from MSFP 10-14 over and above the original SFP 10-14 program were seen for fathers but not for mothers, suggesting an area for further investigation. For applications in both early childhood and early adolescence, these examples suggest a potential protective benefit of using mindfulness and mindful parenting training as a prevention approach with parents.

***Mindfulness and Mindful Parenting Measures***

In tandem with the development of mindful parenting interventions, tools to assess mindfulness and mindful parenting have been developed and tested. Initially, only self-report “dispositional” or general, trait-like mindfulness measures were available, with measures specific to mindfulness in parenting arising later. The most frequently adopted questionnaire to measure general dispositional mindfulness is the *Five Facet Mindfulness Questionnaire* (FFMQ; Baer et al., 2006). The 39-item FFMQ uses items from other mindfulness scales to assess five facets of mindfulness: 1) observing, 2) describing, 3) acting with awareness, 4) nonjudging of experience, and 5) nonreactivity to inner experience. The *Mindfulness Attention Awareness Scale* (MAAS; Brown & Ryan 2003) also measures dispositional mindfulness, and unlike the FFMQ, the MAAS captures the frequency of open and receptive attention to and awareness of ongoing events and experiences. Both FFMQ and MAAS are validated among various samples (Bohlmeijer et al., 2011; de Bruin et al., 2012; Carlson & Brown, 2005; Van Dam et al., 2010).

The *Interpersonal Mindfulness in Parenting* scale (IM-P; Duncan, 2007; 2023) is the most widely used questionnaire that specifically measures mindful parenting. The IM-P is a 31-item self-report scale that measures the five dimensions of mindful parenting proposed by Duncan and colleagues (2009a): 1) listening with full attention, 2) emotional awareness of self and child, 3) self-regulation in the parenting relationship, 4) nonjudgmental acceptance of self and child, and 5) compassion for self and child. New questionnaires of mindful parenting have been developed in recent years, including the *Bangor Mindful Parenting Scale* (BMPS; Jones et al., 2014), which measures how mindful parents are in their parenting role and interactions with their children, using similar dimensions as the FFMQ (e.g., observing, describing, acting with awareness, nonreactivity, and accepting without judgment). The *Mindfulness in Parenting Questionnaire* (MIPQ; McCaffery et al., 2017) is a more recently developed measure that assesses two dimensions of mindful parenting, mindful discipline and being in the moment with the child, which are meant to correspond to Duncan et al.’s five dimensions of mindful parenting.

***Parental Reflective Functioning Interventions***

Two main intervention programs have been developed to explicitly improve reflective functioning in parents, with a particular focus on mothers who are at high risk for low reflective functioning due to trauma or psychopathology. Minding the Baby (e.g., Sadler et al., 2013; Slade, Sadler, & Mayes, 2005) is a preventive program developed for pregnant women living in inner city environments. The program is embedded in a community health center and services are delivered by Masters-level clinicians weekly in the home beginning in the mother’s second trimester until the child’s second birthday. This program combines and builds upon the Nurse-Family Partnership (Olds et al., 2007) and infant-parent psychotherapy (Lieberman et al., 2000) intervention models, with an additional focus on enhancing maternal reflective functioning and affective communication between mother and child. Minding the Baby is one of 18 designated evidence-based home visiting programs in the United States and findings from multiple randomized control trials have demonstrated improvements in mothers’ parental reflective functioning (Slade et al., 2020).

Mothering from the Inside Out (Suchman et al., 2008, 2012, 2018) is a 12-week adjunctive individual therapy intervention for mothers in outpatient substance abuse treatment who are caring for children under five. The goal of this program is to help mothers to regulate strong affect, which can precipitate substance abuse relapse. Reflective functioning is developed through the therapeutic relationship, clarifying the mother’s core representations of herself and considering emotions derived from her representations that might drive her interactions. In the course of treatment, the therapist and mother observe video-recorded play sessions of the mother and her toddler to learn about the mother’s interpretations of the child’s behaviors and intentions and to examine mother-child synchronicity. This program was initially called the Mothers and Toddlers Program, but was later changed to Mothering from the Inside Out when the program was expanded to include older age groups (Suchman et al., 2013). This intervention has also been expanded beyond substance abusing mothers to include mothers with other mental health issues (Suchman et al., 2016). To date, multiple studies have reported significant increases in reflective functioning among mothers who participated in the Mothering Inside Out program (Suchman et al., 2010; Suchman et al., 2017).

In addition to these two main evidence-based interventions, several other programs aim to increase parental reflective functioning. Some of these programs, such as Family Minds (Bammens et al., 2015), explicitly target reflective functioning, while others include reflective functioning as one component of a larger intervention. For example, the Circle of Security- Parenting program (COS-P; Powell et al., 2014) aims to increase reflective functioning in parents to promote positive parent-child interactions and children’s attachment security (Hoffman et al., 2006; Huber et al., 2015). However, more research is needed as COS-P implementation has outpaced its rate of rigorous examination.

***Parental Reflective Functioning Measures***

A well-established approach to assessing parental reflective functioning involves using a coding protocol—the *Reflective Functioning Scale* (RFS; Fonagy et al. 1998)— applied to the *Adult Attachment Interview* (AAI; George, Kaplan, & Main, 1985). The AAI is a semi-structured interview that comprises questions that elicit parents’ autobiographical narratives about the self and their relationship with early caregivers. Over time, additional assessment tools were developed including the *Parent Development Interview* (PDI; Aber et al., 1985; PDI-R; Slade et al., 2004), which later was revised to create the *Pregnancy Interview* (PI; Slade et al., 2007), and impacted the development of the *Working Model of the Child Interview* (WMCI; Zeanah et al., 1996). Unlike the AAI, the PDI and WMCI examine a parent’s capacity to engage in reflective narratives about their child’s emotional experience or their own parenting experiences. Despite being the gold standard assessment of reflective functioning, administering and coding the interviews can be a time- and labor-intensive process. Interviews vary from 45-minutes to 1.5 hours in length and require coding by trained, certified professional. To address this barrier and expand assessment of parental reflective functioning, researchers created a self-report questionnaire, known as the *Parental Reflective Functioning Questionnaire* (PRFQ; Luyten et al., 2017). This 18-item validated scale captures three dimensions of parental reflective functioning: interest and curiosity, certainty about mental states, and pre-mentalizing, The scale was developed based on the existing coding protocols for the PDI and the Adult Attachment Interview (Luyten et al., 2017) and has demonstrated convergent validity with coded reflective functioning scores from the PDI (Anis et al., 2020). The measure has been shown to have high specificity in predicting high reflective functioning but is less sensitive to capturing low reflective functioning capacities. Therefore, the measure may be useful in community samples but is less optimal in clinical samples where participants are likely to have a lower capacity for reflective functioning (Anis et al., 2020).

**References**

Aber, J., Slade, A., Berger, B., Bresgi, I., & Kaplan, M. (1985). *The parent development interview: Interview protocol.* Unpublished manuscript: Barnard College, Columbia University, New York, NY.

Anis, L., Perez, G., Benzies, K. M., Ewashen, C., Hart, M., & Letourneau, N. (2020). Convergent validity of three measures of reflective function: Parent development interview, parental reflective function questionnaire, and reflective function questionnaire. *Frontiers in Psychology, 11,* 1–14. https://doi.org/10.3389/fpsyg.2020.574719

Baer, R. A., Smith, G. T., Hopkins, J., Krietemeyer, J., & Toney, L. (2006). Using self-report assessment methods to explore facets of mindfulness. *Assessment*, *13*(1)*,* 27–45. https://doi.org/10.1177/1073191105283504.

Bammens, A. S., Adkins, T., & Badger, J. (2015). Psycho-educational intervention increases reflective functioning in foster and adoptive parents. *Adoption and Fostering*, *39*(1), 38–50. https://doi.org/10.1177/0308575914565069.

Bardacke, N. (2012). *Mindful birthing: training the mind, body, and heart for childbirth and beyond*. Harper Collins.

Bögels, S. M., & Restifo, K. (2014). *Mindful Parenting: A Guide for Mental Health Practitioners.* Springer.

Bögels, S. M., Hoogstad, B., Van Dun, L., De Schutter, S., & Restifo, K. (2008). Mindfulness training for adolescents with externalizing disorders and their parents. *Behavioural and Cognitive Psychotherapy*, *36*(2), 193–209. https://doi.org/10.1017/S1352465808004190.

Bohlmeijer, E., Klooster, P. M., Fledderus, M., Veehof, M., & Baer, R. (2011). Psychometric properties of the Five Facet Mindfulness Questionnaire in depressed adults and development of a short form. *Assessment*, *18*(3), 308–320. https://doi.org/10.1177/1073191111408231.

Brown, K. W., & Ryan, R. M. (2003). The benefits of being present: Mindfulness and its role in psychological well-being. *Journal of Personality and Social Psychology*, *84*(4), 822-848. https://doi.org/10.1037/0022-3514.84.4.822.

Carlson, L. E., & Brown, K. W. (2005). Validation of the Mindful Attention Awareness Scale in a cancer population. *Journal of Psychosomatic Research*, *58*(1), 29–33. https://doi.org/10.1016/j.jpsychores.2004.04.366.

Carlson, L. E., & Garland, S. N. (2005). Impact of mindfulness-based stress reduction (MBSR) on sleep, mood, stress and fatigue symptoms in cancer outpatients. *International Journal of Behavioral Medicine*, *12*(4), 278–285. https://doi.org/10.1207/s15327558ijbm1204_9.

Coatsworth, J. D., Duncan, L. G., Greenberg, M. T., & Nix, R. L. (2010). Changing parent’s mindfulness, child management skills and relationship quality with their youth: Results from a randomized pilot intervention trial. *Journal of Child and Family Studies*, *19*, 203–217. https://doi.org/10.1007/s10826-009-9304-8.

Coatsworth, J. D., Duncan, L. G., Nix, R. L., Greenberg, M. T., Bamberger, K., Gayles, J. G., Berrena, E., & Demi, M. A. (2015). Integrating mindfulness with parent training: Effects of the Mindfulness-enhanced Strengthening Families Program. *Developmental Psychology, 51(1)*, 26-35. https://doi.org/10.1037/a0038212

de Bruin, E. I., Blom, R., Smit, F. M. A., Van Steensel, F. J. A., & Bögels, S. M. (2015). MYmind: Mindfulness training for youngsters with autism spectrum disorders and their parents. *Autism*, *19*(8), 906–914. https://doi.org/10.1177/1362361314553279.

de Bruin, E. I., Topper, M., Muskens, J. G. A. M., Bögels, S. M., & Kamphuis, J. H. (2012). Psychometric properties of the Five Facets Mindfulness Questionnaire (FFMQ) in a meditating and a non-meditating sample. *Assessment*, *19*(2), 187–197. https://doi.org/10.1177/1073191112446654.

Duncan, L. G. (2007). *Assessment of mindful parenting among parents of early adolescents: Development and validation of the Interpersonal Mindfulness in Parenting scale* (Doctoral dissertation, The Pennsylvania State University). Retrieved from https://etda.libraries.psu.edu/files/final_submissions/3737.

Duncan, L. G. (2023). Interpersonal Mindfulness in Parenting (IM-P) Scale. In O. N. Medvedev, C. U. Krägeloh, R. J. Siebert, & N. N. Singh (Eds.). *Handbook of Assessment in Mindfulness Research* (Living edition). Spring Nature reference. https://doi.org/10.1007/978-3-030-77644-2

Duncan, L. G., & Bardacke, N. (2010). Mindfulness-based childbirth and parenting education: Promoting family mindfulness during the perinatal period. *Journal of Child and Family Studies*, *19*, 190–202. https://doi.org/10.1007/s10826-009-9313-7.

Duncan, L. G., Coatsworth, J. D., & Greenberg, M. T. (2009a). A model of mindful parenting: Implications for parent-child relationships and prevention research. *Clinical Child and Family Psychology Review*, *12*, 255–270. https://doi.org/10.1007/s10567-009-0046-3

Duncan, L. G., Coatsworth, J. D., & Greenberg, M. T. (2009b). Pilot study to gauge acceptability of a mindfulness-based, family-focused preventive intervention. *Journal of Primary Prevention, 30*, 605-618. https://doi.org/10.1007/s10935-009-0185-9

Duncan, L. G., Cohn, M. A., Chao, M. T., Cook, J. G., Riccobono, J., & Bardacke, N. (2017). Benefits of preparing for childbirth with mindfulness training: A randomized controlled trial with active comparison. *BMC Pregnancy and Childbirth*, *17*(1), 2-11. https://doi.org/10.1186/s12884-017-1319-3.

Fonagy, P., Target, M., Steele, H., & Steele, M. (1998). *Reflective-functioning manual, version 5.0, for application to adult attachment interviews.* Unpublished manuscript, University College London, England.

Foxcroft, D. R., Ireland, D., Lister‐Sharp, D. J., Lowe, G., & Breen, R. (2003). Longer‐term primary prevention for alcohol misuse in young people: A systematic review. *Addiction*, *98*(4), 397-411. https://doi.org/10.1046/j.1360-0443.2003.00355.x

Foxcroft, D.R. et al. “Primary Prevention for Alcohol Misuse in Young People (Cochrane Review)." In: *The Cochrane Library: 2002*, 4. Oxford.

George, C., Kaplan, N., & Main, M. (1985). *Attachment interview for adults.* Unpublished manuscript, University of California, Berkeley.

Gold, E., Smith, A., Hopper, I., Herne, D., Tansey, G., & Hulland, C. (2010). Mindfulness-Based Stress Reduction (MBSR) for primary school teachers. *Journal of Child and Family Studies*, *19*, 184–189. https://doi.org/10.1007/s10826-009-9344-0.

Goldin, P. R., & Gross, J. J. (2010). Effects of Mindfulness-Based Stress Reduction (MBSR) on emotion regulation in social anxiety disorder. *Emotion*, *10*(1), 83–91. https://doi.org/10.1037/a0018441.

Hoffman, K. T., Marvin, R. S., Cooper, G., & Powell, B. (2006). Changing toddlers’ and preschoolers’ attachment classifications: The Circle of Security intervention. *Journal of Consulting and Clinical Psychology*, *7*(6), 1017–1026. https://doi.org/10.1037/0022-006X.74.6.1017

Huber, A., McMahon, C. A., & Sweller, N. (2015). Efficacy of the 20‐week circle of security intervention: Changes in caregiver reflective functioning, representations, and child attachment in an Australian clinical sample. *Infant Mental Health Journal*, *36*(6), 556-574. https://doi.org/10.1002/imhj.21540

Jones, L., Hastings, R. P., Totsika, V., Keane, L., & Rhule, N. (2014). Child behavior problems and parental well-being in families of children with autism: The mediating role of mindfulness and acceptance. *American Journal on Intellectual and Developmental Disabilities*, *119*(2), 171–185. https://doi.org/10.1352/1944-7558-119.2.171.

Kabat-Zinn, J. (1982). An outpatient program in behavioral medicine for chronic pain patients based on the practice of mindfulness meditation: Theoretical considerations and preliminary results. *General Hospital Psychiatry: Psychiatry, Medicine and Primary Care, 4*(1), 33-47.

Kabat-Zinn, J. (1990). *Full catastrophe living: Using the wisdom of your body and mind to face stress, pain, and illness*. Delacorte Press.

Kabat-Zinn, M., & Kabat-Zinn, J. (1997). *Everyday blessings: The inner work of mindful parenting.* Hyperion.

Kenny, M. A., & Williams, J. M. G. (2007). Treatment-resistant depressed patients show a good response to mindfulness-based cognitive therapy. *Behaviour Research and Therapy*, *45*(3), 617–625. https://doi.org/10.1016/j.brat.2006.04.008.

Kerr, M., & Stattin, H. (2003). Parenting of adolescents: Action or reaction? In A. C. Crouter & A. Booth (Eds.), *Children's Influence on Family Dynamics: The Neglected Side of Family Relationships* (pp. 121-151). Lawrence Erlbaum Associates.

Klatt, M. D., Buckworth, J., & Malarkey, W. B. (2009). Effects of Low-Dose Mindfulness-Based Stress Reduction (MBSR-ld) on working adults. *Health Education and Behavior*, *36*, 601–614. https://doi.org/10.1177/1090198108317627

Lieberman, A. F., Silverman, R., & Pawl, J. H. (2000). Infant–parent psychotherapy: Core concepts and current approaches. In C. H. Zeanah Jr. (Ed.), *Handbook of infant mental health.*, 2nd ed. (pp. 472–484). The Guilford Press.

Luyten, P., Mayes, L. C., Nijssens, L., & Fonagy, P. (2017). The parental reflective functioning questionnaire: Development and preliminary validation. *PLoS ONE*, *12*(5), 1-28. https://doi.org/10.1371/journal.pone.0176218.

McCaffrey, S., Reitman, D., & Black, R. (2017). Mindfulness in Parenting Questionnaire (MIPQ): Development and validation of a measure of mindful parenting. *Mindfulness*, *8*, 232-246. https://doi.org/10.1007/s12671-016-0596-7

Meppelink, R., de Bruin, E. I., Wanders-Mulder, F. H., Vennik, C. J., & Bögels, S. M. (2016). Mindful parenting training in child psychiatric settings: Heightened parental mindfulness reduces parents’ and children’s psychopathology. *Mindfulness*, *7*, 680–689. https://doi.org/10.1007/s12671-016-0504-1.

Molgaard, V., Kumpfer, K. L., & Fleming, E. (2001). *The Strengthening Families Program: For Parents and Youth 10-14; A video-based curriculum*. Iowa State University Extension.

Olds, D. L., Sadler, L., & Kitzman, H. (2007). Programs for parents of infants and toddlers: Recent evidence from randomized trials. *Journal of Child Psychology and Psychiatry, 48*(3-4), 355–391. https://doi.org./10.1111/j.1469-7610.2006.01702.x.

Potharst, E. S., Boekhorst, M. G. B. M., Cuijlits, I., van Broekhoven, K. E. M., Jacobs, A., Spek, V., Nyklíček, I., Bögels, S. M., & Pop, V. J. M. (2019). A randomized control trial evaluating an online mindful parenting training for mothers with elevated parental stress. *Frontiers in Psychology*, *10,* 1-15. https://doi.org/10.3389/fpsyg.2019.01550

Powell, B., Cooper, G., Hoffman, K., & Marvin, B. (2014). *The Circle of Security intervention: Enhancing attachment in early parent-child relationships*. Guilford Press.

Sadler, L. S., Slade, A., Close, N., Webb, D. L., Simpson, T., Fennie, K., & Mayes, L. C. (2013). Minding the Baby: Enhancing reflectiveness to improve early health and relationship outcomes in an interdisciplinary home-visiting program. *Infant Mental Health Journal*, *34*(5), 391–405. https://doi.org/10.1002/imhj.21406.

Segal, Z. V., Williams, J. M. G., & Teasdale, J. D. (2002). *Mindfulness-based cognitive therapy for depression: A new approach to preventing relapse.* Guilford Press.

Shaddix, C. & Duncan, L. G. (2016). Mindful parenting begins at the beginning: Potential benefits of perinatal meditation training for family well-being. In T. Lomas (Ed.), *Mindfulness in Positive Psychology: The Science of Meditation and Wellbeing*. Routledge.

Slade, A., Aber, J. L., Bresgi, I., Berger, B., & Kaplan, M. (2004). *The Parent Development Interview – Revised.* Unpublished protocol. City University of New York.

Slade, A., Grunebaum, L., Huganir, L., & Reeves, M. (2007). *The pregnancy interview-revised.* City College of New York.

# Slade, A., Holland, M. L., Ordway, M. R., Carlson, E. A., Jeon, S., Close, N., Mayes, L.C., & Sadler, L. S. (2020). Minding the Baby®: Enhancing parental reflective functioning and infant attachment in an attachment-based, interdisciplinary home visiting program. *Development and Psychopathology, 32,* 123-137. https://doi.org/10.1017/S0954579418001463

Slade, A., Sadler, L. S., & Mayes, L. C. (2005). Minding the Baby: Enhancing parental reflective functioning in a nursing/mental health home visiting program. In L. J. Berlin, Y. Ziv, L. Amaya-Jackson, & M. T. Greenberg (Eds.), *Enhancing early attachments: Theory, research, intervention, and policy*. (pp. 152–177). Guilford Press.

# Suchman, N. E., DeCoste, C. L., McMahon, T. J., Dalton, R., Mayes, L. C., & Borelli, J. (2017). Mothering from the Inside Out: Results of a second randomized clinical trial testing a mentalization-based intervention for mothers in addiction treatment. Development and Psychopathology, 29, 617–636. https://doi.org/10.1017/S0954579417000220

# Suchman, N. E., DeCoste, C., Borelli, J. L., & McMahon, T. J. (2018). Does improvement in maternal attachment representations predict greater maternal sensitivity, child attachment security and lower rates of relapse to substance use? A second test of Mothering from the Inside Out treatment mechanisms. *Journal of Substance Abuse Treatment, 85,* 21–30. https://doi.org/10.1016/j.jsat.2017.11.006

# Suchman, N. E., DeCoste, C., Castiglioni, N., McMahon, T. J., Rounsaville, B., & Mayes, L. (2010). The mothers and toddlers program, an attachment-based parenting intervention for substance using women: Post-treatment results from a randomized clinical pilot. *Attachment and Human Development, 12,* 483–504. https://doi.org/10.1080/14616734.2010.501983

Suchman, N. E., DeCoste, C., Rosenberger, P., & Mcmahon, T. J. (2012). Attachment-based intervention for substance-using mothers: A preliminary test of the proposed mechanisms of change. *Infant Mental Health Journal*, *33*(4), 360–371. https://doi.org/10.1002/imhj.21311.

# Suchman, N., DeCoste, C., Castiglioni, N., Legow, N., & Mayes, L. (2008). The Mothers and Toddlers program: Preliminary findings from an attachment-based parenting intervention for substance-abusing mothers. *Psychoanalytic Psychology, 25,* 499–517. https://doi.org/10.1037/0736-9735.25.3.499

Teasdale, J. D., Segal, Z., & Williams, J. M. G. (1995). How does cognitive therapy prevent depressive relapse and why should attentional control (mindfulness) training help? *Behaviour Research and Therapy*, *33*(1), 25–39. https://doi.org/10.1016/0005-7967(94)E0011-7.

Van Dam, N. T., Earleywine, M., & Borders, A. (2010). Measuring mindfulness? An item response theory analysis of the mindful attention awareness scale. *Personality and Individual Differences*, *49*(7), 805–810. https://doi.org/10.1016/j.paid.2010.07.020

van der Oord, S., Bögels, S. M., & Peijnenburg, D. (2012). The effectiveness of mindfulness training for children with ADHD and mindful parenting for their parents. *Journal of Child and Family Studies*, *21*, 139–147. https://doi.org/10.1007/s10826-011-9457-0.

van Vugt, M. K., Hitchcock, P., Shahar, B., & Britton, W. (2012). The effects of mindfulness-based cognitive therapy on affective memory recall dynamics in depression: A mechanistic model of rumination. *Frontiers in Human Neuroscience*, *19*, 257. https://doi.org/10.3389/fnhum.2012.00257.

Warriner, S., Crane, C., Dymond, M., & Krusche, A. (2018). An evaluation of mindfulness-based childbirth and parenting courses for pregnant women and prospective fathers/partners within the UK NHS (MBCP-4-NHS). *Midwifery*, *64*, 1–10. https://doi.org/10.1016/j.midw.2018.05.004.

Zeanah, C.H., Benoit, D., Barton, M.L., & Hirshberg, L. (1996). Working model of the child interview coding manual. Unpublished manuscript.
