# Supplementary material for: Parental Reflective Capacities: A Scoping Review of Mindful Parenting and Parental Reflective Functioning
Source: Mindfulness (N Y). Author manuscript; Available in PMC 2024 Sep 26. (PMC11426413; doi:10.1007/s12671-024-02379-6)
Supplement: Supplementary Materials 2 [file NIHMS2021674-supplement-Supplementary_Materials_2.docx]

**MP and PRF References (N = 301 articles)**

# Aalders, J., Hartman, E., Nefs, G., Nieuwesteeg, A., Hendrieckx, C., Aanstoot, H. J., Winterdijk, A.P., van Mil, E., and Pouwer, F. (2018). Mindfulness and fear of hypoglycaemia in parents of children with Type 1 Diabetes: Results from diabetes MILES youth – The Netherlands. *Diabetic Medicine, 35*, 650–657. https://doi.org/10.1111/dme.13594.

# Adkins, T., Luyten, P., & Fonagy, P. (2018). Development and preliminary evaluation of Family Minds: A mentalization-based psychoeducation program for foster parents. *Journal of Child and Family Studies, 27*, 2519–2532. https://doi.org/10.1007/s10826-018-1080-x

# Álvarez, C., Cristi, P., Del Real, M. T., & Farkas, C. (2019). Mentalization in Chilean mothers with children aged 12 and 30 months: Relation to child sex and temperament and family socioeconomic status. *Journal of Child and Family Studies, 28*, 959-970.

# Alvarez-Monjarás, M., McMahon, T. J., & Suchman, N. E. (2019). Does maternal reflective functioning mediate associations between representations of caregiving with maternal sensitivity in a high-risk sample? *Psychoanalytic Psychology, 36,* 82–92. https://doi.org/10.1037/pap0000166

# Anderson, K., & van Ee, E. (2020). Reflective functioning of refugee mothers with children born of conflict-related sexual violence. *International Journal of Environmental Research and Public Health, 17,* 1-12.

# Arikan, G., & Kumru, A. (2020). Patterns of associations between maternal symptoms and child problem behaviors: the mediating role of mentalization, negative intentionality, and unsupportive emotion socialization. *Child Psychiatry & Human Development*, 1-14.

# Ashton, C. K., O’Brien-Langer, A., & Silverstone, P. H. (2016). The CASA Trauma and Attachment Group (TAG) program for children who have attachment issues following early developmental trauma. *Journal of the Canadian Academy of Child and Adolescent Psychiatry, 25,* 35–42.

# Bakhshayesh, S. H., Khishvand, S., & Siavosh, H. (2015). The effectiveness of mindfulness training for children with ADHD and mindful parenting for their parents. *International Journal of Review in Life Science, 5,* 1506–1511. https://doi.org/10.1007/s10826-011-9457-0

# Bammens, A. S., Adkins, T., & Badger, J. (2015). Psycho-educational intervention increases reflective functioning in foster and adoptive parents. *Adoption and Fostering, 39,* 38–50. https://doi.org/10.1177/0308575914565069

# Barone, L., & Carone, N. (2020). Childhood abuse and neglect experiences, Hostile-Helpless attachment, and reflective functioning in mentally ill filicidal mothers. *Attachment & Human Development,* 1-24.

# Barrio Martínez, L., Martínez-pampliega, A., & Merino Ramos, L. (2020). Mindful parenting: A pilot study of the “Brief Mindfulness Intervention Program” (BMIP) in the educational context. Journal of Evidence-Based Psychotherapies, 20(1), 77–92.

# Bazzano, A., Wolfe, C., Zylowska, L., Wang, S., Schuster, E., Barrett, C., & Lehrer, D. (2015). Mindfulness based stress reduction (MBSR) for parents and caregivers of individuals with developmental disabilities: A community-based approach. *Journal of Child and Family Studies, 24*, 298–308. https://doi.org/10.1007/s10826-013-9836-9

# Beer, M., Ward, L., & Moar, K. (2013). The relationship between mindful parenting and distress in parents of children with an autism spectrum disorder. *Mindfulness, 4*, 102–112. https://doi.org/10.1007/s12671-012-0192-4

# Behbahani, M., Zargar, F., Assarian, F., & Akbari, H. (2018). Effects of mindful parenting training on clinical symptoms in children with attention deficit hyperactivity disorder and parenting stress: Randomized controlled trial. *Iranian Journal of Medical Sciences, 43,* 596–604.

# Benbassat, N., & Priel, B. (2012). Parenting and adolescent adjustment: The role of parental reflective function. *Journal of Adolescence, 35,* 163–174. https://doi.org/10.1016/j.adolescence.2011.03.004

# Benn, R., Akiva, T., Arel, S., & Roeser, R. W. (2012). Mindfulness training effects for parents and educators of children with special needs. *Developmental Psychology, 48*, 1476–1487. https://doi.org/10.1037/a0027537.

# Benton, J., Coatsworth, D., & Biringen, Z. (2019). Examining the association between emotional availability and mindful parenting. *Journal of Child and Family Studies, 28* (6), 1650-1663*.* https://doi.org/10.1007/s10826-019-01384-x

# Berthelot, N., Ensink, K., Bernazzani, O., Normandin, L., Luyten, P., & Fonagy, P. (2015). Intergenerational transmission of attachment in abused and neglected mothers: The role of trauma-specific reflective functioning. *Infant Mental Health Journal, 36*, 200–212. https://doi.org/10.1002/imhj.21499

# Berthelot, N., Lemieux, R., Garon-Bissonnette, J., Lacharité, C., & Muzik, M. (2019). The protective role of mentalizing: Reflective functioning as a mediator between child maltreatment, psychopathology and parental attitude in expecting parents. *Child Abuse and Neglect, 95.* Advance online publication. https://doi.org/10.1016/j.chiabu.2019.104065

# Bluth, K., & Wahler, R. G. (2011a). Does effort matter in mindful parenting? *Mindfulness, 2*, 175–178. https://doi.org/10.1007/s12671-011-0056-3

# Bluth, K., & Wahler, R. G. (2011b). Parenting preschoolers: Can mindfulness help? *Mindfulness, 2,* 282–285. https://doi.org/10.1007/s12671-011-0071-4

# Boekhorst, M. G. B. M., Hulsbosch, L. P., Nyklíček, I., Spek, V., Kastelein, A., Bögels, S., Pop, V. J. M., & Potharst, E. S. (2020). An Online Mindful Parenting Training for mothers raising toddlers: Assessment of acceptability, effectiveness, and personal goals. *Mindfulness, 12*(2), 519–531. https://doi.org/10.1007/s12671-020-01542-z

# Bögels, S. M., Hellemans, J., van Deursen, S., Römer, M., & van der Meulen, R. (2014). Mindful parenting in mental health care: Effects on parental and child psychopathology, parental stress, parenting, coparenting, and marital functioning. *Mindfulness, 5,* 536–551. https://doi.org/10.1007/s12671-013-0209-7

# Bögels, S., Hoogstad, B., Van Dun, L., De Schutter, S., & Restifo, K. (2008). Mindfulness training for adolescents with externalizing disorders and their parents. Behavioural and *Cognitive Psychotherapy, 36*, 193–209. https://doi.org/10.1017/S1352465808004190

# Borelli, J. L., Cohen, C., Pettit, C., Normandin, L., Target, M., Fonagy, P., & Ensink, K. (2019). Maternal and child sexual abuse history: An intergenerational exploration of children’s adjustment and maternal trauma-reflective functioning. *Frontiers in Psychology, 10*, 1-11. https://doi.org/10.3389/fpsyg.2019.01062

# Borelli, J. L., Ensink, K., Gillespie, M. L., Falasiri, E., Bernazzani, O., Fonagy, P., & Berthelot, N. (2020). Mothers’ self‐focused reflective functioning interacts with childhood experiences of rejection to predict current romantic relationship quality and parenting behavior. *Family Process*, 1-15.

# Borelli, J. L., Lai, J., Smiley, P. A., Kerr, M. L., Buttitta, K., Hecht, H. K., & Rasmussen, H. F. (2020). Higher maternal reflective functioning is associated with toddlers’ adaptive emotion regulation. *Infant Mental Health Journal,* 1-15.

# Borelli, J. L., St. John, H. K., Cho, E., & Suchman, N. E. (2016). Reflective functioning in parents of school-aged children. *American Journal of Orthopsychiatry, 86*, 25–36. https://doi.org/10.1037/ort0000141

# Borelli, J. L., Stern, J. A., Marvin, M. J., Smiley, P. A., Pettit, C., & Samudio, M. (2020, March 19). Reflective Functioning and Empathy Among Mothers of School-Aged Children: Charting the Space Between. *Emotion*, 1-18.

# Borelli, J. L., West, J. L., Decoste, C., & Suchman, N. E. (2012). Emotionally avoidant language in the parenting interviews of substance-dependent mothers: Associations with reflective functioning, recent substance use, and parenting behavior. *Infant Mental Health Journal, 33,* 506–519. https://doi.org/10.1002/imhj.21340

# Brown, S. M., Bender, K. A., Bellamy, J. L., Garland, E. L., Dmitrieva, J., & Jenson, J. M. (2018). A pilot randomized trial of a mindfulness-informed intervention for child welfare-involved families. Mindfulness. Advance online publication. https://doi.org/10.1007/s12671-018-1025-x

# Bunday, L., Dallos, R., Morgan, K., & McKenzie, R. (2015). Foster carers’ reflective understandings of parenting looked after children: An exploratory study. Adoption and Fostering, 39, 145–158. https://doi.org/10.1177/0308575915588730

# Burke, L. K., McGill, J., & Adler-Baeder, F. (2020). Exploring the links between facets of mindfulness and parenting efficacy and stress. *Journal of Child and Family Studies, 29*(6), 1679–1691. https://doi.org/10.1007/s10826-019-01692-2

# Burkhart, M. L., Borelli, J. L., Rasmussen, H. F., Brody, R., & Sbarra, D. A. (2017). Parental mentalizing as an indirect link between attachment anxiety and parenting satisfaction. *Journal of Family Psychology, 31,* 203–213. https://doi.org/10.1037/fam0000270

# Buttitta, K. V., Smiley, P. A., Kerr, M. L., Rasmussen, H. F., Querdasi, F. R., & Borelli, J. L. (2019). In a father’s mind: Paternal reflective functioning, sensitive parenting, and protection against socioeconomic risk. *Attachment and Human Development, 21*(5), 445-466. https://doi.org/10.1080/14616734.2019.1582596

# Byrne, G., Sleed, M., Midgley, N., Fearon, P., Mein, C., Bateman, A., & Fonagy, P. (2018). Lighthouse Parenting Programme: Description and pilot evaluation of a mentalization-based treatment to address child maltreatment. *Clinical Child Psychology and Psychiatry*, *24*(4), 680-693. https://doi.org/10.1177/1359104518807741

# Calvete, E., Gómez-Odriozola, J., & Orue, I. (2020). Differential susceptibility to the benefits of mindful parenting depending on child dispositional mindfulness. *Mindfulness, 12*(2), 405–418. https://doi.org/10.1007/s12671-020-01467-7

# Campbell, K., Thoburn, J. W., & Leonard, H. D. (2017). The mediating effects of stress on the relationship between mindfulness and parental responsiveness. *Couple and Family Psychology: Research and Practice, 6,* 48–59. https://doi.org/10.1037/CFP0000075

# Campora, G., Giromini, L., Guerriero, V., Chiodo, C., Zavattini, G. C., & Larciprete, G. (2019). Influence of maternal reflective functioning on mothers’ and children’s weight: A follow‐up study. *Infant Mental Health Journal*, *40*(6), 862-873. https://doi.org/10.1002/imhj.21819

# Carlone, C., & Milan, S. (2020). Does your child need therapy? Maternal reflective functioning and perceived need for and use of child mental health treatment. *Attachment & Human Development*, *23*(3), 310-327.

# Chan, N., & Neece, C. L. (2018). Parenting stress and emotion dysregulation among children with developmental delays: The role of parenting behaviors. *Journal of Child and Family Studies, 27*, 4071–4082. https://doi.org/10.1007/s10826-018-1219-9

# Chan, T. O., & Lam, S. F. (2017). Mediator or moderator? The role of mindfulness in the association between child behavior problems and parental stress. *Research in Developmental Disabilities, 70*, 1–10. https://doi.org/10.1016/j.ridd.2017.08.007

# Chaplin, T. M., Turpyn, C. C., Fischer, S., Martelli, A. M., Ross, C. E., Leichtweis, R. N., … Sinha, R. (2018). Parenting-focused mindfulness intervention reduces stress and improves parenting in highly stressed mothers of adolescents. Mindfulness, *12*(2), 450-462. https://doi.org/10.1007/s12671-018-1026-9

# Cheung, R. Y. M., Leung, S. S. W., & Mak, W. W. S. (2019). Role of mindful parenting, affiliate stigma, and parents’ well-being in the behavioral adjustment of children with autism spectrum disorder: Testing parenting stress as a mediator. Mindfulness, *10*(11), 2352-2362. https://doi.org/10.1007/s12671-019-01208-5

# Claydon, E., Zerwas, S., Callinan, L., & Smith, M. V. (2016). Parental reflective functioning among mothers with eating disorder symptomatology. *Eating Behaviors, 23,* 141–144. http://dx.doi.org/10.1016/j.eatbeh.2016.09.002

# Coatsworth, J. D., Duncan, L. G., Greenberg, M. T., & Nix, R. L. (2010). Changing parent’s mindfulness, child management skills and relationship quality with their youth: Results from a randomized pilot intervention trial*. Journal of Child and Family Studies, 19*, 203–217. https://doi.org/10.1007/s10826-009-9304-8

# Coatsworth, J. D., Duncan, L. G., Nix, R. L., Greenberg, M. T., Gayles, J. G., Bamberger, K. T., … Demi, M. A. (2015). Integrating mindfulness with parent training: Effects of the mindfulness-enhanced strengthening families program. *Developmental Psychology, 51*, 26–35. https://doi.org/10.1037/a0038212

# Coatsworth, J. D., Timpe, Z., Nix, R. L., Duncan, L. G., & Greenberg, M. T. (2018). Changes in mindful parenting: Associations with changes in parenting, parent–youth relationship quality, and youth behavior. *Journal of the Society for Social Work and Research, 9,* 511–529. https://doi.org/10.1086/701148

# Conner, C. M., & White, S. W. (2014). Stress in mothers of children with autism: Trait mindfulness as a protective factor. *Research in Autism Spectrum Disorders, 8,* 617–624. https://doi.org/10.1016/j.rasd.2014.02.001

# Cooke, D., Priddis, L., Luyten, P., Kendall, G., & Cavanagh, R. (2017). Paternal and maternal reflective functioning in the western Australian peel child health study. *Infant Mental Health Journal, 38*, 561–574. https://doi.org/10.1002/imhj.21664

# Cordes, K., Smith-Nielsen, J., Tharner, A., Katznelson, H., Steele, H., & Væver, M. (2017). Reflective functioning in postpartum depressed women with and without comorbid personality disorder. *Psychoanalytic Psychology, 34,* 414–421. https://doi.org/10.1037/pap0000135

# Corthorn, C. (2018). Benefits of mindfulness for parenting in mothers of preschoolers in Chile. *Frontiers in Psychology, 9*, 1-11. https://doi.org/10.3389/fpsyg.2018.01443

# Corthorn, C., & Milicic, N. (2016). Mindfulness and parenting: A correlational study of non-meditating mothers of preschool children. *Journal of Child and Family Studies, 25*, 1672–1683. https://doi.org/10.1007/s10826-015-0319-z

# Cristobal, P. S., Santelices, M. P., & Fuenzalida, D. A. M. (2017). Manifestation of trauma: The effect of early traumatic experiences and adult attachment on parental reflective functioning. *Frontiers in Psychology, 8*, 1-9. https://doi.org/10.3389/fpsyg.2017.00449

# Dawe, S., & Harnett, P. (2007). Reducing potential for child abuse among methadone-maintained parents: Results from a randomized controlled trial. *Journal of Substance Abuse Treatment, 32*, 381–390. https://doi.org/10.1016/j.jsat.2006.10.003

# De Bruin, E. I., Blom, R., Smit, F. M. A., Van Steensel, F. J. A., & Bögels, S. M. (2015). MYmind: Mindfulness training for youngsters with autism spectrum disorders and their parents. *Autism, 19,* 906–914. https://doi.org/10.1177/1362361314553279

# Dehkordian, P., Hamid, N., Beshlideh, K., & Honarmand, M. M. (2017). The effectiveness of mindful parenting, social thinking and exercise on quality of life in ADHD children. *International Journal of Pediatrics, 5,* 4295–4302. https://doi.org/10.22038/ijp.2016.7900

# Dejko–Wańczyk, K., Janusz, B., & Józefik, B. (2020). Understanding the Externalizing Behavior of School-Age Boys: The Role of a Mother’s Mentalization and Attachment. *Journal of Child and Family Studies, 29,* 155-166.

# Dieleman, L. M., Soenens, B., De Pauw, S. S., Prinzie, P., Vansteenkiste, M., & Luyten, P. (2020). The Role of Parental Reflective Functioning in the Relation between Parents’ Self-Critical Perfectionism and Psychologically Controlling Parenting Towards Adolescents. *Parenting, 20,* 1-27.

# Dieleman, L. M., Soenens, B., Prinzie, P., de Clercq, L., Ortibus, E., & de Pauw, S. S. W. (2020). Daily parenting of children with cerebral palsy: The role of daily child behavior, parents’ daily psychological needs, and mindful parenting. *Development and Psychopathology, 33*(1), 184–200. https://doi.org/10.1017/S0954579419001688

# Duncan, L. G., & Bardacke, N. (2010). Mindfulness-based childbirth and parenting education: Promoting family mindfulness during the perinatal period. Journal of Child and Family Studies, 19, 190–202. https://doi.org/10.1007/s10826-009-9313-7

# Duncan, L. G., Coatsworth, J. D., & Greenberg, M. T. (2009). Pilot study to gauge acceptability of a mindfulness-based, family-focused preventive intervention. *Journal of Primary Prevention, 30, 605*–618. https://doi.org/10.1007/s10935-009-0185-9

# Duncan, L. G., Coatsworth, J. D., Gayles, J. G., Geier, M. H., & Greenberg, M. T. (2015). Can mindful parenting be observed? Relations between observational ratings of mother-youth interactions and mothers’ self-report of mindful parenting. *Journal of Family Psychology, 29,* 276–282. https://doi.org/10.1037/a0038857

# Duncan, L. G., Cohn, M. A., Chao, M. T., Cook, J. G., Riccobono, J., & Bardacke, N. (2017). Benefits of preparing for childbirth with mindfulness training: A randomized controlled trial with active comparison. *BMC Pregnancy and Childbirth, 17*, 1-11. https://doi.org/10.1186/s12884-017-1319-3

# Eames, C., Crane, R., Gold, E., & Pratt, S. (2015). Mindfulness-based wellbeing for socio-economically disadvantaged parents: A pre-post pilot study. *Journal of Children’s Services, 10*, 17–28. https://doi.org/10.1108/JCS-09-2014-0040

# Elgendy, R. S., el Malky, M. I., & Ebrahem, S. M. (2020). Mindful parenting and stress among parents with children having Attention Deficit Hyperactivity Disorder. International *Journal of Novel Research in Healthcare and Nursing, 7*(2), 293–305. www.noveltyjournals.com

# Eltelt, R. M. H., & Mostafa, M. M. (2019). Mindfulness-Based Intervention program on stress reduction during pregnancy. *American Journal of Nursing Research, 7*(3), 375–386. https://doi.org/10.12691/ajnr-7-3-19

# Emerson, L. M., Aktar, E., de Bruin, E., Potharst, E., & Bögels, S. (2019). Mindful parenting in secondary child mental health: Key parenting predictors of treatment effects. *Mindfulness*, *12*(2), 532-542. https://doi.org/10.1007/s12671-019-01176-w

# Enav, Y., Erhard-Weiss, D., Kopelman, M., Samson, A. C., Mehta, S., Gross, J. J., & Hardan, A. Y. (2019). A non-randomized mentalization intervention for parents of children with autism. *Autism Research, 12,* 1077–1086. https://doi.org/10.1002/aur.2108

# Ensink, K., Bégin, M., Normandin, L., & Fonagy, P. (2016). Maternal and child reflective functioning in the context of child sexual abuse: Pathways to depression and externalising difficulties. *European Journal of Psychotraumatology, 7*, 1-10. https://doi.org/10.3402/ejpt.v7.30611

# Ensink, K., Bégin, M., Normandin, L., & Fonagy, P. (2017). Parental reflective functioning as a moderator of child internalizing difficulties in the context of child sexual abuse. Psychiatry Research, 257, 361–366. https://doi.org/10.1016/j.psychres.2017.07.051

# Ensink, K., Borelli, J. L., Roy, J., Normandin, L., Slade, A., & Fonagy, P. (2019). Costs of not getting to know you: Lower levels of parental reflective functioning confer risk for maternal insensitivity and insecure infant attachment. *Infancy, 24,* 210–227. https://doi.org/10.1111/infa.12263

# Ensink, K., Normandin, L., Plamondon, A., Berthelot, N., & Fonagy, P. (2016). Intergenerational pathways from reflective functioning to infant attachment through parenting. Canadian *Journal of Behavioural Science. 48*, 9-18. https://doi.org/10.1037/cbs0000030

# Ensink, K., Normandin, L., Target, M., Fonagy, P., Sabourin, S., & Berthelot, N. (2015). Mentalization in children and mothers in the context of trauma: An initial study of the validity of the Child Reflective Functioning Scale. *British Journal of Developmental Psychology, 33*, 203–217. https://doi.org/10.1111/bjdp.12074

# Ensink, K., Rousseau, M. E., Biberdzic, M., Bégin, M., & Normandin, L. (2017). Reflective functioning and personality organization: Associations with negative maternal behaviors. *Infant Mental Health Journal, 38,* 351–362. https://doi.org/10.1002/imhj.21643

# Esbjørn, B. H., Pedersen, S. H., Daniel, S. I. F., Hald, H. H., Holm, J. M., & Steele, H. (2013). Anxiety levels in clinically referred children and their parents: Examining the unique influence of self-reported attachment styles and interview-based reflective functioning in mothers and fathers. *British Journal of Clinical Psychology, 52,* 394–407. https://doi.org/10.1111/bjc.12024

# Evans, A. P. B., Goodman, S. H., Dimidjian, S., & Gallop, R. (2019). The role of engagement in mindfulness-based cognitive therapy for the prevention of depressive relapse/recurrence in perinatal women. Mindfulness, *12*(1), 61-67. https://doi.org/10.1007/s12671-019-01160-4

# Evans, S., Bhide, S., Quek, J., Nicholson, J. M., Anderson, V., Hazell, P., Mulraney, M., & Sciberras, E. (2020). Mindful parenting behaviors and emotional self-regulation in children with ADHD and controls. *Journal of Pediatric Psychology, 45*(9), 1074–1083. https://doi.org/10.1093/jpepsy/jsaa073

# Fereydooni, A., Heidari, A., Saadi, Z. E., Ehteshamzadeh, P., & Pasha, R. (2020). A comparison of the effects of Happiness and Mindfulness Training on parenting self-efficacy in mothers of anxious preschool children. *International Journal of Pediatrics, 8*(11), 12327–12337. https://doi.org/10.22038/ijp.2020.48514.3906

# Fernandes, D. v., Canavarro, M. C., & Moreira, H. (2020a). Mindful parenting interventions for the postpartum period: Acceptance and preferences of mothers with and without depressive symptoms. *Mindfulness, 12*(2), 291–305. https://doi.org/10.1007/s12671-020-01430-6

# Fernandes, D. v., Canavarro, M. C., & Moreira, H. (2020b). The mediating role of parenting stress in the relationship between anxious and depressive symptomatology, mothers’ perception of infant temperament, and mindful parenting during the postpartum period. *Mindfulness, 12*(2), 275–290. https://doi.org/10.1007/s12671-020-01327-4

# Ferraioli, S. J., & Harris, S. L. (2013). Comparative effects of mindfulness and skills-based parent training programs for parents of children with autism: Feasibility and preliminary outcome data. *Mindfulness, 4*, 89–101. https://doi.org/10.1007/s12671-012-0099-0

# Fonagy, P., Sleed, M., & Baradon, T. (2016). Randomized controlled trial of parent-infant psychotherapy for parents with mental health problems and young infants. *Infant Mental Health Journal, 37,* 97–114. https://doi.org/10.1002/imhj.21553

# Gannon, M., MacKenzie, M., Kaltenbach, K., & Abatemarco, D. (2017). Impact of mindfulness-based parenting on women in treatment for opioid use disorder. *Journal of Addiction Medicine, 11*, 368–376. https://doi.org/10.1097/ADM.0000000000000336

# Georg, A., Kress, S., & Taubner, S. (2019). Strengthening mentalizing in a depressed mother of an infant with sleep disorders. *Journal of Clinical Psychology, 75*, 859-873.

# Gershy, N., & Gray, S. A. (2020). Parental emotion regulation and mentalization in families of children with ADHD. *Journal of Attention Disorders, 24,* 2084-2099.

# Gershy, N., Meehan, K. B., Omer, H., Papouchis, N., & Sapir, I. S. (2017). Randomized clinical trial of mindfulness skills augmentation in parent training. *Child and Youth Care Forum, 46,* 783–803. https://doi.org/10.1007/s10566-017-9411-4

# Geurtzen, N., Scholte, R. H. J., Engels, R. C. M. E., Tak, Y. R., & van Zundert, R. M. P. (2015). Association between mindful parenting and adolescents’ internalizing problems: Non-judgmental acceptance of parenting as core element. *Journal of Child and Family Studies, 24,* 1117–1128. https://doi.org/10.1007/s10826-014-9920-9

# Gheibi, Z., Abbaspour, Z., Haghighyzadeh, M. H., & Javadifar, N. (2020). Effects of a Mindfulness-Based Childbirth and Parenting program on maternal-fetal attachment: A randomized controlled trial among Iranian pregnant women. *Complementary Therapies in Clinical Practice, 41*. https://doi.org/10.1016/j.ctcp.2020.101226

# Goodman, J. H., Guarino, A., Chenausky, K., Klein, L., Prager, J., Petersen, R., … Freeman, M. (2014). CALM Pregnancy: Results of a pilot study of mindfulness-based cognitive therapy for perinatal anxiety. *Archives of Women’s Mental Health, 17*, 373–387. https://doi.org/10.1007/s00737-013-0402-7

# Gordo, L., Martinez-Pampliega, A., Elejalde, L. I., & Luyten, P. (2020). Do Parental Reflective Functioning and Parental Competence Affect The Socioemotional Adjustment Of Children?. *Journal of Child and Family Studies, 29*, 3621-3631.

# Gouveia, M. J. R., Canavarro, M. C. C. S. P., & Moreira, H. T. C. (2019). Linking mothers’ difficulties in emotion regulation to children/adolescents’ emotional eating in pediatric obesity: The mediating role of mindful parenting and children/adolescents’ depressive symptoms. *Mindfulness, 10*, 877–893. https://doi.org/10.1007/s12671-018-1055-4

# Gouveia, M. J. R., Canavarro, M. C. C. S. P., & Moreira, H. T. C. (2019). Linking mothers’ difficulties in emotion regulation to children/adolescents’ emotional eating in pediatric obesity: The mediating role of mindful parenting and children/adolescents’ depressive symptoms. *Mindfulness, 10*(5), 877–893. https://doi.org/10.1007/s12671-018-1055-4

# Gouveia, M. J., Canavarro, M. C., & Moreira, H. (2018). Is mindful parenting associated with adolescents’ emotional eating? The mediating role of adolescents’ self-compassion and body shame. *Frontiers in Psychology, 9¸*1-15*.* https://doi.org/10.3389/fpsyg.2018.02004

# Gouveia, M. J., Canavarro, M. C., & Moreira, H. (2018). The role of mindful parenting and children’s weight in mothers’ child-feeding practices. *Eating and Weight Disorders, 24*, 1-9. https://doi.org/10.1007/s40519-018-0615-x

# Gouveia, M. J., Carona, C., Canavarro, M. C., & Moreira, H. (2016). Self-compassion and dispositional mindfulness are associated with parenting styles and parenting stress: The mediating role of mindful parenting. *Mindfulness, 7,* 700–712. https://doi.org/10.1007/s12671-016-0507-y

# Grienenberger, J., Kelly, K., & Slade, A. (2005). Maternal reflective functioning, mother-infant affective communication, and infant attachment: Exploring the link between mental states and observed caregiving behavior in the intergenerational transmission of attachment. *Attachment and Human Development, 7,* 299–311. https://doi.org/10.1080/14616730500245963

# Guo, L., Zhang, J., Mu, L., & Ye, Z. (2020). Preventing postpartum depression with Mindful Self-Compassion Intervention: A randomized control study. *Journal of Nervous and Mental Disease, 208*(2), 101–107. https://doi.org/10.1097/NMD.0000000000001096

# Gurney-Smith, B., Downing, P., Kidd, K., & McMillin, R. (2017). ‘Minding the gap’: Developing mindfulness for adoption. *Adoption and Fostering, 41*, 110–119. https://doi.org/10.1177/0308575917702829

# Ha, C., Sharp, C., & Goodyer, I. (2011). The role of child and parental mentalizing for the development of conduct problems over time. *European Child and Adolescent Psychiatry, 20,* 291–300. https://doi.org/10.1007/s00787-011-0174-4

# Håkansson, U., Söderström, K., Watten, R., Skårderud, F., & Øie, M. G. (2018). Parental reflective functioning and executive functioning in mothers with substance use disorder. *Attachment and Human Development, 20*, 181–207. https://doi.org/10.1080/14616734.2017.1398764

# Håkansson, U., Watten, R. G., Söderström, K., & Øie, M. G. (2019). The association between executive functioning and parental stress and psychological distress is mediated by parental reflective functioning in mothers with substance use disorder. *Stress and Health, 35,* 1-14. https://doi.org/10.1002/smi.2868

# Håkansson, U., Watten, R., Söderström, K., Skårderud, F., & Øie, M. G. (2018). Adverse and adaptive childhood experiences are associated with parental reflective functioning in mothers with substance use disorder. *Child Abuse and Neglect, 81*, 259–273. https://doi.org/10.1016/j.chiabu.2018.05.007

# Halfon, S., & Besiroglu, B. (2020). Parental reflective function and children’s attachment-based mental state talk as predictors of outcome in psychodynamic child psychotherapy. Psychotherapy, 1-14.

# Han, Z. R., Ahemaitijiang, N., Yan, J., Hu, X., Parent, J., Dale, C., DiMarzio, K., Singh, N. N. (2019). Parent mindfulness, parenting, and child psychopathology in China. *Mindfulness*, *12*(2), 334-343. https://doi.org/10.1007/s12671-019-01111-z

# Handeland, T. B., Kristiansen, V. R., Lau, B., Håkansson, U., & Øie, M. G. (2019). High degree of uncertain reflective functioning in mothers with substance use disorder. *Addictive* *Behaviors Reports, 10*. Advance online publication. https://doi.org/10.1016/j.abrep.2019.100193

# Haydicky, J., Shecter, C., Wiener, J., & Ducharme, J. M. (2015). Evaluation of MBCT for adolescents with ADHD and their parents: Impact on individual and family functioning. *Journal of Child and Family Studies, 24,* 76–94. https://doi.org/10.1007/s10826-013-9815-1

# Heifetz, M., & Dyson, A. (2017). Mindfulness-based group for teens with developmental disabilities and their parents: A pilot study. *Mindfulness, 8,* 444–453. https://doi.org/10.1007/s12671-016-0616-7

Henrichs, J., van den Heuvel, M. I., Witteveen, A. B., Wilschut, J., & Van den Bergh, B. R. H. (2019). Does mindful parenting mediate the association between maternal anxiety during pregnancy and child behavioral/emotional problems? *Mindfulness, 12*(2), 370-380. https://doi.org/10.1007/s12671-019-01115-9.

# Heron-Delaney, M., Kenardy, J. A., Brown, E. A., Jardine, C., Bogossian, F., Neuman, L., … Pritchard, M. (2016). Early maternal reflective functioning and infant emotional regulation in a preterm infant sample at 6 months corrected age. *Journal of Pediatric Psychology, 41,* 906–914. https://doi.org/10.1093/jpepsy/jsv169

# Hertzmann, L., Target, M., Hewison, D., Casey, P., Fearon, P., & Lassri, D. (2016). Mentalization-based therapy for parents in entrenched conflict: A random allocation feasibility study. *Psychotherapy, 53*, 388–401. https://doi.org/10.1037/pst0000092

# Hicks, L. M., & Dayton, C. J. (2019). Mindfulness and trauma symptoms predict child abuse potential in risk-exposed, men and women during pregnancy. *Child Abuse and Neglect, 90,* 43–51. https://doi.org/10.1016/j.chiabu.2019.01.018

# Hicks, L. M., Dayton, C. J., & Victor, B. G. (2018). Depressive and trauma symptoms in expectant, risk-exposed, mothers and fathers: Is mindfulness a buffer? *Journal of Affective Disorders, 238,* 179–186. https://doi.org/10.1016/j.jad.2018.05.044

# Huber, A., McMahon, C., & Sweller, N. (2015). Improved child behavioural and emotional functioning after circle of security 20-week intervention. *Attachment and Human Development, 17*, 547–569. https://doi.org/10.1080/14616734.2015.1086395

# Huber, A., McMahon, C., & Sweller, N. (2016). Improved parental emotional functioning after circle of security 20-week parent–child relationship intervention. *Journal of Child and Family Studies, 25,* 2526–2540. https://doi.org/10.1007/s10826-016-0426-5

# Hunter, J. E., Jenkins, C. L., Grim, V., Leung, S., Charen, K. H., Hamilton, D. R., Allen, E. G., & Sherman, S. L. (2019). Feasibility of an app-based mindfulness intervention among women with an FMR1 premutation experiencing maternal stress. *Research in Developmental Disabilities, 89,* 76–82. https://doi.org/10.1016/j.ridd.2019.03.008

# Huth-Bocks, A. C., Muzik, M., Beeghly, M., Earls, L., & Stacks, A. M. (2014). Secure base scripts are associated with maternal parenting behavior across contexts and reflective functioning among trauma-exposed mothers. *Attachment and Human Development, 16,* 535–556. https://doi.org/10.1080/14616734.2014.967787

# Hwang, Y. S., Kearney, P., Klieve, H., Lang, W., & Roberts, J. (2015). Cultivating mind: Mindfulness interventions for children with autism spectrum disorder and problem behaviours, and their mothers. *Journal of Child and Family Studies, 24,* 3093–3106. https://doi.org/10.1007/s10826-015-0114-x

# Jastreboff, A. M., Chaplin, T. M., Finnie, S., Savoye, M., Stults-Kolehmainen, M., Silverman, W. K., & Sinha, R. (2018). Preventing childhood obesity through a mindfulness-based parent stress intervention: A randomized pilot study. *Journal of Pediatrics, 202,* 136-142.e1. https://doi.org/10.1016/j.jpeds.2018.07.011

# Jessee, A., Mangelsdorf, S. C., Wong, M. S., Schoppe-Sullivan, S. J., Shigeto, A., & Brown, G. L. (2018). The role of reflective functioning in predicting marital and coparenting quality. *Journal of Child and Family Studies, 27,* 187–197. https://doi.org/10.1007/s10826-017-0874-6

# Jones, L., Gold, E., Totsika, V., Hastings, R. P., Jones, M., Griffiths, A., & Silverton, S. (2018). A mindfulness parent well-being course: Evaluation of outcomes for parents of children with autism and related disabilities recruited through special schools. *European Journal of Special Needs Education, 33, 16*–30. https://doi.org/10.1080/08856257.2017.1297571

# Jones, L., Hastings, R. P., Totsika, V., Keane, L., & Rhule, N. (2014). Child behavior problems and parental well-being in families of children with autism: The mediating role of mindfulness and acceptance. *American Journal on Intellectual and Developmental Disabilities, 119*, 171–185. https://doi.org/10.1352/1944-7558-119.2.171

# Kil, H., & Grusec, J. E. (2020). Links among mothers’ dispositional mindfulness, stress, perspective-taking, and mother-child interactions. *Mindfulness, 11*(7), 1710–1722. https://doi.org/10.1007/s12671-020-01387-6

# Kohlhoff, J., Stein, M., Ha, M., & Mejaha, K. (2016). The Circle of Security Parenting (COS-P) intervention: Pilot evaluation. *Australian Journal of Child and Family Health Nursing, 13,* 3-7.

# Korukcu, O., & Kukulu, K. (2017). The effect of the mindfulness-based transition to motherhood program in pregnant women with preterm premature rupture of membranes. *Health Care for Women International, 38,* 765–785. https://doi.org/10.1080/07399332.2017.1318882

# Krink, S., Muehlhan, C., Luyten, P., Romer, G., & Ramsauer, B. (2018). Parental reflective functioning affects sensitivity to distress in mothers with postpartum depression. *Journal of Child and Family Studies, 27,* 1671–1681. https://doi.org/10.1007/s10826-017-1000-5

# Laurent, H. K., Duncan, L. G., Lightcap, A., & Khan, F. (2017). Mindful parenting predicts mothers’ and infants’ hypothalamic-pituitary-adrenal activity during a dyadic stressor. *Developmental Psychology, 53,* 417–424. https://doi.org/10.1037/dev0000258

# Laurent, H. K., Wright, D., & Finnegan, M. (2018). Mindfulness-related differences in neural response to own infant negative versus positive emotion contexts. *Developmental Cognitive Neuroscience, 30, 70*–76. https://doi.org/10.1016/j.dcn.2018.01.002

Lengua, L. J., Ruberry, E. J., McEntire, C., Klein, M., & Jones, B. (2018). Preliminary evaluation of an innovative, brief parenting program designed to promote self-regulation in parents and children. *Mindfulness, 12*(2), 438-449. https://doi.org/10.1007/s12671-018-1016-y.

# León, E., Palacios, J., Román, M., Moreno, C., & Peñarrubia, M. G. (2015). Parental stress, family functioning and children’s psychological adjustment in adoptive families: A comparative and longitudinal study. *Family Science, 6,* 50–57. https://doi.org/10.1080/19424620.2015.1080991

# León, E., Steele, M., Palacios, J., Román, M., & Moreno, C. (2018). Parenting adoptive children: Reflective functioning and parent-child interactions. A comparative, relational and predictive study. *Children and Youth Services Review, 95,* 352–360. https://doi.org/10.1016/j.childyouth.2018.11.009

# León, M. J., & Olhaberry, M. (2020). Triadic interactions, parental reflective functioning, and early social‐emotional difficulties. *Infant Mental Health Journal*, 1-11. https://doi.org/10.1002/imhj.21844

# Letourneau, N., Anis, L., Ntanda, H., Novick, J., Steele, M., Steele, H., & Hart, M. (2020). Attachment & Child Health (ATTACH) pilot trials: Effect of parental reflective function intervention for families affected by toxic stress. *Infant Mental Health Journal*, 1-18.

# Lewallen, A. C., & Neece, C. L. (2015). Improved social skills in children with developmental delays after parent participation in MBSR: The role of parent–child relational factors. *Journal of Child and Family Studies, 24*, 3117–3129. https://doi.org/10.1007/s10826-015-0116-8

# Lippold, M. A., Duncan, L. G., Coatsworth, J. D., Nix, R. L., & Greenberg, M. T. (2015). Understanding how mindful parenting may be linked to mother–adolescent communication. *Journal of Youth and Adolescence, 44*, 1663–1673. https://doi.org/10.1007/s10964-015-0325-x

Lippold, M. A., Jensen, T. M., Duncan, L. G., Nix, R. L., Coatsworth, J. D., & Greenberg, M. T. (2019). Mindful parenting, parenting cognitions, and parent-youth communication: Bidirectional linkages and mediational processes. *Mindfulness, 12*(2), 381-391. https://doi.org/10.1007/s12671-019-01119-5.

# Liu, Z., Sun, X., Guo, Y., & Luo, F. (2019). Mindful parenting inhibits adolescents from being greedy: The mediating role of adolescent core self-evaluations. *Current Psychology.* https://doi.org/10.1007/s12144-019-00577-3

# Ljubetić, M., & Ercegovac, I. R. (2020). The relationship between mindful parenting, cognitive parental awareness, and the subjective well-being of adolescents. *Metodički Ogledi: Časopis Za Filozofiju Odgoja, 27*(1), 103–126.

# Lloyd, T., & Hastings, R. P. (2008). Psychological variables as correlates of adjustment in mothers of children with intellectual disabilities: Cross-sectional and longitudinal relationships. *Journal of Intellectual Disability Research, 52*, 37–48. https://doi.org/10.1111/j.1365-2788.2007.00974.x

# Lo, H. H. M., Chan, S. K. C., Szeto, M. P., Chan, C. Y. H., & Choi, C. W. (2017). A feasibility study of a brief mindfulness-based program for parents of preschool children with developmental disabilities. *Mindfulness, 8,* 1665–1673. https://doi.org/10.1007/s12671-017-0741-y

# Lo, H. H. M., Wong, S. W. L., Wong, J. Y. H., Yeung, J. W. K., Snel, E., & Wong, S. Y. S. (2020). The effects of Family-Based Mindfulness Intervention on ADHD symptomology in young children and their parents: A randomized control trial. *Journal of Attention Disorders, 24(*5), 667–680. https://doi.org/10.1177/1087054717743330

# Lo, Herman H.M., Wong, S. W. L., Wong, J. Y. H., Yeung, J. W. K., Snel, E., & Wong, S. Y. S. (2017). The effects of family-based mindfulness intervention on ADHD symptomology in young children and their parents: A randomized control trial. *Journal of Attention Disorders, 24*(5), 667-680. https://doi.org/10.1177/1087054717743330

# Lönnberg, G., Jonas, W., Bränström, R., Nissen, E., & Niemi, M. (2020). Long-term effects of a Mindfulness-Based Childbirth and Parenting Program— A randomized controlled trial. *Mindfulness, 12*(2), 476–488. https://doi.org/10.1007/s12671-020-01403-9

# Lönnberg, G., Jonas, W., Unternaehrer, E., Bränström, R., Nissen, E., & Niemi, M. (2020). Effects of a Mindfulness Based Childbirth and Parenting program on pregnant women’s perceived stress and risk of perinatal depression–Results from a randomized controlled trial. *Journal of Affective Disorders, 262,* 133–142. https://doi.org/10.1016/j.jad.2019.10.048

# Lunsky, Y., Hastings, R. P., Weiss, J. A., Palucka, A. M., Hutton, S., & White, K. (2017). Comparative effects of mindfulness and support and information group interventions for parents of adults with autism spectrum disorder and other developmental disabilities. *Journal of Autism and Developmental Disorders, 47*, 1769–1779. https://doi.org/10.1007/s10803-017-3099-z

# Lunsky, Y., Robinson, S., Reid, M., & Palucka, A. (2015). Development of a mindfulness-based coping with stress group for parents of adolescents and adults with developmental disabilities. *Mindfulness, 6,* 1335–1344. https://doi.org/10.1007/s12671-015-0404-9

# MacDonald, E. E., & Hastings, R. P. (2010). Mindful parenting and care involvement of fathers of children with intellectual disabilities. *Journal of Child and Family Studies, 19,* 236–240. https://doi.org/10.1007/s10826-008-9243-9

# Maglica, T., Ercegovac, I. R., & Ljubetic, M. (2020). Mindful parenting and behavioural problems in preschool children. *Hrvatska Revija Za Rehabilitacijska Istrazivanja, 56*(1), 44–58.

# Mah, J. W. T., Murray, C., Locke, J., & Carbert, N. (2020). Mindfulness-Enhanced Behavioral Parent Training for clinic-referred families of children with ADHD: A randomized controlled trial. *Journal of Attention Disorders, 25(*12). https://doi.org/10.1177/1087054720925882

# Maloney, R., & Altmaier, E. (2007). An initial evaluation of a mindful parenting program. *Journal of Clinical Psychology, 63,* 1231–1238. https://doi.org/10.1002/jclp.20395

# Mann, J., Kuyken, W., O’Mahen, H., Ukoumunne, O. C., Evans, A., & Ford, T. (2016). Manual development and pilot randomised controlled trial of mindfulness-based cognitive therapy versus usual care for parents with a history of depression. *Mindfulness, 7,* 1024–1033. https://doi.org/10.1007/s12671-016-0543-7

# Mata López, C., Santelices Álvarez, M. P., & Vergés Gómez, A. (2020). Do educators matter? Associations between caregivers’ mentalization and preschoolers’ attachment, social emotional development and theory of mind. *Early Child Development and Care*, 1-15. https://doi.org/10.1080/03004430.2020.1755664

# Maughan, A. L., & Weiss, J. A. (2017). Parental outcomes following participation in cognitive behavior therapy for children with autism spectrum disorder*. Journal of Autism and Developmental Disorders, 47,* 3166–3179. https://doi.org/10.1007/s10803-017-3224-z

# Maupin, A. N., Samuel, E. E., Nappi, S. M., Heath, J. M., & Smith, M. V. (2017). Disseminating a parenting intervention in the community: Experiences from a multi-site evaluation. *Journal of Child and Family Studies, 26*, 3079–3092. https://doi.org/10.1007/s10826-017-0804-7

# May, L. M., Reinka, M. A., Tipsord, J. M., Felver, J. C., & Berkman, E. T. (2016). Parenting an early adolescent: A pilot study examining neural and relationship quality changes of a mindfulness intervention. *Mindfulness, 7,* 1203–1213. https://doi.org/10.1007/s12671-016-0563-3

# McGregor, H. A., Sanner, C. M., & Neece, C. L. (2020). Effects of MBSR Parent Intervention on internalizing problems in children: ASD status as a moderator. *Journal of Mental Health Research in Intellectual Disabilities, 13*(4), 343–363. https://doi.org/10.1080/19315864.2020.1815913

# McKee, L. G., Parent, J., Zachary, C. R., & Forehand, R. (2018). Mindful parenting and emotion socialization practices: Concurrent and longitudinal associations. *Family Process, 57*, 752–766. https://doi.org/10.1111/famp.12329

# Meamar, E., Meamar, E., Keshavarzi, F., Emamipour, S., & Golshani, F. (2015). Effectiveness of mindful parenting training on mothers’ affective self-regulation and on the externalizing behavioral problems in adolescent girls. *Applied Environmental and Biological Sciences. 5,* 677–682.

# Medeiros, C., Gouveia, M. J., Canavarro, M. C., & Moreira, H. (2016). The indirect effect of the mindful parenting of mothers and fathers on the child’s perceived well-being through the child’s attachment to parents. *Mindfulness, 7,* 916–927. https://doi.org/10.1007/s12671-016-0530-z

# Meppelink, R., de Bruin, E. I., Wanders-Mulder, F. H., Vennik, C. J., & Bögels, S. M. (2016). Mindful parenting training in child psychiatric settings: Heightened parental mindfulness reduces parents’ and children’s psychopathology. *Mindfulness, 7*, 680–689. https://doi.org/10.1007/s12671-016-0504-1

# Midgley, N., Cirasola, A., Austerberry, C., Ranzato, E., West, G., Martin, P., … Park, T. (2019). Supporting foster carers to meet the needs of looked after children: A feasibility and pilot evaluation of the reflective fostering programme. *Developmental Child Welfare, 1*, 41–60. https://doi.org/10.1177/2516103218817550

# Miklósi, M., Szabó, M., & Simon, L. (2017). The role of mindfulness in the relationship between perceived parenting, early maladaptive schemata and parental sense of competence. *Mindfulness, 8,* 471–480. https://doi.org/10.1007/s12671-016-0619-4

# Minor, H. G., Carlson, L. E., Mackenzie, M. J., Zernicke, K., & Jones, L. (2006). Evaluation of a mindfulness-based stress reduction (MBSR) program for caregivers of children with chronic conditions. *Social Work in Health Care, 43,* 91–109. https://doi.org/10.1300/J010v43n01_06

# Mohammadi, F. S., Chorami, M., Sharifi, T., & Ghazanfari, A. (2020). Comparing the effects of group training of mindful parenting skills and psychological capital on stress and psychological flexibility in mothers with blind girl students. *International Journal of School Health, 7*(3), 31–38.

# Mohaupt, H., & Duckert, F. (2016). Parental reflective functioning in fathers who use intimate partner violence: Findings from a Norwegian clinical sample. *Nordic Psychology, 68,* 272–286. https://doi.org/10.1080/19012276.2016.1162107

# Möller, C., Odersjö, C., Pilesjö, F., Terpening, K., Österberg, M., & Holmqvist, R. (2017). Reflective functioning, limit setting, and emotional availability in mother–child dyads. *Parenting, 17*, 225–241. https://doi.org/10.1080/15295192.2017.1369311

# Moreira, H., & Canavarro, M. C. (2015). Individual and gender differences in mindful parenting: The role of attachment and caregiving representations. *Personality and Individual Differences, 87*, 13–19. https://doi.org/10.1016/j.paid.2015.07.021

# Moreira, H., & Canavarro, M. C. (2018a). Does the association between mindful parenting and adolescents’ dispositional mindfulness depend on the levels of anxiety and depression symptomatology in mothers? *Journal of Adolescence, 68*, 22–31. https://doi.org/10.1016/j.adolescence.2018.07.003

# Moreira, H., & Canavarro, M. C. (2018b). The association between self-critical rumination and parenting stress: The mediating role of mindful parenting. *Journal of Child and Family Studies, 27,* 2265–2275. https://doi.org/10.1007/s10826-018-1072-x

# Moreira, H., & Canavarro, M. C. (2020). Mindful parenting is associated with adolescents’ difficulties in emotion regulation through adolescents’ psychological inflexibility and self-compassion. *Journal of Youth and Adolescence, 49*(1), 192–211. https://doi.org/10.1007/s10964-019-01133-9

# Moreira, H., Caiado, B., & Canavarro, M. C. (2020). Is mindful parenting a mechanism that links parents’ and children’s tendency to experience negative affect to overprotective and supportive behaviors? *Mindfulness, 12*(2), 319–333. https://doi.org/10.1007/s12671-020-01468-6

# Moreira, H., Carona, C., Silva, N., Nunes, J., & Canavarro, M. C. (2016). Exploring the link between maternal attachment-related anxiety and avoidance and mindful parenting: The mediating role of self-compassion. *Psychology and Psychotherapy: Theory, Research and Practice, 89,* 369–384. https://doi.org/10.1111/papt.12082

# Moreira, H., Fonseca, A., Caiado, B., & Canavarro, M. C. (2019). Work-family conflict and mindful parenting: The mediating role of parental psychopathology symptoms and parenting stress in a sample of Portuguese employed parents. *Frontiers in Psychology, 10.* https://doi.org/10.3389/fpsyg.2019.00635

# Moreira, H., Gouveia, M. J., & Canavarro, M. C. (2018). Is mindful parenting associated with adolescents’ well-being in early and middle/late adolescence? The mediating role of adolescents’ attachment representations, self-compassion and mindfulness. *Journal of Youth and Adolescence, 47,* 1771–1788. https://doi.org/10.1007/s10964-018-0808-7

# Moser, D. A., Suardi, F., Rossignol, A. S., Vital, M., Manini, A., Serpa, S. R., & Schechter, D. S. (2019). Parental Reflective Functioning correlates to brain activation in response to video-stimuli of mother–child dyads: Links to maternal trauma history and PTSD. *Psychiatry Research: Neuroimaging, 293*, 110985. https://doi.org/10.1016/j.pscychresns.2019.09.005

# Neece, C. L. (2014). Mindfulness-based stress reduction for parents of young children with developmental delays: Implications for parental mental health and child behavior problems. *Journal of Applied Research in Intellectual Disabilities, 27*, 174–186. https://doi.org/10.1111/jar.12064

# Neece, C. L., Chan, N., Klein, K., Roberts, L., & Fenning, R. M. (2019). Mindfulness-based stress reduction for parents of children with developmental delays: Understanding the experiences of Latino families. *Mindfulness, 10,* 1017–1030. https://doi.org/10.1007/s12671-018-1011-3

# Nguyen, T. M., Bui, T. T. H., Xiao, X., & Le, V. H. (2020). The influence of self-compassion on mindful parenting: A mediation model of gratitude*. Family Journal, 28*(4), 455–462. https://doi.org/10.1177/1066480720950421

# Nijssens, L., Bleys, D., Casalin, S., Vliegen, N., & Luyten, P. (2018). Parental attachment dimensions and parenting stress: The mediating role of parental reflective functioning. *Journal of Child and Family Studies, 27*, 2025–2036. https://doi.org/10.1007/s10826-018-1029-0

# Ordway, M. R., Sadler, L. S., Dixon, J., Close, N., Mayes, L., & Slade, A. (2014). Lasting effects of an interdisciplinary home visiting program on child behavior: Preliminary follow-up results of a randomized trial. *Journal of Pediatric Nursing, 29*, 3–13. https://doi.org/10.1016/j.pedn.2013.04.006

# Pajulo, M., Pyykkönen, N., Kalland, M., Sinkkonen, J., Helenius, H., Punamäki, R. L., & Suchman, N. (2012). Substance-abusing mothers in residential treatment with their babies: Importance of pre- and postnatal maternal reflective functioning. *Infant Mental Health Journal, 33,* 70–81. https://doi.org/10.1002/imhj.20342

# Pajulo, M., Suchman, N., Kalland, M., Sinkkonen, J., Helenius, H., & Mayes, L. (2008). Role of maternal reflective ability for substance abusing mothers. *Journal of Prenatal & Perinatal Psychology & Health, 23,* 13–31.

# Pan, W. L., Gau, M. L., Lee, T. Y., Jou, H. J., Liu, C. Y., & Wen, T. K. (2019). Mindfulness-based programme on the psychological health of pregnant women. *Women and Birth, 32,* e102–e109. https://doi.org/10.1016/j.wombi.2018.04.018

# Pan, W.-L., Chang, C.-W., Chen, S.-M., & Gau, M.-L. (2019). Assessing the effectiveness of mindfulness-based programs on mental health during pregnancy and early motherhood - A randomized control trial. *BMC Pregnancy and Childbirth, 19*(1). https://doi.org/10.1186/s12884-019-2503-4

# Parent, J., Clifton, J., Forehand, R., Golub, A., Reid, M., & Pichler, E. R. (2014). Parental mindfulness and dyadic relationship quality in low-income cohabiting Black stepfamilies: Associations with parenting experienced by adolescents. *Couple and Family Psychology: Research and Practice, 3,* 67–82. https://doi.org/10.1037/cfp0000020

# Parent, J., Dale, C. F., McKee, L. G., & Sullivan, A. D. W. (2020). The longitudinal influence of caregiver dispositional mindful attention on mindful parenting, parenting practices, and youth psychopathology. *Mindfulness, 12*(2), 357–369. https://doi.org/10.1007/s12671-020-01536-x

# Parent, J., Garai, E., Forehand, R., Roland, E., Potts, J., Haker, K., … Compas, B. E. (2011). Parent mindfulness and child outcome: The roles of parent depressive symptoms and parenting. *Mindfulness, 1*, 254–264. https://doi.org/10.1007/s12671-010-0034-1

# Parent, J., McKee, L. G., Anton, M., Gonzalez, M., Jones, D. J., & Forehand, R. (2016). Mindfulness in parenting and coparenting. *Mindfulness, 7,* 504–513. https://doi.org/10.1007/s12671-015-0485-5

# Parent, J., McKee, L. G., N. Rough, J., & Forehand, R. (2016). The association of parent mindfulness with parenting and youth psychopathology across three developmental stages. *Journal of Abnormal Child Psychology, 44*, 191–202. https://doi.org/10.1007/s10802-015-9978-x

# Paris, R., Herriott, A., Holt, M., & Gould, K. (2015). Differential responsiveness to a parenting intervention for mothers in substance abuse treatment. *Child Abuse and Neglect, 50,* 206–217. https://doi.org/10.1016/j.chiabu.2015.09.007

# Park, Y. Nix, R., Duncan, L. G., Coatsworth, J. D., Greenberg, M. T. (2020). Unfolding relations among mother' mindful parenting, recurrent conflict, and adolescents' externalizing and internalizing problems. *Family Process,* *59*(4), 1690-1705.

# Pazzagli, C., Germani, A., Buratta, L., Luyten, P., & Mazzeschi, C. (2019). Childhood obesity and parental reflective functioning: Is there a relation?. *International Journal of Clinical and Health Psychology, 19,* 209-217.

# Perez-Blasco, J., Viguer, P., & Rodrigo, M. F. (2013). Effects of a mindfulness-based intervention on psychological distress, well-being, and maternal self-efficacy in breast-feeding mothers: Results of a pilot study. *Archives of Women’s Mental Health, 16,* 227–236. https://doi.org/10.1007/s00737-013-0337-z

# Poormirzaei, M., & Bagheri, M. (2020). The mediating role of child’s cognitive emotion regulation in the relationship between parental mindfulness and child’s mind reading ability. *Cognitive Processing, 21*(3), 403–410. https://doi.org/10.1007/s10339-020-00951-1

# Potharst, E. S., Aktar, E., Rexwinkel, M., Rigterink, M., & Bögels, S. M. (2017). Mindful with Your Baby: Feasibility, acceptability, and effects of a mindful parenting group training for mothers and their babies in a mental health context. *Mindfulness, 8,* 1236–1250. https://doi.org/10.1007/s12671-017-0699-9

# Potharst, E. S., Baartmans, J. M. D., & Bögels, S. M. (2018). Mindful parenting training in a clinical versus non-clinical setting: An explorative study. Mindfulness. https://doi.org/10.1007/s12671-018-1021-1

# Potharst, E. S., Boekhorst, M. G. B. M., Cuijlits, I., van Broekhoven, K. E. M., Jacobs, A., Spek, V., … Pop, V. J. M. (2019). A randomized control trial evaluating an online mindful parenting training for mothers with elevated parental stress. Frontiers in Psychology, 10. https://doi.org/10.3389/fpsyg.2019.01550

# Potharst, E. S., Leyland, A., Colonnesi, C., Veringa, I. K., Salvadori, E. A., Jakschik, M., Bögels, S. M., & Zeegers, M. A. J. (2020). Does mothers’ self-reported mindful parenting relate to the observed quality of parenting behavior and mother-child interaction? *Mindfulness, 12*(2), 344–356. https://doi.org/10.1007/s12671-020-01533-0

# Potharst, E. S., Zeegers, M., & Bögels, S. M. (2018). Mindful with Your Toddler group training: feasibility, acceptability, and effects on subjective and objective measures. *Mindfulness, 9,* 1-15. https://doi.org/10.1007/s12671-018-1073-2

# Price, C., Kantrowitz-Gordon, I., & Calhoun, R. (2019). A pilot feasibility study of mindfulness childbirth education for women with a history of sexual trauma*. Complementary Therapies in Clinical Practice, 37,* 102–108. https://doi.org/10.1016/j.ctcp.2019.09.005

# Pugsley, L., & Acar, S. (2020). Supporting creativity or conformity? Influence of home environment and parental factors on the value of children’s creativity characteristics. *Journal of Creative Behavior, 54*(3), 598–609. https://doi.org/10.1002/jocb.393

# Raulston, T. J., Zemantic, P. K., Machalicek, W., Hieneman, M., Kurtz-Nelson, E., Barton, H., Hansen, S. G., & Frantz, R. J. (2019). Effects of a brief Mindfulness-Infused Behavioral Parent training for mothers of children with Autism Spectrum Disorder. *Journal of Contextual Behavioral Science, 13,* 42–51. https://doi.org/10.1016/j.jcbs.2019.05.001

# Rayan, A., & Ahmad, M. (2016). Effectiveness of mindfulness-based interventions on quality of life and positive reappraisal coping among parents of children with Autism Spectrum Disorder. *Research in Developmental Disabilities, 55*, 185–196. https://doi.org/10.1016/j.ridd.2016.04.002

# Rayan, A., & Ahmad, M. (2017). Effectiveness of mindfulness-based intervention on perceived stress, anxiety, and depression among parents of children with Autism Spectrum Disorder. *Mindfulness, 8,* 677–690. https://doi.org/10.1007/s12671-016-0595-8

# Ren, Y., Han, Z. R., Ahemaitijiang, N., & Zhang, G. (2020). Maternal mindfulness and school-age children’s emotion regulation: Mediation by positive parenting practices and moderation by maternal perceived life stress. Mindfulness, 12(2), 306–318. https://doi.org/10.1007/s12671-019-01300-w

# Ren, Y., Hu, X., Han, Z. R., Yang, X., & Li, M. (2020). Mindful parenting and parenting practices in Chinese families of children with Autism Spectrum Disorder. *Journal of Child and Family Studies, 29*(2), 559–571. https://doi.org/10.1007/s10826-019-01549-8

# Rice, R., Ni Bhearra, A., Kilbride, K., Lynch, C., & McNicholas, F. (2020). Rolling out a Mindfulness-Based Stress Reduction intervention for parents of children with ADHD: A feasibility study. *Irish Journal of Psychological Medicine.* https://doi.org/10.1017/ipm.2020.121

# Ridderinkhof, A., de Bruin, E. I., Blom, R., & Bögels, S. M. (2018). Mindfulness-based program for children with autism spectrum disorder and their parents: Direct and long-term improvements. *Mindfulness, 9,* 773–791. https://doi.org/10.1007/s12671-017-0815-x

# Riva Crugnola, C., Ierardi, E., & Canevini, M. P. (2018). Reflective functioning, maternal attachment, mind-mindedness, and emotional availability in adolescent and adult mothers at infant 3 months. *Attachment and Human Development, 20,* 84–106. https://doi.org/10.1080/14616734.2017.1379546

# Riva Crugnola, C., Ierardi, E., Bottini, M., Verganti, C., & Albizzati, A. (2019). Childhood experiences of maltreatment, reflective functioning and attachment in adolescent and young adult mothers: Effects on mother-infant interaction and emotion regulation. *Child Abuse and Neglect, 93*, 277–290. https://doi.org/10.1016/j.chiabu.2019.03.024

# Roach, A. T., Mhende, J., Barger, B., & Roberts, D. A. (2019). Implementing mindfulness practices with parents of young children in a low-socioeconomic status neighborhood. *Journal of the Georgia Public Health Association, 7*(2), 49–58. https://doi.org/10.20429/jgpha.2019.070206

# Roberts, L. R., & Neece, C. L. (2015). Feasibility of mindfulness-based stress reduction intervention for parents of children with developmental delays*. Issues in Mental Health Nursing, 36*, 592–602. https://doi.org/10.3109/01612840.2015.1017063

# Roberts, L. R., Boostrom, G. G., Dehom, S. O., & Neece, C. L. (2020). Self-reported parenting stress and cortisol awakening response following Mindfulness-Based Stress Reduction intervention for parents of children with Developmental Delays: A pilot study. *Biological Research for Nursing, 22*(2), 217–225. https://doi.org/10.1177/1099800419890125

# Røhder, K., Væver, M. S., Aarestrup, A. K., Jacobsen, R. K., Smith-Nielsen, J., & Schiøtz, M. L. (2020). Maternal-fetal bonding among pregnant women at psychosocial risk: The roles of adult attachment style, prenatal parental reflective functioning, and depressive symptoms. *PloS One*, *15*, 1-19. https://doi.org/10.1371/journal.pone.0239208

# Rosenblum, K. L., McDonough, S. C., Sameroff, A. J., & Muzik, M. (2008). Reflection in thought and action: Maternal parenting reflectivity predicts mind-minded comments and interactive behavior. *Infant Mental Health Journal, 29,* 362–376. https://doi.org/10.1002/imhj.20184

# Rosso, Anna M., & Airaldi, C. (2016). Intergenerational transmission of reflective functioning. Frontiers in Psychology, 7. https://doi.org/10.3389/fpsyg.2016.01903

# Rosso, Anna Maria, Viterbori, P., & Scopesi, A. M. (2015). Are maternal reflective functioning and attachment security associated with preadolescent mentalization? *Frontiers in Psychology, 6*. https://doi.org/10.3389/fpsyg.2015.01134

# Rostad, W. L., & Whitaker, D. J. (2016). The association between reflective functioning and parent–child relationship quality. *Journal of Child and Family Studies, 25*, 2164–2177. https://doi.org/10.1007/s10826-016-0388-7

# Ruiz, N., Witting, A., Ahnert, L., & Piskernik, B. (2019). Reflective functioning in fathers with young children born preterm and at term. *Attachment and Human Development*, *22*(1), 32-45. https://doi.org/10.1080/14616734.2019.1589059

# Ruskin, D., Campbell, L., Stinson, J., & Ahola Kohut, S. (2018). Changes in parent psychological flexibility after a one-time mindfulness-based intervention for parents of adolescents with persistent pain conditions. *Children, 5*, 121-XX. https://doi.org/10.3390/children5090121

# Rutherford, H. J. V., Booth, C. R., Luyten, P., Bridgett, D. J., & Mayes, L. C. (2015). Investigating the association between parental reflective functioning and distress tolerance in motherhood. *Infant Behavior and Development, 40,* 54–63. https://doi.org/10.1016/j.infbeh.2015.04.005

# Rutherford, H. J. V., Byrne, S. P., Crowley, M. J., Bornstein, J., Bridgett, D. J., & Mayes, L. C. (2018). Executive functioning predicts reflective functioning in mothers*. Journal of Child and Family Studies, 27,* 944–952. https://doi.org/10.1007/s10826-017-0928-9

# Rutherford, H. J. V., Crowley, M. J., Gao, L., Francis, B., Schultheis, A., & Mayes, L. C. (2018). Prenatal neural responses to infant faces predict postpartum reflective functioning. *Infant Behavior and Development, 53,* 43–48. https://doi.org/10.1016/j.infbeh.2018.09.003

# Rutherford, H. J. V., Goldberg, B., Luyten, P., Bridgett, D. J., & Mayes, L. C. (2013). Parental reflective functioning is associated with tolerance of infant distress but not general distress: Evidence for a specific relationship using a simulated baby paradigm*. Infant Behavior and Development, 36*, 635–641. https://doi.org/10.1016/j.infbeh.2013.06.008

# Rutherford, H. J. V., Maupin, A. N., Landi, N., Potenza, M. N., & Mayes, L. C. (2017). Parental reflective functioning and the neural correlates of processing infant affective cues. *Social Neuroscience, 12,* 519–529. https://doi.org/10.1080/17470919.2016.1193559

# Sadler, L. S., Slade, A., Close, N., Webb, D. L., Simpson, T., Fennie, K., & Mayes, L. C. (2013). Minding the baby: Enhancing reflectiveness to improve early health and relationship outcomes in an interdisciplinary home-visiting program. *Infant Mental Health Journal, 34,* 391–405. https://doi.org/10.1002/imhj.21406

# Salem-Guirgis, S., Albaum, C., Tablon, P., Riosa, P. B., Nicholas, D. B., Drmic, I. E., & Weiss, J. A. (2019). MYmind: A concurrent group-based mindfulness intervention for youth with autism and their parents. *Mindfulness*, *10*(9), 1730-1743. https://doi.org/10.1007/s12671-019-01107-9

# Salo, S. J., Flykt, M., Mäkelä, J., Biringen, Z., Kalland, M., Pajulo, M., & Punamäki, R. L. (2019). The effectiveness of Nurture and Play: a mentalisation-based parenting group intervention for prenatally depressed mothers. *Primary Health Care Research & Development, 20,* 1-11.

Schechter, D. S., Coates, S. W., Kaminer, T., Coots, T., Zeanah, C. H., Davies, M., Schonfeld, I.S. Marhsall, R.D., Liebowitz, M. R., Trabka, K.A., McCaw BA, J.E., & Myers, M. M. (2008). Distorted maternal mental representations and atypical behavior in a clinical sample of violence-exposed mothers and their toddlers. *Journal of Trauma and Dissociation*, *9*(2), 123–147. https://doi.org/10.1080/15299730802045666

# Schechter, D. S., Coots, T., Zeanah, C. H., Davies, M., Coates, S. W., Trabka, K. A., Marshall, R.D., Liebowitz, M.R., Myers, M. M. (2005). Maternal mental representations of the child in an inner-city clinical sample: Violence-related posttraumatic stress and reflective functioning. *Attachment and Human Development, 7,* 313–331. https://doi.org/10.1080/14616730500246011

# Schultheis, A. M., Mayes, L. C., & Rutherford, H. J. V. (2019). Associations between emotion regulation and parental reflective functioning. *Journal of Child and Family Studies, 28,* 1094–1104. https://doi.org/10.1007/s10826-018-01326-z

# Scopesi, A. M., Rosso, A. M., Viterbori, P., & Panchieri, E. (2015). Mentalizing abilities in preadolescents’ and their mothers’ autobiographical narratives. *Journal of Early Adolescence, 35,* 467–483. https://doi.org/10.1177/0272431614535091

# Sealy, J., & Glovinsky, I. P. (2016). Strengthening the reflective functioning capacities of parents who have a child with a neurodevelopmental disability through a brief, relationship-focused intervention. *Infant Mental Health Journal, 37,* 115–124. https://doi.org/10.1002/imhj.21557

# Seidman, L. C., Martin, S. R., Trant, M. W., Payne, L. A., Zeltzer, L. K., Cousineau, T. M., & Donovan, E. (2019). Feasibility and acceptance testing of a mobile application providing psychosocial support for parents of children and adolescents with chronic pain: Results of a nonrandomized trial. *Journal of Pediatric Psychology, 44,* 645–655. https://doi.org/10.1093/jpep/jsz007

# Serkel-Schrama, I. J. P., de Vries, J., Nieuwesteeg, A. M., Pouwer, F., Nyklíček, I., Speight, J., … Hartman, E. E. (2016). The association of mindful parenting with glycemic control and quality of life in adolescents with Type 1 Diabetes: Results from diabetes MILES—The Netherlands. *Mindfulness, 7,* 1227–1237. https://doi.org/10.1007/s12671-016-0565-1

# Shaffer, E. J., Lape, J. E., & Salls, J. (2020). Decreasing stress for parents of special needs children through a Web-Based Mindfulness Program: A pilot study. *Internet Journal of Allied Health Sciences and Practice, 18*(4). https://nsuworks.nova.edu/ijahsp

# Shai, D., & Belsky, J. (2017). Parental embodied mentalizing: How the nonverbal dance between parents and infants predicts children’s socio-emotional functioning. *Attachment and Human Development, 19*, 191–219. https://doi.org/10.1080/14616734.2016.1255653

# Shai, D., Dollberg, D., & Szepsenwol, O. (2017). The importance of parental verbal and embodied mentalizing in shaping parental experiences of stress and coparenting. *Infant Behavior and Development, 49,* 87–96. https://doi.org/10.1016/j.infbeh.2017.08.003

# Short, V. L., Gannon, M., Weingarten, W., Kaltenbach, K., LaNoue, M., & Abatemarco, D. J. (2017). Reducing stress among mothers in drug Tteatment: A description of a mindfulness based parenting intervention. Maternal and Child Health Journal, 21, 1377–1386. https://doi.org/10.1007/s10995-016-2244-1

# Singh, N. N., Lancioni, G. E., Karazsia, B. T., Myers, R. E., Hwang, Y. S., & Analayo, B. (2019). Effects of mindfulness-based positive behavior support (MBPBS) training are equally beneficial for mothers and their children with autism spectrum disorder or with intellectual disabilities. Frontiers in Psychology, 10. https://doi.org/10.3389/fpsyg.2019.00385

# Singh, N. N., Lancioni, G. E., Medvedev, O. N., Hwang, Y.-S., & Myers, R. E. (2020). A component analysis of the Mindfulness-Based Positive Behavior Support (MBPBS) Program for mindful parenting by mothers of children with Autism Spectrum Disorder. *Mindfulness, 12*(2), 463–475. https://doi.org/10.1007/s12671-020-01376-9

# Singh, N. N., Lancioni, G. E., Winton, A. S. W., Fisher, B. C., Wahler, R. G., McAleavey, K., Singh, J., & Sabaawi, M. (2006). Mindful parenting decreases aggression, noncompliance, and self-injury in children with autism. *Journal of Emotional and Behavioral Disorders, 14*, 169–177. https://doi.org/10.1177/10634266060140030401

# Singh, N. N., Lancioni, G. E., Winton, A. S. W., Karazsia, B. T., Myers, R. E., Latham, L. L., & Singh, J. (2014). Mindfulness-based positive behavior support (MBPBS) for mothers of adolescents with autism spectrum disorder: Effects on adolescents’ behavior and parental stress. *Mindfulness, 5,* 646–657. https://doi.org/10.1007/s12671-014-0321-3

# Singh, N. N., Lancioni, G. E., Winton, A. S. W., Singh, J., Curtis, W. J., Wahler, R. G., & McAleavey, K. M. (2007). Mindful parenting decreases aggression and increases social behavior in children with developmental disabilities. *Behavior Modification, 31,* 749–771. https://doi.org/10.1177/0145445507300924

# Singh, N. N., Singh, A. N., Lancioni, G. E., Singh, J., Winton, A. S. W., & Adkins, A. D. (2010). Mindfulness training for parents and their children with ADHD increases the children’s compliance*. Journal of Child and Family Studies, 19,* 157–166. https://doi.org/10.1007/s10826-009-9272-z

# Siu, A. F. Y., Ma, Y., & Chui, F. W. Y. (2016). Maternal mindfulness and child social behavior: The mediating role of the mother-child relationship. Mindfulness, 7, 577–583. https://doi.org/10.1007/s12671-016-0491-2

# Slade, A., Grienenberger, J., Bernbach, E., Levy, D., & Locker, A. (2005). Maternal reflective functioning, attachment, and the transmission gap: A preliminary study. Attachment and Human Development, 7, 283–298. https://doi.org/10.1080/14616730500245880

# Slade, A., Holland, M. L., Ordway, M. R., Carlson, E. A., Jeon, S., Close, N., … Sadler, L. S. (2018). Minding the Baby ®: Enhancing parental reflective functioning and infant attachment in an attachment-based, interdisciplinary home visiting program. *Development and Psychopathology, 32*(1), 123-137. https://doi.org/10.1017/S0954579418001463

# Slade, A., Holland, M. L., Ordway, M. R., Carlson, E. A., Jeon, S., Close, N., Mayes, L.C., & Sadler, L. S. (2020). Minding the Baby®: Enhancing parental reflective functioning and infant attachment in an attachment-based, interdisciplinary home visiting program. *Development and Psychopathology, 32,* 123-137. https://doi.org/10.1017/S0954579418001463

# Sleed, M., Baradon, T., & Fonagy, P. (2013). New Beginnings for mothers and babies in prison: A cluster randomized controlled trial. *Attachment and Human Development, 15*, 349–367. https://doi.org/10.1080/14616734.2013.782651

# Smaling, H. J.A., Huijbregts, S. C. J., Suurland, J., Van Der Heijden, K. B., Van Goozen, S. H. M., & Swaab, H. (2015). Prenatal reflective functioning in primiparous women with a high-risk profile. *Infant Mental Health Journal, 36,* 251–261. https://doi.org/10.1002/imhj.21506

# Smaling, H. J.A., Huijbregts, S. C. J., van der Heijden, K. B., Hay, D. F., van Goozen, S. H. M., & Swaab, H. (2017). Prenatal reflective functioning and development of aggression in infancy: The roles of maternal intrusiveness and sensitivity. *Journal of Abnormal Child Psychology, 45,* 237–248. https://doi.org/10.1007/s10802-016-0177-1

# Smaling, H. J.A., Huijbregts, S. C. J., van der Heijden, K. B., van Goozen, S. H. M., & Swaab, H. (2016). Maternal reflective functioning as a multidimensional construct: Differential associations with children’s temperament and externalizing behavior. *Infant Behavior and Development, 44*, 263–274. https://doi.org/10.1016/j.infbeh.2016.06.007

# Smaling, Hanneke J.A., Huijbregts, S. C. J., Suurland, J., van der Heijden, K. B., Mesman, J., van Goozen, S. H. M., & Swaab, H. (2016). Prenatal reflective functioning and accumulated risk as predictors of maternal interactive behavior during free play, the still-face paradigm, and two teaching tasks. *Infancy, 21,* 766–784. https://doi.org/10.1111/infa.12137

# Srivastava, M., Gupta, A., Talukdar, U., Kalra, B. P., & Lahan, V. (2011). Effect of parental training in managing the behavioral problems of early childhood. *Indian Journal of Pediatrics, 78, 973*–978. https://doi.org/10.1007/s12098-011-0401-5

# Stacks, A. M., Barron, C. C., & Wong, K. (2019). Infant mental health home visiting in the context of an infant—toddler court team: Changes in parental responsiveness and reflective functioning. *Infant Mental Health Journal, 40,* 523–540. https://doi.org/10.1002/imhj.21785

# Stacks, A. M., Muzik, M., Wong, K., Beeghly, M., Huth-Bocks, A., Irwin, J. L., & Rosenblum, K. L. (2014). Maternal reflective functioning among mothers with childhood maltreatment histories: Links to sensitive parenting and infant attachment security. *Attachment & Human Development, 16,* 515-533. https://doi.org/10.1080/14616734.2014.935452

# Staines, J., Golding, K., & Selwyn, J. (2019). Nurturing attachments parenting program: The relationship between adopters’ parental reflective functioning and perception of their children’s difficulties. Developmental Child Welfare, 1, 143–158. https://doi.org/10.1177/2516103219829861

# Stover, C. S., & Coates, E. E. (2016). The relationship of reflective functioning to parent child interactions in a sample of fathers with concurrent intimate partner violence perpetration and substance abuse problems. *Journal of Family Violence, 31,* 433–442. https://doi.org/10.1007/s10896-015-9775-x

# Stover, C. S., & Kiselica, A. (2014). An initial examination of the association of reflective functioning to parenting of fathers. *Infant Mental Health Journal, 35,* 452–461. https://doi.org/10.1002/imhj.21459

# Suardi, F., Moser, D. A., Sancho Rossignol, A., Manini, A., Vital, M., Merminod, G., ... & Schechter, D. S. (2020). Maternal reflective functioning, interpersonal violence-related posttraumatic stress disorder, and risk for psychopathology in early childhood. *Attachment & Human Development, 22,* 225-245. https://doi.org/10.1080/14616734.2018.1555602

# Suchman, N. E., DeCoste, C. L., McMahon, T. J., Dalton, R., Mayes, L. C., & Borelli, J. (2017). Mothering from the Inside Out: Results of a second randomized clinical trial testing a mentalization-based intervention for mothers in addiction treatment. Development and Psychopathology, 29, 617–636. https://doi.org/10.1017/S0954579417000220

# Suchman, N. E., DeCoste, C., Borelli, J. L., & McMahon, T. J. (2018). Does improvement in maternal attachment representations predict greater maternal sensitivity, child attachment security and lower rates of relapse to substance use? A second test of Mothering from the Inside Out treatment mechanisms. *Journal of Substance Abuse Treatment, 85,* 21–30. https://doi.org/10.1016/j.jsat.2017.11.006

# Suchman, N. E., DeCoste, C., Castiglioni, N., McMahon, T. J., Rounsaville, B., & Mayes, L. (2010). The mothers and toddlers program, an attachment-based parenting intervention for substance using women: Post-treatment results from a randomized clinical pilot. *Attachment and Human Development, 12,* 483–504. https://doi.org/10.1080/14616734.2010.501983

# Suchman, N. E., DeCoste, C., Leigh, D., & Borelli, J. (2010). Reflective functioning in mothers with drug use disorders: Implications for dyadic interactions with infants and toddlers. *Attachment and Human Development, 12,* 567–585. https://doi.org/10.1080/14616734.2010.501988

# Suchman, N. E., DeCoste, C., McMahon, T. J., Rounsaville, B., & Mayes, L. (2011). The Mothers and Toddlers program, an attachment-based parenting intervention for substance-using women: Results at 6-week follow-up in a randomized clinical pilot. *Infant Mental Health Journal, 32,* 427–449. https://doi.org/10.1002/imhj.20303

# Suchman, N. E., DeCoste, C., Rosenberger, P., & McMahon, T. J. (2012). Attachment-based intervention for substance-using mothers: A preliminary test of the proposed mechanisms of change. *Infant Mental Health Journal, 33*, 360–371. https://doi.org/10.1002/imhj.21311

# Suchman, N. E., Ordway, M. R., de las Heras, L., & McMahon, T. J. (2016). Mothering from the Inside Out: Results of a pilot study testing a mentalization-based therapy for mothers enrolled in mental health services. *Attachment and Human Development, 18,* 596–617. https://doi.org/10.1080/14616734.2016.1226371

# Suchman, N., DeCoste, C., Castiglioni, N., Legow, N., & Mayes, L. (2008). The Mothers and Toddlers program: Preliminary findings from an attachment-based parenting intervention for substance-abusing mothers. *Psychoanalytic Psychology, 25,* 499–517. https://doi.org/10.1037/0736-9735.25.3.499

# Townshend, K., Caltabiano, N. J., Powrie, R., & O’Grady, H. (2018). A preliminary study investigating the effectiveness of the Caring for Body and Mind in Pregnancy (CBMP) in reducing perinatal depression, anxiety and stress. Journal of Child and Family Studies, 27, 1556–1566. https://doi.org/10.1007/s10826-017-0978-z

# Turpyn, C. C., & Chaplin, T. M. (2016). Mindful parenting and parents’ emotion expression: Effects on adolescent risk behaviors. *Mindfulness, 7,* 246–254. https://doi.org/10.1007/s12671-015-0440-5

# Turpyn, C. C., Chaplin, T. M., Fischer, S., Thompson, J. C., Fedota, J. R., Baer, R. A., & Martelli, A. M. (2019). Affective neural mechanisms of a parenting-focused mindfulness intervention. Mindfulness, *12*(2), 392-404. https://doi.org/10.1007/s12671-019-01118-6

# Væver, M. S., Cordes, K., Stuart, A. C., Tharner, A., Shai, D., Spencer, R., & Smith-Nielsen, J. (2020). Associations of maternal sensitivity and embodied mentalizing with infant-mother attachment security at one year in depressed and non-depressed dyads. *Attachment & Human Development,* 1-18.

# van de Weijer-Bergsma, E., Formsma, A. R., de Bruin, E. I., & Bögels, S. M. (2012). The effectiveness of mindfulness training on behavioral problems and attentional functioning in adolescents with ADHD. *Journal of Child and Family Studies, 21*, 775–787. https://doi.org/10.1007/s10826-011-9531-7

# van den Heuvel, M. I., Johannes, M. A., Henrichs, J., & Van den Bergh, B. R. H. (2015). Maternal mindfulness during pregnancy and infant socio-emotional development and temperament: The mediating role of maternal anxiety. *Early Human Development, 91,* 103–108. https://doi.org/10.1016/j.earlhumdev.2014.12.003

# van der Oord, S., Bögels, S. M., & Peijnenburg, D. (2012). The effectiveness of mindfulness training for children with ADHD and mindful parenting for their parents. *Journal of Child and Family Studies, 21*, 139–147. https://doi.org/10.1007/s10826-011-9457-0

# van Gampelaere, C., Luyckx, K., Goethals, E. R., van der Straaten, S., Laridaen, J., Casteels, K., Vanbesien, J., Depoorter, S., Klink, D., Cools, M., & Goubert, L. (2020). Parental stress, anxiety and trait mindfulness: Associations with parent–child mealtime interactions in children with type 1 diabetes. *Journal of Behavioral Medicine, 43*(3), 448–459. https://doi.org/10.1007/s10865-020-00144-3

# Van Gampelaere, C., Luyckx, K., Van Ryckeghem, D. M. L., van der Straaten, S., Laridaen, J., Goethals, E. R., Casteels, K., Vanbesien, J., den Brinker, M., Cools, M., & Goubert, L. (2019). Mindfulness, worries, and parenting in parents of children with Type 1 Diabetes. *Journal of Pediatric Psychology, 44,* 499–508. https://doi.org/10.1093/jpepsy/jsy094

# Cowling, C., & Van Gordon, W. (2018). Effects of a brief online mindfulness intervention on mindfulness, psychological distress and parenting stress in preschool parents. *Mindfulness and Compassion, 3*, 55–70.

# Vismara, L., Sechi, C., & Lucarelli, L. (2020). Reflective function in first-time mothers and fathers: Association with infant temperament and parenting stress*. European Journal of Trauma & Dissociation*, 1-6. https://doi.org/10.1016/j.ejtd.2020.100147

# Waldman-Levi, A., Finzi-Dottan, R., & Cope, A. (2020). Mother-child Joint Play: The Role of Maternal Caregiving and Reflective Function. *Journal of Child and Family Studies, 29*, 94-104. https://doi.org/10.1007/s10826-019-01638-8

# Wang, H., Han, Z. R., Yan, J. J., & Ahemaitijiang, N. (2020). Dispositional mindfulness moderates the relationship between family risks and Chinese parents’ mental health. *Mindfulness, 12*(3), 672–682. https://doi.org/10.1007/s12671-020

# Wang, S. S.-C., & Lo, H. H. M. (2020). The role of mindful parenting in the relationship of parent and child mental health in Taiwan Chinese. *China Journal of Social Work, 13*(3), 232–249. https://doi.org/10.1080/17525098.2020.1815351

# Wang, Y., Liang, Y., Fan, L., Lin, K., Xie, X., Pan, J., & Zhou, H. (2018). The indirect path from mindful parenting to emotional problems in adolescents: The role of maternal warmth and adolescents’ mindfulness. *Frontiers in Psychology*, 1-7. https://doi.org/10.3389/fpsyg.2018.00546

# Warriner, S., Crane, C., Dymond, M., & Krusche, A. (2018). An evaluation of mindfulness-based childbirth and parenting courses for pregnant women and prospective fathers/partners within the UK NHS (MBCP-4-NHS). *Midwifery, 64*, 1–10. https://doi.org/10.1016/j.midw.2018.05.004

# Waters, L. (2016). The relationship between child stress, child mindfulness and parent mindfulness. *Psychology, 7*, 40–51. https://doi.org/10.4236/psych.2016.71006

# Weitlauf, A. S., Broderick, N., Stainbrook, J. A., Taylor, J. L., Herrington, C. G., Nicholson, A. G., Santulli, M., Dykens, E. M., Juárez, A. P., & Warren, Z. E. (2020). Mindfulness-Based Stress Reduction for parents implementing early intervention for autism: An RCT. *Pediatrics, 145,* 81–92. https://doi.org/10.1542/peds.2019-1895K

# Wheeler, A. C., Miller, S., Wylie, A., & Edwards, A. (2018). Mindfulness and acceptance as potential protective factors for mothers of children with fragile X syndrome. *Frontiers in Public Health, 6,* 1-10. https://doi.org/10.3389/fpubh.2018.00316

# Whitlock, J., Lloyd-Richardson, E., Fisseha, F., & Bates, T. (2018). Parental secondary stress: The often hidden consequences of nonsuicidal self-injury in youth. Journal of Clinical Psychology, 74, 178–196. https://doi.org/10.1002/jclp.22488

# Williams, K. L., & Wahler, R. G. (2010). Are mindful parents more authoritative and less authoritarian? An analysis of clinic-referred mothers. *Journal of Child and Family Studies, 19,* 230–235. https://doi.org/10.1007/s10826-009-9309-3

# Wilson, H., & Donachie, A. L. (2018). Evaluating the effectiveness of a dialectical behaviour therapy (DBT) informed programme in a community perinatal team. *Behavioural and Cognitive Psychotherapy, 46,* 541–553. https://doi.org/10.1017/S1352465817000790

# Wong, K., Hicks, L. M., Seuntjens, T. G., Trentacosta, C. J., Hendriksen, T. H. G., Zeelenberg, M., & van den Heuvel, M. I. (2019). The role of mindful parenting in individual and social decision-making in children. *Frontiers in Psychology, 10,* 1-10. https://doi.org/10.3389/fpsyg.2019.00550

# Wong, K., Stacks, A. M., Rosenblum, K. L., & Muzik, M. (2017). Parental reflective functioning moderates the relationship between difficult temperament in infancy and behavior problems in toddlerhood. *Merrill-Palmer Quarterly, 63*, 54–76. https://doi.org/10.13110/merrpalmquar1982.63.1.0054

# Yang, M., Jia, G., Sun, S., Ye, C., Zhang, R., & Yu, X. (2019). Effects of an Online Mindfulness Intervention focusing on attention monitoring and acceptance in pregnant women: A randomized controlled trial. *Journal of Midwifery and Women’s Health, 64*(1), 68–77. https://doi.org/10.1111/jmwh.12944

# Zarnegar, Z., Hambrick, E. P., Perry, B. D., Azen, S. P., & Peterson, C. (2016). Clinical improvements in adopted children with fetal alcohol spectrum disorders through neurodevelopmentally informed clinical intervention: A pilot study. *Clinical Child Psychology and Psychiatry, 21,* 551–567. https://doi.org/10.1177/1359104516636438

# Zeegers, M. A. J., Potharst, E. S., Veringa-Skiba, I. K., Aktar, E., Goris, M., Bögels, S. M., & Colonnesi, C. (2019). Evaluating mindful with your baby/toddler: Observational changes in maternal sensitivity, acceptance, mind-mindedness, and dyadic synchrony. *Frontiers in Psychology,* *10*, 1-14. https://doi.org/10.3389/fpsyg.2019.00753

# Zhang, D., Chan, S. K. C., Lo, H. H. M., Chan, C. Y. H., Chan, J. C. Y., Ting, K. T., … Wong, S. Y. S. (2017). Mindfulness-based intervention for Chinese children with ADHD and their parents: A pilot mixed-method study. *Mindfulness, 8,* 859–872. https://doi.org/10.1007/s12671-016-0660-3

# Zhang, N., Rudi, J. H., Zamir, O., & Gewirtz, A. H. (2018). Parent engagement in online mindfulness exercises within a parent training program for post-deployed military families. *Mindfulness, 9,* 725–736. https://doi.org/10.1007/s12671-017-0810-2

# Zhang, N., Zhang, J., & Gewirtz, A. H. (2019). Do less mindful mothers show better parenting via improvements in trait mindfulness following a military parent training program? *Frontiers in Psychology, 10,* 1-14. https://doi.org/10.3389/fpsyg.2019.00909

Zhang, W., Wang, M., & Ying, L. (2019). Parental mindfulness and preschool children’s emotion regulation: The role of mindful parenting and secure parent-child attachment. *Mindfulness, 10*(12), 2481-2491. https://doi.org/10.1007/s12671-019-01177-9.

# Zimmer-Gembeck, M. J., Kerin, J. L., Webb, H. J., Gardner, A. A., Campbell, S. M., Swan, K., & Timmer, S. G. (2019). Improved perceptions of emotion regulation and reflective functioning in parents: Two additional positive outcomes of parent-child interaction therapy. Behavior Therapy, 50, 340–352. https://doi.org/10.1016/j.beth.2018.07.002
